# Development of newly synthesised quinazolinone-based CDK2 inhibitors with potent efficacy against melanoma

**DOI:** 10.1080/14756366.2022.2036985

**Published:** 2022-02-09

**Authors:** Eman R. Mohammed, Ghada F. Elmasry

**Affiliations:** Department of Pharmaceutical Chemistry, Faculty of Pharmacy, Cairo University, Cairo, Egypt

**Keywords:** Quinazolinones, cell cycle, CDK2 inhibitors, melanoma, MDA-MB-435

## Abstract

Inhibiting Cyclin-dependent kinase 2 (CDK2) has been established as a therapeutic strategy for the treatment of many cancers. Accordingly, this study aimed at developing a new set of quinazolinone-based derivatives as CDK2 inhibitors. The new compounds were evaluated for their anticancer activity against sixty tumour cell lines. Compounds **5c** and **8a** showed excellent growth inhibition against the melanoma cell line MDA-MB-435 with GI% of 94.53 and 94.15, respectively. Cell cycle analysis showed that compound **5c** led to cell cycle cessation at S phase and G2/M phase revealing that CDK2 could be the plausible biological target. Thus, the most cytotoxic candidates **5c** and **8a** were evaluated *in vitro* for their CDK2 inhibitory activity and were able to display significant inhibitory action. The molecular docking study confirmed the obtained results. ADME study predicted that **5c** had appropriate drug-likeness properties. These findings highlight a rationale for further development and optimisation of novel CDK2 inhibitors.

## Graphical Abstract

**Figure:**
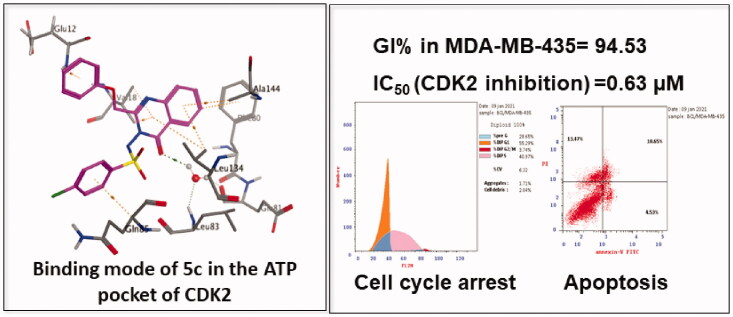

## 1. Introduction

Cancer prevalence and mortality are promptly increasing worldwide. The growing prominence of cancer; particularly with fast population growth and ageing in many parts of the world partially reveals a marked decline in mortality rates caused by coronary heart disease and stroke, with respect to cancer. Accordingly, cancer rapidly emerges to be the principal cause of death worldwide^1^.

Cyclin-dependent kinases (Cdks) are serine/threonine kinases. The mammalian cells contain 13 CDKs, among them CDK1, CDK2, CDK3, CDK4, and CDK6 which are involved in regulating the cell cycle checkpoints *via* initiation of proteinaceous substrates phosphorylation, using adenosine triphosphate (ATP)^2^^,^^3^. Abnormalities in the cell cycle regulation induce unrestricted cell growth, which is usually considered a hallmark of cancer^4^. Disruption of a deregulated cell cycle, as a result of overactivation of a CDK, has been accepted as a strategy for the treatment of proliferative diseases, especially cancer^5^.

Cyclin-dependent kinase 2 (CDK2) is a key regulator of the cell cycle transition and progression. CDK2 performs a critical role in controlling various events of the cell division cycle, such as DNA repair, gene transcription, G1-S transition, and modulation of G2 progression^6–8^. Additionally, the altered expression of cyclins whose association with CDKs is essential for their catalytic activity can drive aberrant proliferation in cancer. In the same vein, the overexpression of CDK2 regulatory subunits cyclin A and/or E is a key oncogenic process in many types of cancers^9^^,^^10^. Owing to the crucial role of CDK2 in cell cycle regulation, transcription activity, and tumours epigenetic modifications, CDK2 inhibition may confer a therapeutic benefit against certain cancers *viz* breast cancer^11^, colo-rectal cancer^12^^,^^13^, glioblastoma^14–16^, and melanoma^17^^,^^18^.

**Figure 1.**
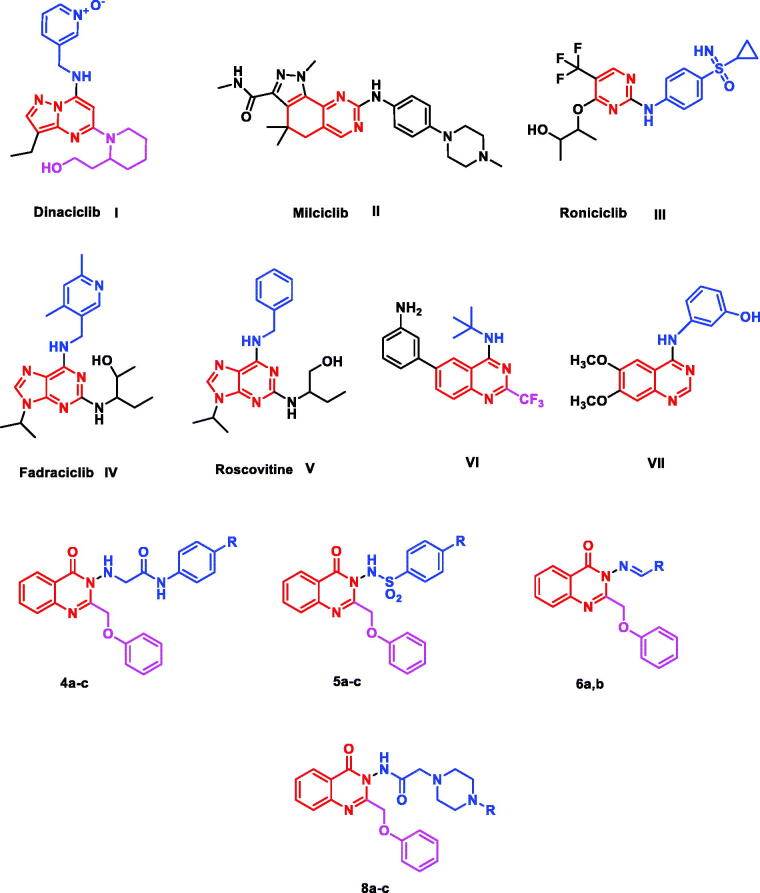
Reported CDK2 inhibitors and structures of the proposed compounds.

Several potent CDK2 inhibitors are under clinical evaluation, such as dinaciclib (I)^19^ (an inhibitor of several CDKs), milciclib (II)^20^, and roniciclib (III)^21^ (Figure 1). Nevertheless, some adverse effects have been observed during clinical trials, revealing that the discovery of new CDK2 inhibitors is still an active field of research^22^.

Surveying literature revealed that different scaffolds can possess CDK2 inhibitory activity, such as pyrazolopyrimidines (e.g. dinaciclib **I**), purines [e.g. fadraciclib **IV** and roscovitine (seliciclib) **V**]^23^^,^^24^ and quinazolines (e.g. compounds **VI** and **VII**)^25^^,^^26^ (Figure 1). Almost all of these are ATP- competitive inhibitors, interacting with Leu83 residue at the catalytic ATP binding site of the enzyme. Moreover, these CDK2 inhibitors share similar properties, such as hydrophobicity, low molecular weight, and heterocyclic flat structure. Interestingly, quinazolin-4(3*H*)-one is a privileged basic structure having promising anticancer activity with high human tolerance^27–29^.

In the course of finding several chemical substances which may serve as leads for developing new anticancer agents, we are particularly interested in the present study with quinazolinone derivatives in an attempt to achieve novel CDK2 inhibitors serving as chemotherapeutic agents. Continuing our studies on quinazolinone analogues as attractive candidates as antitumor agents^30^^,^^31^, we have designed some new quinazolinone derivatives containing phenoxy methyl group at position 2 and bearing different moieties which are known to enhance the antitumor activity attached to the 3-amino group *viz* un/substituted phenyl acetamides^32^, un/substituted benzene sulfonamides^33^, benzylidenes^34^, and acetyl piperazines^35^ (Figure 1). In this work, the substitution pattern at the 2 and 3 positions of the quinazolinone scaffold was selected to provide a different electronic environment that would affect the lipophilicity, and hence the activity of the target molecules. The aim of constructing these congeners is an attempt to attain active CDK2 inhibitors with potentiated activity against cancerous cells in which CDK2 is overexpressed.

All the newly synthesised quinazolinones were subjected to *in vitro* anticancer screening by the National Cancer Institute (USA) against 60 different human cell lines. The most potent compounds **5c** and **8a** were selected to be further studied through the determination of their half-maximal inhibitory concentration (IC_50_) values against melanoma MDA-MB-435 (**5c** and **8a**) and glioblastoma SNB-75 cell lines (**5c**). To investigate the mechanistic pathways of the anticancer activity of the most active representatives of this class, they were subjected to *in vitro* CDK2 inhibition assay. Furthermore, the effect of **5c** on the induction of apoptosis in MDA-MB-435 and SNB-75 cell lines was also investigated using flow cytometry and molecular docking study was carried out for the most active compound **5c** to identify the binding features required for its antitumor activity.

## 2. Experimental section

### 2.1. Chemistry

#### 2.1.1. General

Starting materials and solvents were purchased from commercial sources and were used without further purification. Melting points were determined by open capillary tube method using Electrothermal 9100 melting point apparatus (Staffordshire, UK) and were uncorrected. Elemental analyses and Mass spectra were carried out at the Regional Centre for Mycology and Biotechnology, Al-Azhar University, Egypt. Mass spectral data were given as *m/z* (relative abundance %). NMR and Infra-red spectra were performed at the Microanalytical Unit-Faculty of Pharmacy, Cairo University. NMR spectra were recorded on a Bruker Ascend 400/R (^1^H: 400, ^13^C: 100 MHz) spectrometer and were run in deuterated chloroform (CDCl_3_) or deuterated dimethylsulphoxide (DMSO‑*d*6). Chemical shift values (*δ*) were given in parts per million (ppm). Coupling constants were given in hertz (Hz) and spin multiplicities were given as s (singlet), d (doublet), dd (doublet of doublet), t (triplet), q (quartet), or m (multiplet). Infra-red spectra (IR) were recorded as Potassium bromide discs on Schimadzu FT-IR 8400S spectrophotometer (Kyoto, Japan) and expressed in wavenumber (*υ*_max_, cm^−1^). TLC was performed on TLC plates UV fluorescent silica gel (60 FZSU), using chloroform: methanol 9:1 as the eluting system, and the spots were visualised by the ultraviolet lamp. The starting compounds **1**^36^, **2**^37^^,^^38^, and the key intermediates **3**^37^^,^^38^ and **7**^37^^,^^38^ were prepared as reported.

#### 2.1.2. General procedure for the synthesis of compounds 4a–c and 5a–c

A mixture of equimolar amounts of the amino derivative **3** and the appropriate 2-chloro-*N*-phenylacetamide or *p*-substituted benzenesulfonyl chloride in dry DMF (10 mL) containing anhydrous potassium carbonate (1.5 mmol, 0.2 g) were refluxed for 20 h. The mixture was poured over crushed ice and the precipitated solid was filtered off, washed with water, dried, and recrystallized from the suitable solvent.

##### 2.1.2.1. 2-((4-oxo-2-(phenoxymethyl)quinazolin-3(4H)-yl)amino)-N-phenylacetamide 4a

Yield: 80%, m.p.: 135–137 °C, I.R (KBr, cm^−1^): *υ*_max_ 3271 (2NH), 3136 (CH aromatic), 2947 (CH aliphatic), 1678 (2C = O), 1600 (C = N), 1550 (C = C). ^1^H-NMR (400 MHz, CDCl_3_): *δ* 5.30 (s, 2H, CH_2_), 5.35 (s, 2H, OCH_2_), 7.03–7.10 (m, 5H, phenyl-H), 7.29–7.36 (m, 5H, phenyl-H), 7.48–7.55 (m, 3H, 1H phenyl-H + 2H NH exchanged by D_2_O), 7.80 (d, 2H, *J* = 4.00 Hz, phenyl-H), 8.32 (d, 1H, *J* = 8.00 Hz, phenyl-H). ^13^C-NMR: *δ* 47.7 (CH_2_ carbon), 53.4 (OCH_2_ carbon), 114.9, 119.8, 120.6, 121.9, 124.6, 126.5, 127.9, 128.9, 129.1, 129.7, 134.4, 146.5, 151.2 (Ar–Cs), 157.5 (C = N), 158.0 (C–O), 160.9 (C = O), 165.3 (C = O). MS *m/z* (%): 402.59 (M^+^ +2, 17.61%), 400.27 (M^+^, 20.49%), 370.95 (100%). Anal. Calcd. for C_23_H_20_N_4_O_3_ (400.44): C, 68.99; H, 5.03; N, 13.99. Found: C, 69.16; H, 5.21; N, 14,23.

##### 2.1.2.2. N-(4-methoxyphenyl)-2-((4-oxo-2-(phenoxymethyl)quinazolin-3(4H)-yl)amino)acetamide 4b

Yield: 90%, m.p.: 146–148 °C, I.R (KBr, cm^−1^): *υ*_max_ 3448, 3309 (2NH), 3062 (CH aromatic), 2931 (CH aliphatic), 1681 (2C = O), 1604 (C = N), 1500 (C = C). ^1^H-NMR (400 MHz, DMSO-d6): *δ* 3.35 (s, 3H, OCH_3_), 5.35 (s, 2H, CH_2_), 5.74 (s, 2H, OCH_2_), 6.91 (d, 1H, *J* = 8.80 Hz, phenyl-H), 6.96 (t, 1H, *J* = 7.32 Hz, phenyl-H), 7.05 (d, 2H, *J* = 8.00 Hz, phenyl-H), 7.31 (t, 2H, *J* = 7.32 Hz, phenyl-H), 7.53–7.58 (m, 3H, phenyl-H), 7.63 (d, 2H, *J* = 8.16 Hz, phenyl-H), 7.81 (t, 1H, *J* = 7.40 Hz, phenyl-H), 8.17 (d, 1H, *J* = 7.76 Hz, phenyl-H), 9.96 (s, 2H, NH exchanged by D_2_O). MS *m/z* (%): 432.08 (M^+^ +2, 31.71%), 430.82 (M^+^, 44.07%), 415.76 (100%). Anal. Calcd. for C_24_H_22_N_4_O_4_ (430.46): C, 66.97; H, 5.15; N, 13.02. Found: C, 67.13; H, 5.41; N, 13.30.

##### 2.1.2.3. N-(4-chlorophenyl)-2-((4-oxo-2-(phenoxymethyl)quinazolin-3(4H)-yl)amino)acetamide 4c

Yield: 80%, m.p.: 165–167 °C, I.R (KBr, cm^−1^): *υ*_max_ 3305 (2NH), 3062 (CH aromatic), 2974 (CH aliphatic), 1681 (2C = O), 1600 (C = N), 1492 (C = C), 752 (C–Cl). ^1^H-NMR (400 MHz, CDCl_3_): *δ* 5.30 (s, 2H, CH_2_), 5.34 (s, 2H, OCH_2_), 7.02–7.08 (m, 4H, phenyl-H), 7.18 (d, 1H, *J* = 8.56 Hz, phenyl-H), 7.30–7.40 (m, 3H, phenyl-H), 7.39 (d, 1H, *J* = 8.64 Hz, phenyl-H), 7.51–7.55 (m, 2H, 1H phenyl-H + 1H NH exchanged by D_2_O) , 7.78 (d, 2H, *J* = 8.20 Hz, phenyl-H), 8.29 (d, 1H, *J* = 7.96 Hz, phenyl-H), 8.88 (s, 1H, NH exchanged by D_2_O). ^13^C-NMR: *δ* 47.7 (CH_2_ carbon), 68.4 (OCH_2_ carbon), 114.9, 120.6, 120.9, 121.9, 122.1, 126.5, 127.4, 127.9, 128.9, 129.7, 134.3, 146.5, 151.5 (Ar–Cs), 157.5 (C = N), 158.0 (C–O), 160.9 (C = O), 165.3 (C = O). MS *m/z* (%): 435.53 (M^+^ +1, 27.64%), 434.59 (M^+^, 48.64%), 177.17 (100%). Anal. Calcd. for C_23_H_19_ClN_4_O_3_ (434.88): C, 63.52; H, 4.40; N, 12.88. Found: C, 63.29; H, 4.62; N, 13.14.

##### 2.1.2.4. N-(4-oxo-2-(phenoxymethyl)quinazolin-3(4H)-yl)benzenesulfonamide 5a

Yield: 70%, m.p.: 114–116 °C, I.R (KBr, cm^−1^): *υ*_max_ 3309 (NH), 3062 (CH aromatic), 2978 (CH aliphatic), 1681 (C = O), 1600 (C = N), 1492 (C = C), 1296, 1153 (SO_2_). ^1^H-NMR (400 MHz, CDCl_3_): *δ* 5.37 (s, 2H, OCH_2_), 7.01 (t, 1H, *J* = 6.00 Hz, phenyl-H), 7.08 (d, 1H, *J* = 8 Hz, phenyl-H), 7.30 (t, 1H, *J* = 8.00 Hz, phenyl-H), 7.48–7.58 (m, 4H, 3H phenyl-H + 1H NH exchanged by D_2_O), 7.77 (t, 1H, *J* = 8.00 Hz, phenyl-H), 7.86 (d, 1H, *J* = 8.00 Hz, phenyl-H), 7.96 (d, 5H, *J* = 8.00 Hz, phenyl-H), 8.29 (d, 1H, *J* = 8.00 Hz, phenyl-H). ^13^C-NMR: *δ* 67.9 (OCH_2_ carbon), 114.9, 120.2, 121.9, 126.7, 127.1, 127.7, 129.3, 129.7, 133.2, 134.6, 141.6 (Ar–Cs), 153.5 (C = N), 157.8 (C–O), 160.5 (C = O). MS *m/z* (%): 407.75 (M^+^, 54.00%), 250.15 (100%). Anal. Calcd. for C_21_H_17_N_3_O_4_S (407.44): C, 61.91; H, 4.21; N, 10.31. Found: C, 61.74; H, 4.34; N, 10.52.

##### 2.1.2.5. 4-methyl-N-(4-oxo-2-(phenoxymethyl)quinazolin-3(4H)-yl)benzenesulfonamide 5b

Yield: 75%, m.p.: 140–142 °C, I.R (KBr, cm^−1^): *υ*_max_ 3309 (NH), 3059 (CH aromatic), 2978 (CH aliphatic), 1681 (C = O), 1604 (C = N), 1492 (C = C), 1303, 1153 (SO_2_). ^1^H-NMR (400 MHz, CDCl_3_): *δ* 2.38 (s, 3H, CH_3_), 5.28, 5.32 (2 s, 2H, OCH_2_), 7.01 (t, 1H, *J* = 6.00 Hz, phenyl-H), 7.08 (d, 2H, *J* = 8.00 Hz, phenyl-H), 7.28–7.33 (m, 5H, 4H phenyl-H + 1H NH exchanged by D_2_O) , 7.51 (d, 1H, *J* = 4.00 Hz, phenyl-H), 7.76–7.81 (m, 4H, phenyl-H), 8.29 (d, 1H, *J* = 8.00 Hz, phenyl-H). ^13^C-NMR: *δ* 21.5 (CH_3_ carbon), 68.5 (OCH_2_ carbon), 114.9, 121.9, 126.6, 126.7, 127.2, 127.8, 134.6, 139.1, 143.9, 146.5, 151.5 (Ar–Cs), 157.2 (C = N), 158.1 (C–O), 160.9 (C = O). MS *m/z* (%): 421.84 (M^+^, 24.26%), 203.28 (100%). Anal. Calcd. for C_22_H_19_N_3_O_4_S (421.47): C, 62.70; H, 4.54; N, 9.97. Found: C, 62.59; H, 4.62; N, 10.15.

##### 2.1.2.6. 4-chloro-N-(4-oxo-2-(phenoxymethyl)quinazolin-3(4H)-yl)benzenesulfonamide 5c

Yield: 72%, m.p.: 208–210 °C, I.R (KBr, cm^−1^): *υ*_max_ 3329 (NH), 3086 (CH aromatic), 2931 (CH aliphatic), 1681 (C = O), 1602 (C = N), 1496 (C = C), 1373, 1153 (SO_2_), 752 (C–Cl). ^1^H-NMR (400 MHz, DMSO-d6): *δ* 5.22 (s, 2H, OCH_2_), 6.94–7.09 (m, 4H, phenyl-H), 7.35 (t, 3H, *J* = 8.00 Hz, phenyl-H), 7.52–7.57 (m, 3H, 2H-phenyl-H + 1H NH exchanged by D_2_O), 7.82 (br. s, 3H, phenyl-H), 8.31 (d, 1H, *J* = 8.00 Hz, phenyl-H). ^13^C-NMR: *δ* 68.3 (OCH_2_ carbon), 115.1, 115.3, 121.8, 121.9, 126.4, 127.4, 127.6, 129.8, 130.0, 135.0, 148.6 (Ar–Cs), 153.1 (C = N), 158.3 (C–O), 162.0 (C = O). MS *m/z* (%): 441.09 (M^+^, 50.22%), 239.67 (100%). Anal. Calcd. for C_21_H_16_ClN_3_O_4_S (441.06): C, 57.08; H, 3.65; N, 9.51. Found: C, 57.24; H, 3.71; N, 9.72.

#### 2.1.3. General procedure for the synthesis of compounds 6a and 6b

A mixture of equimolar amounts of compound **3** and the corresponding aromatic aldehyde was refluxed in glacial acetic acid (15 ml) for 8 h. The reaction mixture was poured over crushed ice and the precipitated solid was filtered off, washed with water, dried, and recrystallized from ethanol affording **6a** and **6b**, respectively.

##### 2.1.3.1. 3-((4-hydroxy-3-methoxybenzylidene)amino)-2-(phenoxymethyl)quinazolin-4(3H)-one 6a

Yield: 83%, m.p.: 191–193 °C, I.R (KBr, cm^−1^): *υ*_max_ 3300–3228 (OH), 3070 (CH aromatic), 2939 (CH aliphatic), 2839 (aldehydic CH), 1681 (C = O), 1600 (C = N), 1516 (C = C). ^1^H-NMR (400 MHz, CDCl_3_): *δ* 3.83 (s, 3H, OCH_3_), 5.35 (s, 2H, OCH_2_), 6.98–7.10 (m, 3H, phenyl-H), 7.31–7.44 (m, 4H, phenyl-H), 7.49–7.55 (m, 2H, phenyl-H), 7.62–7.64 (m, 1H, phenyl-H), 7.81 (s, 1H, phenyl-H), 8.87 (d, 1H, *J* = 4.80 Hz, phenyl-H), 9.85 (s, 1H, azomethine-H), 10.80 (s, 1H, OH exchanged by D_2_O). ^13^C-NMR: *δ* 56.1 (CH_3_O carbon), 68.4 (OCH_2_ carbon), 108.8, 114.4, 114.9, 121.3, 121.9, 126.8, 127.2, 127.5, 128.2, 129.5, 129.7, 134.4, 134.9, 147.2 (Ar–Cs), 150.1 (C = N), 151.7 (C = N), 163.4 (C–O), 190.9 (C = O). MS *m/z* (%): 401.27 (M^+^, 20.76%), 328.41 (100%). Anal. Calcd. for C_23_H_19_N_3_O_4_ (401.14): C, 68.82; H, 4.77; N, 10.47. Found: C, 68.65; H, 4.89; N, 10.64.

##### 2.1.3.2. 2-(phenoxymethyl)-3-((3-phenylallylidene)amino)quinazolin-4(3H)-one 6b

Yield: 85%, m.p.: 211–213 °C, I.R (KBr, cm^−1^): *υ*_max_ 3059 (CH aromatic), 2974 (CH aliphatic), 2850 (aldehydic CH), 1681 (C = O), 1600 (C = N), 1492 (C = C). ^1^H-NMR (400 MHz, CDCl_3_): *δ* 5.32 (s, 2H, OCH_2_), 7.00 (d, 2H, *J* = 8.64 Hz, phenyl-H), 7.04 (d, 2H, *J* = 7.76 Hz, phenyl-H), 7.28–7.32 (m, 3H, phenyl-H), 7.41–7.48 (m, 4H, phenyl-H), 7.53–7.56 (m, 2H, phenyl-H), 7.82 (m, 1H, =C–H), 8.33 (d, 1H, *J* = 7.84 Hz, =C–H), 8.87 (d, 1H, *J* = 9.44 Hz, phenyl-H), 9.71 (s, 1H, azomethine-H). ^13^C-NMR: *δ* 67.5 (OCH_2_ carbon), 115.3, 121.6, 123.9, 127.9, 129.5, 130.3, 131.3, 134.5, 135.0, 145.9, 146.7, 151.1, 152.9, 158.2, 158.7, 168.9, 176.1 (Ar–Cs), 193.9 (C = O). MS *m/z* (%): 381.93 (M^+^, 17.15%), 302.67 (100%). Anal. Calcd. for C_24_H_19_N_3_O_2_ (381.44): C, 75.57; H, 5.02; N, 11.02. Found: C, 75.38; H, 5.14; N, 11.25.

#### 2.1.4. General procedure for the synthesis of compounds 8a–c

Equimolar amounts of the chloro derivative **8** and the appropriate piperazine in dry DMF (10 ml) containing anhydrous potassium carbonate (1.5 mmol, 0.2 g), were refluxed for 20 h. The reaction mixture was cooled, poured over crushed ice and the yielded solid was crystallised from methanol to give the corresponding 8**a–c**.

##### 2.1.4.1. 2–(4-methylpiperazin-1-yl)-N-(4-oxo-2-(phenoxymethyl)quinazolin-3(4H)-yl)acetamide 8a

Yield: 65%, m.p.: 158–160 °C, I.R (KBr, cm^−1^): *υ*_max_ 3425 (NH), 3082 (CH aromatic), 2931 (CH aliphatic), 1681 (2C = O), 1620 (C = N), 1496 (C = C). ^1^H-NMR (400 MHz, DMSO-d6): 3.47–3.65 (m, 13H, 8H piperazinyl-H + 3H CH_3_ + 2H CH_2_), 5.03 (s, 2H, OCH_2_), 6.93–6.97 (m, 2H, phenyl-H), 7.23 (s, 2H, phenyl-H), 7.44–7.70 (m, 5H, 4H phenyl-H + 1H NH exchanged by D_2_O), 8.14 (s, 1H, phenyl-H). ^13^C-NMR: *δ* 44.2 (CH_3_ carbon), 45.0, 45.2 (piperazine-Cs), 45.5 (CH_2_ carbon), 72.4 (OCH_2_ carbon), 119.7, 126.5, 126.6, 131.0, 131.7, 134.4, 139.3 (Ar–Cs), 152.8 (C = N), 157.4 (C–O), 162.6 (C = O), 166.9 (C = O). MS *m/z* (%): 407.97 (M^+^, 67.35%), 386.95 (100%). Anal. Calcd. for C_22_H_25_N_5_O_3_ (407.47): C, 64.85; H, 6.18; N, 17.19. Found: C, 64.71; H, 6.27; N, 17.43.

##### 2.1.4.2. N-(4-oxo-2-(phenoxymethyl)quinazolin-3(4H)-yl)-2–(4-phenylpiperazin-1-yl)acetamid 8b

Yield: 70%, m.p.: 115–117 °C, I.R (KBr, cm^−1^): *υ*_max_ 3545 (NH), 3059 (CH aromatic), 2939 (CH aliphatic), 1720, 1693 (2C = O), 1600 (C = N), 1496 (C = C). ^1^H-NMR (400 MHz, CDCl_3_): *δ* 2.90 (s, 2H, CH_2_), 3.15–3.27 (m, 4H, piperazinyl-H), 3.29–3.38 (m, 4H, piperazinyl-H), 5.02 (d, 1H, *J* = 11.88 Hz, upfield H of OCH_2_), 5.15 (d, 1H, *J* = 11.88 Hz, downfield H of OCH_2_), 6.89–6.97 (m, 4H, phenyl-H), 7.03–7.07 (m, 2H, phenyl-H), 7.28–7.36 (m, 5H, 4H phenyl-H + 1H NH exchanged by D_2_O), 7.55 (t, 1H, *J* = 7.32 Hz, phenyl-H), 7.78–7.85 (m, 2H, phenyl-H), 8.29 (d, 1H, *J* = 7.64 Hz, phenyl-H). ^13^C-NMR: *δ* 48.1, 53.7 (piperazine-Cs), 60.8 (CH_2_ carbon), 68.2 (OCH_2_ carbon), 114.6, 116.2, 120.1, 121.9, 127.1, 127.9, 128.1, 129.2, 129.8, 135.1, 146.5, 150.9 (Ar–Cs), 152.1 (C = N), 158.0 (C–O), 159.7 (C = O), 170.2 (C = O). MS *m/z* (%): 469.48 (M^+^, 47.44%), 331.43 (100%). Anal. Calcd. for C_27_H_27_N_5_O_3_ (469.55): C, 69.07; H, 5.80; N, 14.92. Found: C, 68.95; H, 5.97; N, 15.11.

##### 2.1.4.3. 2–(4-(4-methoxyphenyl)piperazin-1-yl)-N-(4-oxo-2-(phenoxymethyl)quinazolin-3(4H)-yl)acetamide 8c

Yield: 70%, m.p.: 100–102 °C, I.R (KBr, cm^−1^): *υ*_max_ 3541 (NH), 3143 (CH aromatic), 2951 (CH aliphatic), 1720, 1693 (2C = O), 1598 (C = N), 1496 (C = C). ^1^H-NMR (400 MHz, CDCl_3_): *δ* 2.64 (s, 2H, piperazinyl-H), 2.90 (s, 2H, piperazinyl-H), 3.04 (s, 2H, piperazinyl-H), 3.33 (s, 2H, piperazinyl-H), 3.80 (s, 5H, 3H OCH_3_ + 2H CH_2_), 5.01 (d, 1H, *J* = 12.00 Hz, upfield H of OCH_2_), 5.14 (d, 1H, *J* = 12.00 Hz, downfield H of OCH_2_), 6.85–6.91 (m, 4H, phenyl-H), 7.02 (t, 3H, *J* = 8.00 Hz phenyl-H), 7.31 (t, 3H, *J* = 8.00 Hz, phenyl-H), 7.54 (t, 1H, *J* = 8.00 Hz, phenyl-H), 7.77–7.82 (m, 2H, 1H phenyl-H + 1H NH exchanged by D_2_O), 8.28 (d, 1H, *J* = 8.00 Hz, phenyl-H). ^13^C-NMR: *δ* 50.7, 53.8 (piperazine-Cs), 55.6 (OCH_3_ carbon), 60.8 (CH_2_ carbon), 68.2 (OCH_2_ carbon), 114.5, 114.6, 118.3, 121.9, 127.1, 127.9, 128.1, 129.8, 135.1, 145.2, 146.5 (Ar–Cs), 152.1 (C–O), 154.0 (C = N), 158.0 (C-O), 159.7 (C = O), 170.2 (C = O). MS *m/z* (%): 499.66 (M^+^, 38.98%), 324.50 (100%). Anal. Calcd. for C_28_H_29_N_5_O_4_ (499.57): C, 67.45; H, 6.09; N, 14.28. Found: C, 67.32; H, 5.85; N, 14.02.

### 2.2. Biological evaluation

#### 2.2.1. Biological assays

All experimental procedures applied in the mentioned biological assays were conducted as reported earlier; NCI-60 human tumour cell lines screening^39–41^, determination of IC_50_ in MDA-MB-435 and SNB-75 cell lines^42^^,^^43^, Cell cycle analysis^44^, Apoptosis determination^45^, CDK2 inhibition assay^46^ and have been provided in the Supplementary Materials.

#### 2.2.2. Statistical analysis

The presented data and results were confirmed by at least three independent experiments. The data have been expressed as means ± *SD* and analysed with GraphPad Prism 8.0 software. Statistical comparisons were made by Student’s *t*-test. *p*-Values <0.05 (*p* < 0.05) was considered statistically significant.

### 2.3. In silico studies

#### 2.3.1. Molecular docking study

The molecular docking study of the most active compound was achieved using Molecular Operating Environment (MOE, 2010.10) software. The X-ray crystal structure of CDK2 enzyme co-crystallised with the quinazoline derivative DIN-234325 as an inhibitor (PDB code: 2B53) was downloaded from the protein data bank^25^^,^^47^. The enzyme was prepared for docking by removal of all ligands and water molecules that are not involved in the binding. One conserved water molecule involved in the co-crystalized ligand binding to the CDK2 binding site was kept (Figure 5). The protein structure was prepared for docking study using Protonate 3 D protocol in MOE with default options. The Triangle Matcher placement method and London dG scoring function were used for the evaluation of the binding pattern and binding affinity of the ligands. Docking protocol was first validated by redocking of the co-crystallised ligand (DIN-234325) in the active site of the enzyme with an energy score (*S*) = −18.24 kcal/mol and root mean square deviation (RMSD) of 0.485 Å. The validated docking protocol was then used to study the ligand-target interactions in the active site for the most potent newly synthesised compounds **5c** to predict its binding mode and binding affinity to justify its promising potency.

#### 2.3.2. Drug likeness properties

The free Swiss ADME web tool available from the Swiss Institute of Bioinformatics (SIB) was used for the calculation of the physicochemical descriptors as well as to predict the ADME parameters, pharmacokinetic properties, and drug-like nature of the most active newly synthesised candidate **5c**^48–50^. The SMILES of the tested compound were inserted directly on the webpage followed by running the prediction process.

## 3. Results and discussion

### 3.1. Chemistry

**Scheme 1.**
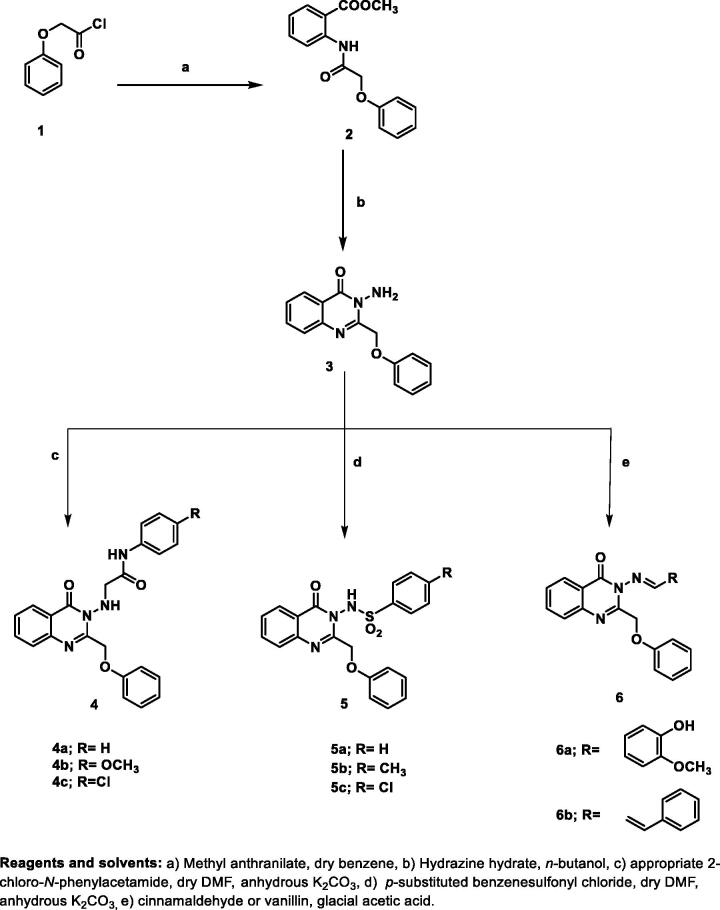
Preparation of the key starting compound **3** and the target compounds **4a–c, 5a–c**, and **6a,b**. Reagents and solvents: (a) Methyl anthranilate, dry benzene, (b) Hydrazine hydrate, *n*-butanol, (c) appropriate 2-chloro-*N*-phenylacetamide, dry DMF, anhydrous K_2_CO_3_, (d) p-substituted benzenesulfonyl chloride, dry DMF, anhydrous K_2_CO_3_, (e) cinnamaldehyde or vanillin, glacial acetic acid.

**Scheme 2.**
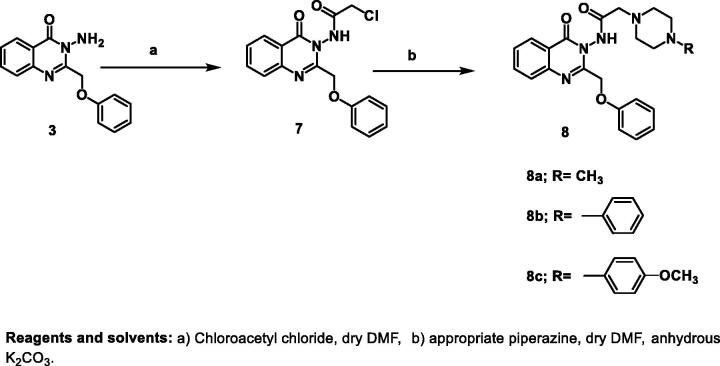
Synthesis of the target derivatives **8a–c**. Reagents and solvents: (a) Chloroacetyl chloride, dry DMF, (b) appropriate piperazine, dry DMF, anhydrous K_2_CO_3_.

The target quinazolin-4(3*H*)-one derivatives were synthesised as outlined in Schemes 1, 2. The starting compounds **1**^36^, **2**^37^^,^^38^, and the key intermediates **3**^37^^,^^38^ and **7**^37^^,^^38^ were prepared according to the reported procedures. As depicted in Scheme 1, stirring of 3-amino-2-(phenoxymethyl)quinazolin-4(3*H*)-one **3** with the appropriate 2-chloro-*N*-phenylacetamide or various 4-substituted benzene sulphonyl chloride in dry Dimethyl formamide at 70 °C in the presence of anhydrous Potassium carbonate afforded compounds **4a–c** and **5a–c**, respectively. The chemical structures of the new phenyl acetamide derivatives **4a–c** were confirmed by the appearance of an additional singlet signal in ^1^H-NMR spectra around 5.30 ppm assigned to the new methylene group. Furthermore, the ^1^H-NMR showed characteristic signals in the aromatic region attributed to the additional aromatic protons in compounds **4a–c** and **5a–c**. ^13^C NMR also illustrated the carbon skeleton of the target derivatives. Moreover, condensation of the key intermediate **3** with aromatic aldehydes, namely vanillin, and cinnamaldehyde in refluxing glacial acetic acid yielded the corresponding Schiff’s bases **6a,b**. ^1^H NMR spectra of **6a,b** illustrated the azomethine proton as a sharp singlet signal at 9.71–9.85 ppm. Additionally, acylation of **3** with chloroacetyl chloride in dry Dimethylformamide produced the chloroacetylamino derivative **7**, the key intermediate in Scheme 2. The latter upon reaction with different substituted piperazines in dry Dimethylformamide in the presence of anhydrous Potassium carbonate experienced aromatic nucleophilic substitution reaction and yielded **8a–c**. The postulated structures of the products were proved by the appearance of aliphatic signals at *δ* 3.04–3.48 ppm region ascribed to the piperazine protons. The ^13^C NMR spectra showed two signals at the range of 159.7–170.2 ppm corresponding to the two carbonyl carbons.

### 3.2. Biological evaluation

#### 3.2.1. NCI-60 human tumour cell lines screening

In this research, all of the newly synthesised quinazolinones were subjected to *in vitro* anticancer screening by the National Cancer Institute (USA) against 60 different human cell lines including leukaemia, non-small cell lung cancer, colon cancer, CNS cancer, melanoma, ovarian cancer, renal cancer, prostate cancer, and breast cancer^51^. The tested compounds were evaluated at a single dose (10^−5 ^M). The growth inhibition percentages obtained from the single-dose test for the tested compounds are shown in Tables 1, 2. Screening the anticancer activity of the tested compounds revealed that compounds **5c** and **8a** were the most active compounds displaying superior activity against the melanoma cell line MDA-MB-435 with growth inhibition percentages 94.53 and 94.15, respectively. Concerning the phenyl acetamide derivatives **4a–c**, Compound **4a** possessing the unsubstituted phenyl ring showed good potency against glioblastoma cell line SNB-75 with growth inhibition percentages 62.34. Regarding the benzenesulfonamide counterparts, the introduction of an electron-withdrawing group on the benzenesulfonamide moiety increased the inhibitory activity on the glioblastoma cell line SNB-75 as depicted by the chloro derivative **5c** (GI% = 63.09) which was higher than that of the unsubstituted derivative **5a** (GI% = 40.06) and the methyl derivative **5b** (GI% = 33.03). The chloro derivative **5c** also displayed high to moderate cytotoxicity against various carcinoma cell lines *viz* breast cancer cell lines MDA-MB-468 and MCF-7, leukaemia cell lines K-562 and SR, and colon cancer cell lines HCT-15 and KM12 with growth inhibition percentages 59.61, 58.11, 52.75 and 42.42, 45.60 and 44.77, sequentially. It is worth mentioning that the quinazolinone **5c** is not only the most active member among this series but also displayed the highest activity among all the target compounds. Interestingly, the piperazine derivative **8a** showed potent to moderate cytotoxicity against leukaemia cell lines SR and K-562, breast cancer cell lines MCF-7 and MDA-MB-468 and colon cancer cell lines HCT-15, KM12, and SW-620 with growth inhibition percentages 69.55, 67.80, 57.64, and 49.06, 44.04, 55.03, and 42.80, respectively. Other tested compounds exhibited weak activity against most investigated cell lines. Thus, compounds **5c** and **8a** were subjected to further testing to determine their IC_50_ against the most sensitive cell lines.

**Table 1.** Growth inhibition percent obtained from the single dose (10^−5 ^M) test for the target quinazolinones **4a–c** and **5a–c**.

Panel/cell line	GI% at 10^−5^ M of the quinazolinones					
	4a	4b	4c	5a	5b	5c
	827118	827119	827120	827121	827122	827123
Leukaemia						
CCRF-CEM	–	–	–	–	–	–
HL-60(TB)	–	–	–	–	–	24.32
K-562	–	–	10.43	–	–	**52.75**
MOLT-4	–	–	15.9	–	–	30.51
RPMI-8226	–	–	–	–	–	–
SR	–	–	10.05	–	–	**42.42**
Non-small cell lung cancer						
A549/ATCC	–	–	–	–	–	14.05
EKVX	14.38	10.17	19.01	15.21	13.18	25.19
HOP-62	13.59	12.71	12.12	16.59	11.16	25.00
HOP-92	15.94	16.34	29.33	10.25	10.82	N.T.
NCI-H226	–	–	11.13	–	–	17.00
NCI-H23	16.55	–	–	11.73	11.47	17.19
NCI-H322M	–	–	–	–	–	17.13
NCI-H460	–	–	–	–	–	–
NCI-H522	13.74	12.54	24.21	–	–	30.63
Colon cancer						
COLO 205	–	–	–	–	–	–
HCC-2998	–	–	–	–	–	22.36
HCT-116	–	–	–	–	–	16.40
HCT-15	–	–	–	–	–	**45.60**
HT29	–	–	–	–	–	20.01
KM12	–	–	–	–	–	**44.77**
SW-620	–	–	–	–	–	30.94
CNS cancer						
SF-268	–	–	–	–	–	11.40
SF-295	–	–	–	–	–	–
SF-539	–	–	–	–	–	13.14
SNB-19	–	–	–	–	–	20.21
SNB-75	**62.34**	21.9	–	**40.06**	**33.03**	**63.09**
U251	–	–	–	–	–	12.24
Melanoma						
LOX IMVI	–	–	–	–	–	16.40
MALME-3M	–	–	12.02	–	–	17.90
M14	–	–	–	–	–	25.84
MDA-MB-435	–	–	–	–	–	**94.53**
SK-MEL-2	–	–	–	–	–	25.29
SK-MEL-28	–	–	–	–	–	12.91
SK-MEL-5	–	–	–	–	–	–
UACC-257	–	–	–	–	–	–
UACC-62	–	–	14.58	–	–	23.06
Ovarian cancer						
IGROV1	19.54	6.78	19.62	16.06	21.58	38.26
OVCAR-3	–	–	–	–	–	–
OVCAR-4	–	–	11.8	–	–	–
OVCAR-5	–	–	–	–	–	–
OVCAR-8	–	–	–	–	–	–
NCI/ADR-RES	–	–	10.99	–	–	26.99
SK-OV-3	N.T.	N.T.	4.95	N.T.	−4.74	N.T.
Renal cancer						
786-0	–	–	–	–	–	–
A498	–	34.65	16.35	–	–	36.68
ACHN	–	–	–	11.23	15	–
CAKI-1	12.78	21.63	23.58	15.07	–	26.93
RXF 393	–	–	–	–	10.27	–
SN12C	13.08	12.37	19.15	11.46	–	23.41
TK-10	–	–	–	–	23.64	–
UO-31	21.57	15.77	10.43	18.13	10.74	37.25
Prostate cancer						
PC-3	10.95	9.76	15.9	–	–	17.26
DU-145	–	–	–	–	15.73	–
Breast cancer						
MCF7	15.7	13.20	10.05	14.31	11.54	**58.11**
MDA-MB-231/ATCC	18.56	11.38	–	13.77	10.94	32.6
HS 578T	11.86	–	19.01	–	–	24.96
BT-549	–	–	12.12	–	–	–
T-47D	–	–	29.33	–	–	25.18
MDA-MB-468	–	–	11.13	–	–	**59.61**

N.T.: not tested; – means GI < 10%.

Bold numbers represent high GI%.

**Table 2.** Growth inhibition percent obtained from the single dose (10^−5 ^M) test for the target quinazolinones **6a,b** and **8a–c**.

Panel/cell line	GI% at 10^−5^ M of the quinazolinones				
	6a	6b	8a	8b	8c
	827124	827125	827977	827126	827127
Leukaemia					
CCRF-CEM	–	15.66	10.65	–	–
HL-60(TB)	–	–	19.74	–	–
K-562	–	–	**67.8**	–	–
MOLT-4	–	–	11.86	–	–
RPMI-8226	–	12.52	23.17	–	–
SR	–	14.34	**69.55**	–	12.84
Non-small cell lung cancer					
A549/ATCC	–	–	–	–	–
EKVX	–	13.78	13.93	–	–
HOP-62	–	–	–	–	–
HOP-92	–	–	–	–	–
NCI-H226	–	–	15.39	–	–
NCI-H23	–	–	–	10.01	–
NCI-H322M	–	–	–	–	–
NCI-H460	–	–	–	–	–
NCI-H522	16.82	18.6	24.6	11.45	10.67
Colon cancer					
COLO 205	–	–	15.89	–	–
HCC-2998	–	–	10.06	–	–
HCT-116	–	–	18.15	–	–
HCT-15	–	23.15	**44.04**	–	–
HT29	–	–	26.89	–	–
KM12	–	–	**55.03**	–	–
SW-620	–	–	**42.80**	–	–
CNS cancer					
SF-268	–	–	–	–	–
SF-295	–	–	–	–	–
SF-539	–	–	11.37	–	–
SNB-19	–	17.13	13.65	–	–
SNB-75	13.12	16.57	27.37	–	26.47
U251	–	–	18.55	–	–
Melanoma					
LOX IMVI	–	27.86	14.73	–	–
MALME-3M	–	–	20.47	–	–
M14	–	–	21.06	–	–
MDA-MB-435	–	–	**94.15**	–	–
SK-MEL-2	–	–	12.84	–	–
SK-MEL-28	–	–	–	–	–
SK-MEL-5	–	–	17.11	–	–
UACC-257	–	–	–	–	–
UACC-62	–	–	30.69	–	–
Ovarian cancer					
IGROV1	–	–	26.87	–	–
OVCAR-3	–	–	–	–	–
OVCAR-4	–	10.01	–	–	–
OVCAR-5	–	–	–	–	–
OVCAR-8	–	–	–	–	–
NCI/ADR-RES	–	–	28.41	–	–
SK-OV-3	N.T.	N.T.	22.99	N.T.	N.T.
Renal cancer					
786-0	–	–	–	–	–
A498	–	–	18.24	–	15.04
ACHN	–	–	–	–	–
CAKI-1	–	–	28.85	–	–
RXF 393	–	–	–	–	–
SN12C	10.37	12.39	–	–	–
TK-10	–	–	–	–	–
UO-31	15.75	10.2	29.32	20.18	10.03
Prostate cancer					
PC-3	–	10.39	–	–	14.02
DU-145	–	–	–	–	–
Breast cancer					
MCF7	22.94	16.22	**57.64**	–	–
MDA-MB-231/ATCC	10.92	–	21.65	10.38	–
HS 578T	–	–	13.94	10.4	–
BT-549	–	–	–	–	–
T-47D	–	–	32.2	–	–
MDA-MB-468	–	10.89	**49.06**	–	–

N.T.: not tested; – means GI < 10%.

Bold numbers represent high GI%.

##### 3.2.1.1. Detection of IC_50_ in melanoma MDA-MB-435, glioblastoma SNB-75 cells, and normal cell line WI-38

Compounds **5c** and **8a** which displayed superior inhibitory activity against the melanoma cell line were selected to be further studied through the determination of its half-maximal inhibitory concentration (IC_50_) values against the most sensitive cancer cell lines compared to roscovitine as a reference anticancer drug. The results of the mean values of experiments performed in triplicate were summarised in Table 3. The *in vitro* results confirmed that the most sensitive cell line for compound **5c** was melanoma cell line MDA-MB-435 with IC_50_ = 3.03 µM which is 1.7-folds more potent than roscovitine (IC_50_ = 5.26 µM). Compound **8a** also showed good potency against MDA-MB-435 cell line with IC_50_ = 6.22 µM approaching the activity of roscovitine. Additionally, the quinazolinone **5c** exhibited promising anticancer activity against glioblastoma (SNB-75) cell line with IC_50_ value of 8.62 µM which is nearly equipotent as roscovitine (IC_50_ = 7.76 µM).

**Table 3.** Concentrations required for 50% inhibition of cell viability (IC_50_) of compound **5c** compared to roscovitine.

Panel/cell line		IC_50_ (µM)±SD*		
		5c	8a	Roscovitine
Melanoma	MDA-MB-435	3.03 ± 0.38	6.22 ± 0.53	5.26 ± 0.34
Glioblastoma	SNB-75	8.62 ± 0.13	–	7.76 ± 0.23

– means not determined.

*All results were found to be statistically significant at *p* *<* 0.05.

Furthermore, **5c** and **8a** were evaluated for their cytotoxic activity in normal fibroblast cells WI-38 to explore the compounds’ selectivity towards tumour cells. As shown in Table 4, the tested compounds were found to be more selective to the cancer cells. Interestingly, the quinazolinone **5c** showed superior selectivity towards melanoma MDA-MB-435 cell line with the highest selectivity index (SI = 20.63).

**Table 4.** Cytotoxic activity of compounds **5c, 8a** and roscovitine against WI-38 normal cells and their selectivity index (SI).

Compound No.	IC_50_ (µM) ± *SD*	SI	
	WI-38	MDA-MB-435	SNB-75
**5c**	62.52 ± 3.5	20.63	7.25
**8a**	37.27 ± 2.1	7.08	–
Roscovitine	14.01 ± 0.79	2.66	1.93

– means not determined.

Selectivity index (SI) was attained by dividing the activity of the tested compound (IC_50_) in normal cell line (WI-38) by its activity (IC_50_) in cancer cell line.

#### 3.2.2. Flow cytometric analysis and apoptotic studies

Compound **5c** showed the highest antiproliferative activity against MDA-MB-435 and SNB-75 cancer cell lines; consequently, flow cytometric analysis was carried out to study its effect on the cell cycle phases in the same cancer cell lines and thus identify its plausible biological target. Besides, apoptotic activity was measured by Annexin V-based flow cytometric analysis.

##### 3.2.2.1. Cell cycle analysis

**Figure 2.**
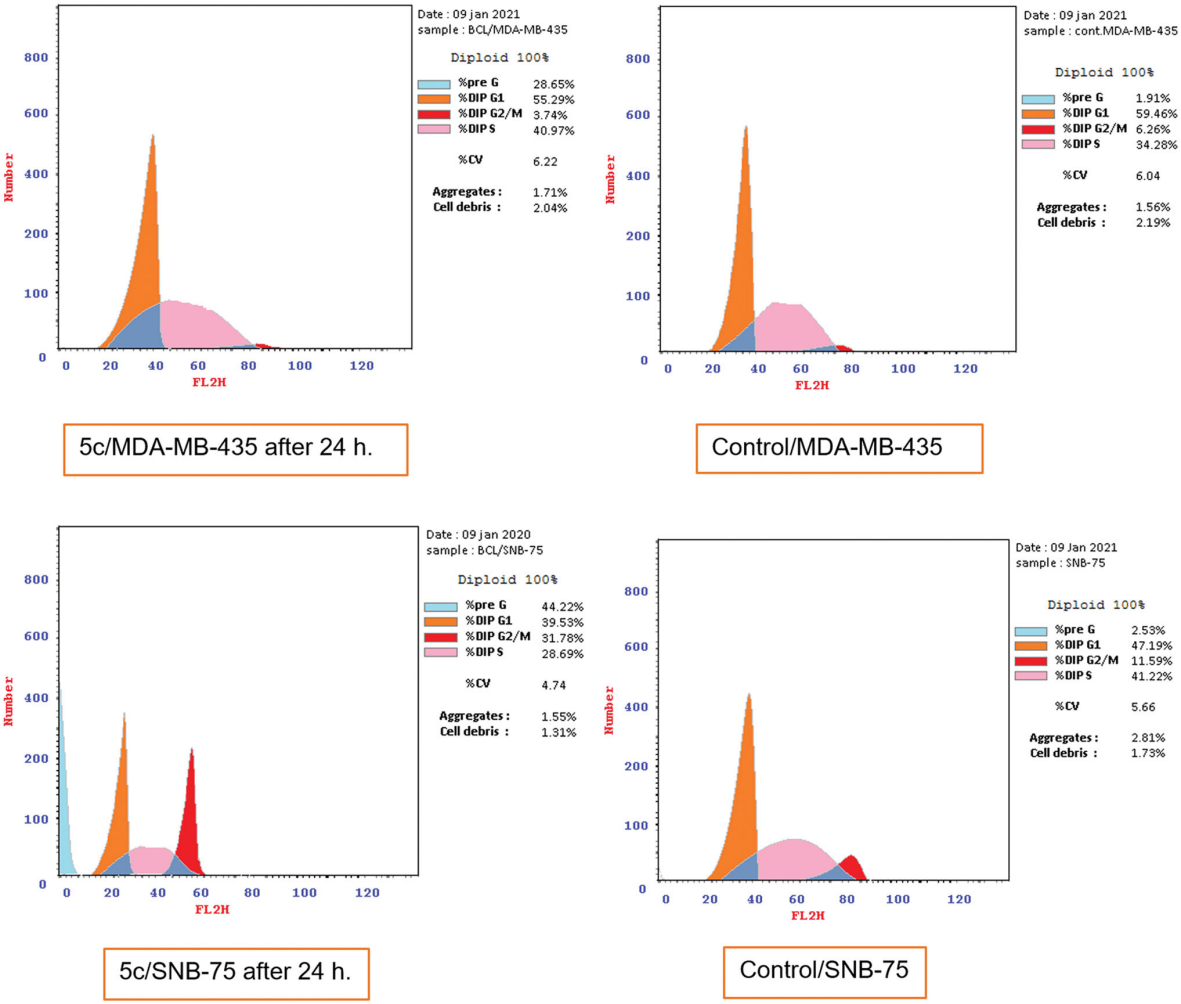
Effect of compound **5c** on the cell cycle of melanoma MDA-MB-435 and glioblastoma SNB-75 cell lines.

The role of CDK2 inhibitors in cell cycle arrest and apoptosis induction is well-documented^5^^,^^52^^,^^53^. The chloro quinazolinone **5c** that showed promising anticancer activity against MDA-MB-435 and SNB-75 cell lines was subjected to cell cycle analysis and apoptotic assay to define the phases at which cell cycle arrest takes place in both MDA-MB-435 and SNB-75 cell lines and to investigate its effect on induction of apoptosis. The results of the cell cycle analysis of compound **5c** are shown in Table 5 and Figure 2.

**Table 5.** Cell cycle analysis results of compound **5c** after 24 h in MDA-MB-435 and SNB-75 cell lines.

Cell line	Compound code	%G0-G1	%S	%G2/M	%Pre-G1	Comment
MDA-MB-435	5c	55.29	40.97	3.74	28.65	Pre G1 apoptosis and cell growth arrest at S
MDA-MB-435	Control	59.46	34.28	6.26	1.91	Control pattern
SNB-75	5c	39.53	28.69	31.78	44.22	Pre G1 apoptosis and cell growth arrest at G2/M
SNB-75	Control	47.19	41.22	11.59	2.53	Control pattern

The obtained results revealed that compound **5c** led to pre G1 apoptosis with a cell growth arrest at S phase in melanoma MDA-MB-435 cells after 24 h. which was confirmed by an increase in the percentage of DNA content (40.97%) after addition of the compound compared to control cells (34.28%). For glioblastoma SNB-75 cells, the results showed that the quinazolinone **5c** induced pre G1 apoptosis with a cell growth arrest at G2/M phase which was confirmed by an increase in the percentage of DNA content (31.78%) after the addition of the compound compared to the to that of the untreated control cells (11.59%). Statistical analysis of the different stages of the cell cycle demonstrated the ability of **5c** to induce significant levels of pre G1 apoptosis and cell cycle arrest in S phase and G2/M compared to the control. Thus, cell cycle arrest induced by the quinazolinone **5c** at S phase and G2/M phase suggests that CDK2 could be the biological target for the newly synthesised compounds.

##### 3.2.2.2. Apoptosis determination

**Figure 3.**
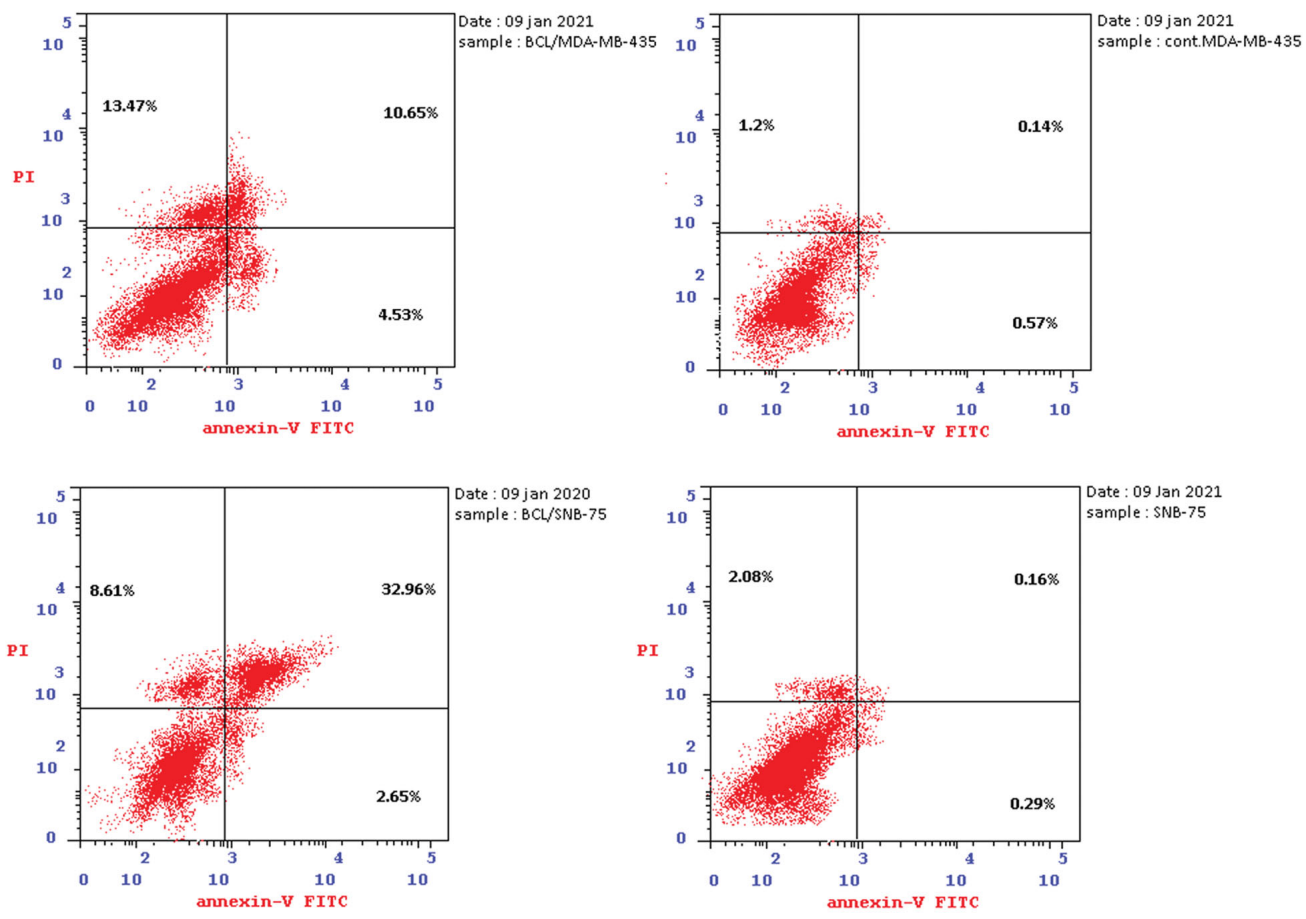
Effect of compound **5c** on induction of apoptosis in MDA-MB-435 and SNB-75 cancer cells using PI/Annexin double staining.

The apoptotic activity of quinazolinone **5c** in melanoma MDA-MB-435 and glioblastoma SNB-75 cell lines was further studied using Annexin V-FITC assay. As shown in Table 6 and Figure 3, total apoptotic cells percentage was increased in both cell lines (28.65, 44.22%, respectively), compared to the positive control, annexin V (% apoptosis 1.91, 2.53) after 24 h of treatment of MDA-MB-435 and SNB-75 cell lines with **5c** at its IC_50_ concentration. Statistical analysis of the different stages of apoptosis revealed the ability of **5c** to induce significant levels of apoptosis in MDA-MB-435 and SNB-75 treated cells (in all stages) compared to the control.

**Table 6.** Apoptosis induction analysis for compound **5c**.

Cell line	Compound code	%Apoptosis			
		Total	Early	Late	% Necrosis
MDA-MB-435	5c	28.65	4.53	10.65	13.47
MDA-MB-435	Control	1.91	0.57	0.14	1.2
SNB-75	5c	44.22	2.65	32.96	8.61
SNB-75	Control	2.53	0.29	0.16	2.08

#### 3.2.3. In vitro CDK2 inhibitory activity

It was reported that CDK2 is overexpressed in melanoma^44^^,^^45^. Accordingly, the most potent congeners on MDA-MB-435 the melanoma cancer cell line; **5c** and **8a** were selected as representatives to study the CDK2 inhibitory activity of the newly synthesised compounds. The quinazolinones **5c** and **8a** were further investigated *in vitro* for their kinase inhibitory activity on CDK2 and were compared to the CDK2 kinase inhibitor roscovitine as a reference standard. The results were presented in Table 7. The anti-proliferative activity of the quinazolinone **5c** appeared to correlate well with its ability to inhibit CDK2 at sub-micromolar range with IC_50_ value 0.63 µM which is twice as potent as roscovitine (IC_50_ = 1.28 µM). Additionally, Compound **8a** exhibited IC_50_ value equal to 1.74 µM which is slightly higher than that of the reference drug.

**Table 7.** *In vitro* inhibitory activity of compound **5c** against CDK2.

Compound	IC_50_ (µM) ± SD*
**5c**	0.63 ± 0.022
**8a**	1.74 ± 0.062
**Roscovitine**	1.28 ± 0.045

*All results were found to be statistically significant at *p* *<* 0.05.

### 3.3. In silico studies

#### 3.3.1. Molecular docking study

A molecular docking technique was performed to interpret the observed promising activity of compound **5c** on CDK2. Therefore, docking of **5c** into the three-dimensional X-ray crystallographic structure of the cyclin-dependent kinase2 (PDB code: 2B53)^25^ in complex with the quinazoline DIN-234325 (IC_50_ = 0.6 ± 0.1 µM) was carried out using Molecular Operating Environment (MOE) 2010.10. The inhibitor binds into the ATP binding pocket located in the deep cleft between the upper and lower domains of CDK2. Through two hydrogen bond interactions, the N1 of the quinazoline ring interacts with the key amino acids Glu81 and Leu83 *via* a water molecule. It is worth mentioning that the two previously mentioned interactions are consistently observed in the hinge region for ATP-competitive CDK2 inhibitors^54^^,^^55^. Stacking interactions were shaped through the quinazoline ring of the ligand and Val18 and Leu134 residues. Additionally, the aniline ring attached at position 6 of the quinazoline ring forms pi staking interaction with Gln 85.

**Figure 4.**
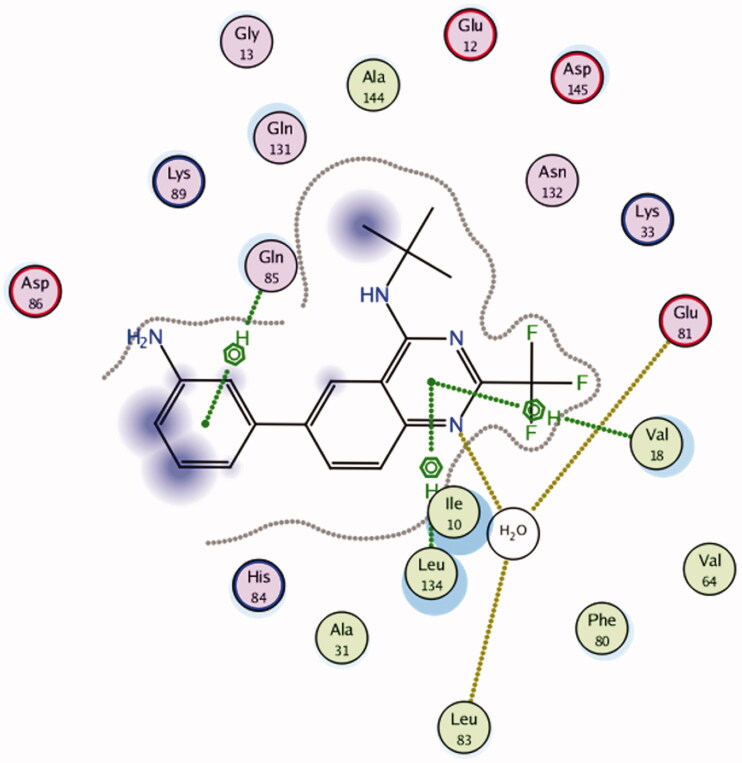
2D interaction diagram showing DIN-234325 interactions with the key amino acids in the CDK2 active site.

**Figure 5.**
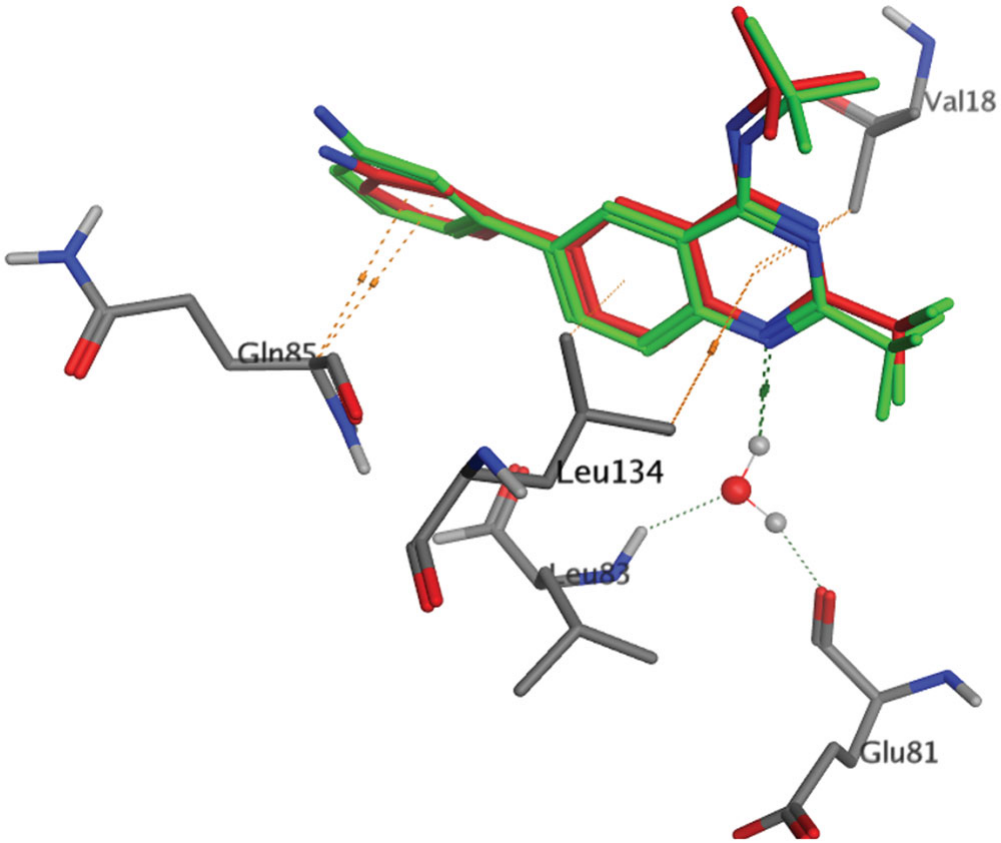
3D representation of the superimposition of the docked pose (red) and the co-crystallised quinazoline ligand (green) in the CDK2 binding site (RMSD = 0.485 Å).

To confirm the accuracy of the docking procedure, validation was performed by re-docking of the co-crystallised ligand into the catalytic domain of CDK2. The validation results revealed that the used docking protocol is suitable for the proposed docking study. This is demonstrated by the perfect alignment between the coordinates of DIN-234325 and its self-docked pose with root mean square deviation (RMSD) of 0.485 Å, docking score (S) of −18.24 Kcal mol^−1^. Additionally, the self-docked pose was able to achieve the same binding interactions depicted by the co-crystallised ligand with the main amino acids in the CDK2 binding site (Figures 4, 5).

**Figure 6.**
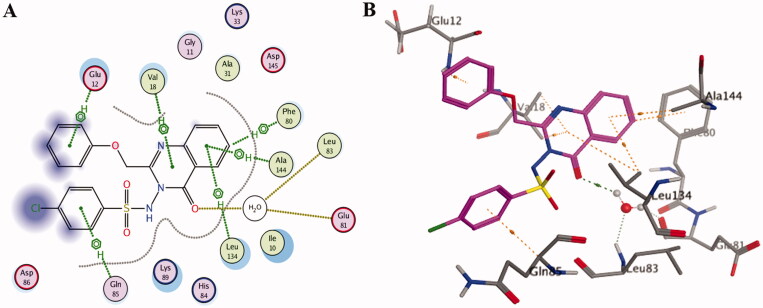
2D diagram (A) and 3D representation (B) of quinazolinone **5c** showing its interactions with the CDK2 binding site.

The outcomes of the molecular docking study showed the binding mode of compound **5c** within the binding pocket of CDK2 kinase (Figure 6; binding score (S) of −13.8 Kcal mol^−1^) indicating that two hydrogen bonds are formed between the quinazolinone moiety and the hinge region. Specifically, the quinazolinone carbonyl oxygen forms two water-mediated hydrogen bonds with the Leu83-NH and Glu81-CO. Moreover, the planar aromatic core makes extensive hydrophobic contacts with Ala31, Phe80, Ile10, Leu134, Val18, and Ala144 in a similar mode to that of the co-crystallised ligand. Additionally, other hydrogen-pi interactions were observed between the phenyl rings attached to the C2 and N2 of the quinazolinone ring and Glu12 and Gln85, respectively.

#### 3.3.2. Drug likeness properties

**Figure 7.**
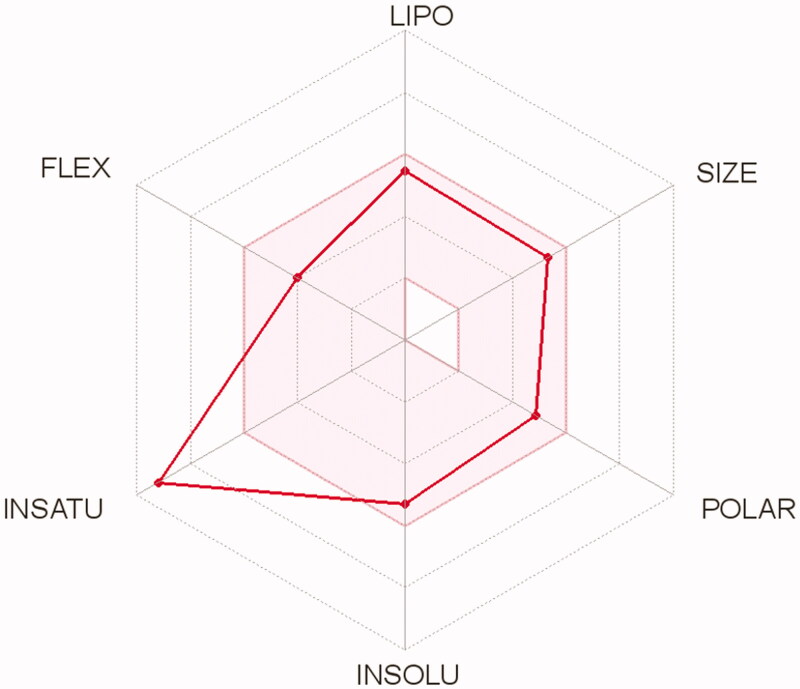
Bioavailability radar chart of compound **5c**. The pink area represents the range of the optimal property values for oral bioavailability and the red line is compound **5c** predicted properties; saturation (INSATU), size (SIZE), polarity (POLAR), solubility (INSOLU), lipophilicity (LIPO), and flexibility (FLEX).

The drug-likeness profile for the most potent candidate **5c** was predicted using SwissADME server^48^. The results of the Swiss ADME prediction of drug-likeness of this compound revealed no alerts for Pan Assay Interfering Substances (PAINS) and no violation to Lipinski and other pharmacokinetics filters (for more details see supporting materials, Figure S1). Moreover, the selected quinazolinone **5c** showed a predicted log Po/w value in the range of 3.25–4.06 with moderate water solubility, high GIT absorption, and no BBB permeability and thus minimal predicted CNS adverse effects. Furthermore, **5c** is not P-glycoprotein substrates (PGP−) so it is not liable to the efflux by this transporter and thus avoiding the drug-resistance mechanism used by some tumour cell lines^56^ (for boiled egg chart of compound **5c** see supporting materials, Figure S2). The oral bioavailability radar chart of compound **5c** demonstrated its good predicted oral bioavailability (Figure 7). Collectively the tested compound possesses promising physicochemical and pharmacokinetic properties.

## 4. Conclusion

In this study, three series of quinazolin-4(3*H*)-one derivatives were designed and synthesised as CDK2 inhibitors. All the newly synthesised compounds were screened for their antiproliferative activity against a panel of sixty cancer cell lines. The quinazolinones **5c** and **8a** displayed excellent growth inhibition against the melanoma cell line MDA-MB-435 (GI% = 94.53 and 94.15, respectively). The quinazolinone **5c** also exhibited promising activity against the glioblastoma cell line SNB-75 (GI% = 63.09), while **8a** showed potent to moderate activity against leukaemia cell lines SR (GI% = 69.55) and K-562 (GI% = 67.80). Consequently, the IC_50_ of **5c** and **8a** against the most sensitive cell line **(**MDA-MB-435) in this research work was detected. Compound **5c** has emerged as the most active member against MDA-MB-435 with IC_50_ value of 3.03 µM which is 1.7-folds more potent than roscovitine, the reference standard (IC_50_ = 5.26 µM). Compound **8a** also showed good potency on MDA-MB-435 cell line with IC_50_ value of 6.22 µM which is comparable to that of the reference drug. The quinazolinone **5c** triggered cell cycle arrest in the S phase in melanoma MDA-MB-435 cells and in the G2/M phase in glioblastoma SNB-75 cells suggesting that CDK2 could be the potential biological target for the newly synthesised congeners. Furthermore, compound **5c** induced apoptosis in MDA-MB-435 and SNB-75 cell lines as indicated by the percent of the total apoptosis of 28.65 and 44.22%, respectively in comparison to the positive control, annexin V (% apoptosis 1.91, 2.53). Accordingly, the most active compounds **5c** and **8a** were tested for their CDK2 inhibitory activities. Interestingly, the outcomes of CDK2 inhibitory assay were consistent with that of the cytotoxicity data. The quinazolinones **5c** and **8a** displayed CDK2 IC_50_ values of 0.63 µM and 1.74 µM, sequentially in comparison with the used reference roscovitine (IC_50_ = 1.28 µM). The molecular docking study confirmed the biological results *via* prediction of the possible binding mode of the most active compound **5c** with CDK2 active site indicating its ability to form a pair of crucial hydrogen bonds with the CDK2 hinge region (Leu83 and Glu81). Moreover, the most active compound **5c** possessed promising pharmacokinetic properties as depicted by the computational ADME study. These outcomes endorsed that the most active candidates **5c** and **8a** may serve as useful lead compounds in the search for promising anti-melanoma agents acting through CDK2 inhibition.

## Supplementary Material

Supplemental Material
