# Supplementary material for: Development of newly synthesised quinazolinone-based CDK2 inhibitors with potent efficacy against melanoma
Source: J Enzyme Inhib Med Chem. 2022 Feb 9;37(1):686–700. doi: 10.1080/14756366.2022.2036985 (PMC8843100; doi:10.1080/14756366.2022.2036985)
Supplement: Supplemental Material [file IENZ_A_2036985_SM4411.pdf]

Supplementary Material

Development of newly synthesized quinazolinone-based CDK2 inhibitors with potent efficacy against melanoma

Eman R. Mohammed and Ghada F. Elmasry

Department of Pharmaceutical Chemistry, Faculty of Pharmacy, Cairo University, El-Kasr El-Eini Street, P.O. Box 11562 Cairo, Egypt

Table of contents

Item	Page no.
1. Cytotoxicity screening against a panel of 60 human tumor cell lines	3
2. Detection of IC_{50} against MDA-MB-435, SNB-75 and WI-38 cell lines	3
3. Cell cycle analysis	4
4. Apoptosis determination	4
5. CDK2 inhibition assay	5
6. Drug likeness figures	5-6
7. Microanalysis data	7-9
8. IR spectra	10-21
9. NMR spectra	22-36
10. Mass spectra	37-48

1. Cytotoxicity screening against a panel of 60 human tumor cell lines

All the compounds were selected for NCI-60 Human Tumor Cell Lines Screen by the Developmental Therapeutics Program (DTP) at the National Cancer Institute (NCI), Maryland, USA. The operation of this screen utilizes 60 different human tumor cell lines, representing leukemia, non-small-cell lung cancer, colon cancer, CNS cancer, melanoma, ovarian cancer, renal cancer, prostate cancer and breast cancer.

The compounds were supplied as dry powder and a single concentration is tested in all 60 cell lines at a single dose of 10 µM solution in dimethyl sulfoxide (DMSO) using Sulforhodamine B assay. Briefly, cells are seeded in 96 well plates at an appropriate density and incubated for 1 day. After 1 day, some of the plates are processed to determine the density time zero. To the remaining plates, compounds are added at 10 µM concentration. Plates are incubated a further 2 days, then fixed and stained with sulphorhodamine B. Growth inhibition is calculated relative to cells without drug treatment and the time zero control. The use of a time zero control allows the determination of cell kill as well as net growth inhibition.

2. Detection of IC_{50} against MDA-MB-435, SNB-75 cell lines and normal cell line WI-38.

Antiproliferative activities of **5c** in melanoma cell line MDA-MB-435, glioblastoma cell line SNB-75 and normal cell line WI-38 and that of **8a** in melanoma cell line MDA-MB-435 and normal cell line WI-38 were measured spectrophotometrically using in vitro MTT based toxicology assay kit (catalog no. M-5655, M-8910) (Sigma Aldrich), according to the manufacturer's protocol. The three examined cell lines were obtained from American Type Culture Collection (ATCC). Briefly, cells were platted (cells density 1.2 – 1.8 × 10,000 cells/well) in a volume of 100 µl complete growth medium + 100 µl of the tested compound per well in a 96-well plate for 48 h before the MTT assay. The cultures were removed from incubator into laminar flow hood then each vial of MTT [M-5655] should be reconstituted with 3 ml of medium or balanced salt solution without phenol red and serum. Reconstituted MTT was added in an amount equal to

10% of the culture medium volume. Cultures were returned to incubator for 2–4 h. After the incubation period, cultures were removed from incubator and the resulting formazan crystals were dissolved by adding an amount of MTT solubilization solution [M-8910] equal to the original culture medium volume. Dissolution is enhanced by gentle mixing in a gyratory shaker. Then the absorbance is measured spectrophotometrically at a wavelength of 450 nm. The absorbance of multiwell plates was measured at 690 nm and subtracted from the 450 nm measurement. IC_{50} values (concentration of sample causing 50% loss of cell proliferation of the vehicle control) were calculated using non-linear regression curve fitting of the dose response plots on GraphPad Prism 7.0 software. Data presented are the results of at least three independent experiments. The results of these studies are presented as mean IC_{50} (µM) ± standard deviation (SD).

3. Cell cycle analysis

Melanoma MDA-MB-435 and glioblastoma SNB-75 cells were treated with the quinazolinone **5c** for 24 h (at its IC_{50} concentration), and then cells were washed twice with ice-cold phosphate buffered saline (PBS). Consequently, the treated cells were collected by centrifugation, fixed in ice-cold 70% (v/v) ethanol, washed with PBS, re-suspended with 100 µg/mL RNase, stained with 40 µg/mL propidium iodide (PI), and analyzed by flow cytometry using FACS Calibur (Becton Dickinson, BD, Franklin Lakes, NJ, USA). The cell cycle distributions were calculated using Cell Quest software 5.1 (Becton Dickinson).

4. Apoptosis determination

Annexin V fluorescein isothiocyanate (FITC)/PI apoptosis detection kit (BD Biosciences) was used according to the manufacturer's instructions to measure the apoptotic activity of compound **5c**. Melanoma MDA-MB-435 and glioblastoma SNB-75 cells were cultured to a monolayer then treated with the quinazolinone **5c** at its IC_{50} concentration. The cells then were collected by trypsinisation and 0.5×10^6 cells were washed twice with PBS and stained with 5 µL Annexin V-FITC and 5 µL PI in 1Xbinding buffer for 15 min. at room temperature in the dark. Analyses were performed using FACS Calibur flow cytometer (BD Biosciences, San Jose, CA).

5. CDK2 inhibition assay

CDK2 enzyme inhibition activity was measured for the quinazolinones **5c** and **8a** using a colorimetric 96-well CDK2 assay kit (BPS Bioscience) (catalog no. 79599), according to the manufacturer's protocol. All the IC_{50} values were calculated using GraphPad Prism 7.0 Software.

6. Drug likeness figures

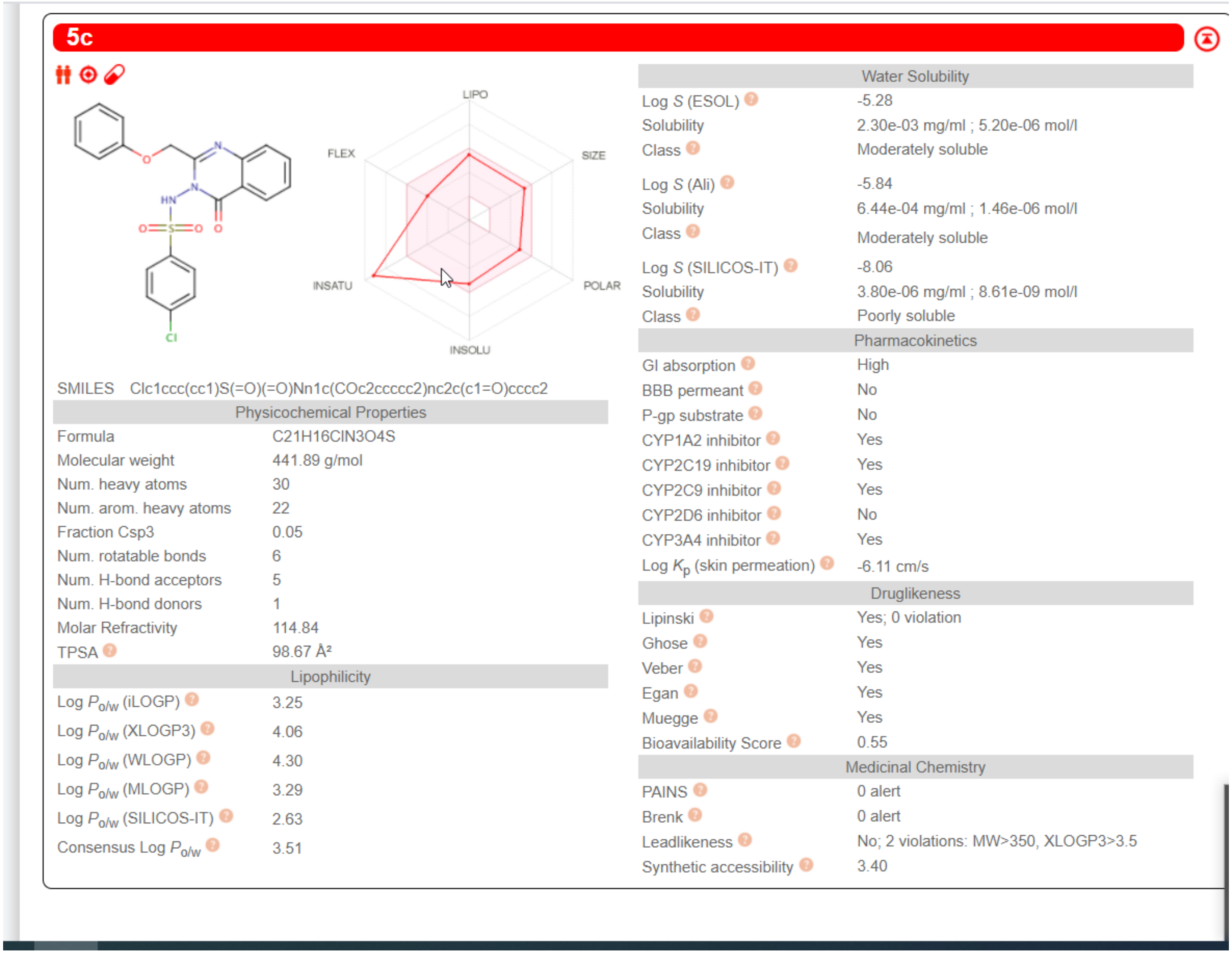

Figure S1. The pharmacokinetic profile of compound **5c** using SwissADME web server.

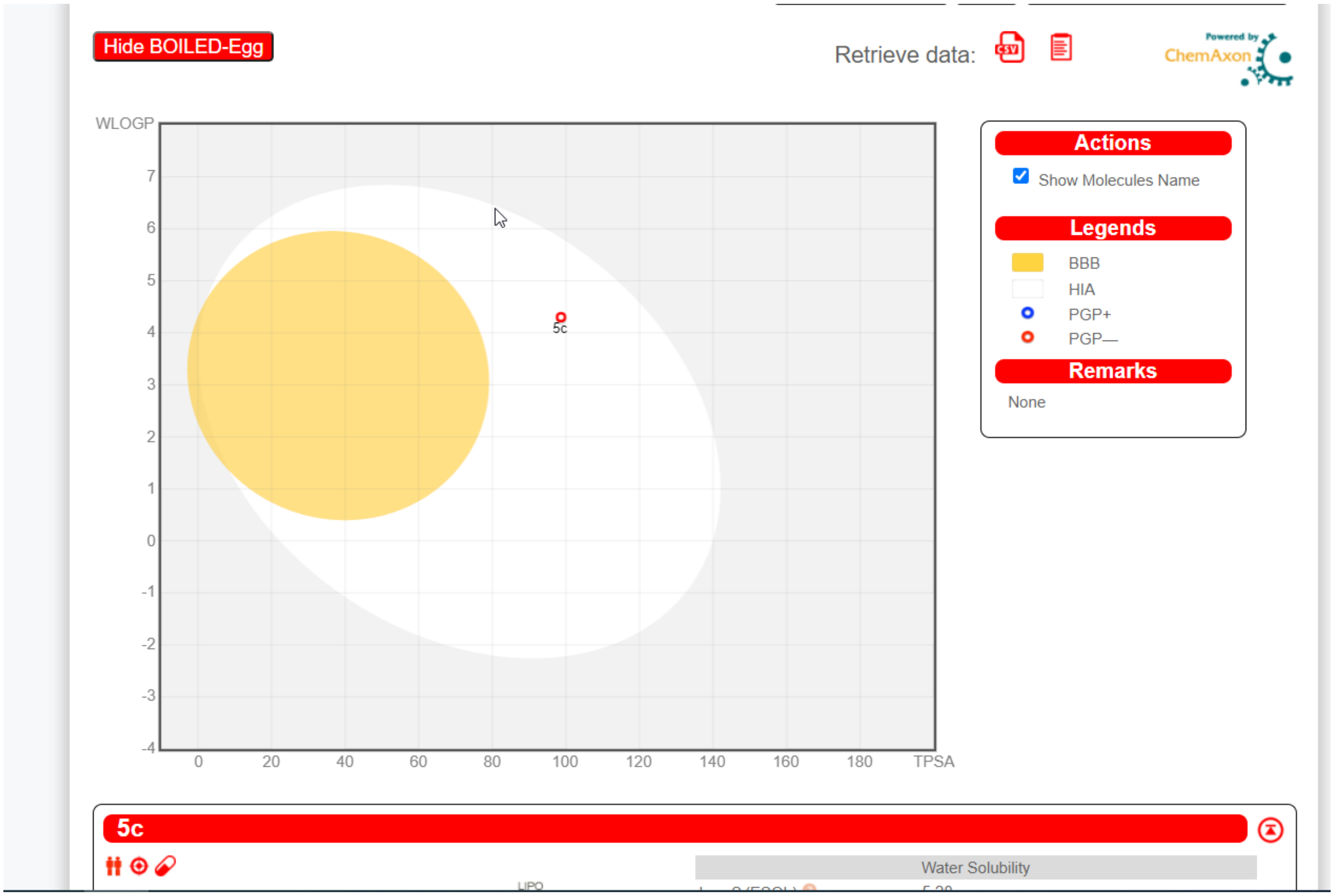

Figure S2. Boiled-egg chart of compound **5c**.

7. Microanalysis Data

جامعة الازهر
Al-Azhar University
المركز الإقليمى للفطريات وتطبيقاتها
The Regional Center for Mycology and Biotechnology

Requester Data:

Name: Dr. Eman Raafat
Authority: Faculty of Pharmacy, Cairo University

Sample Data:

Eight samples had been submitted for elemental analysis.

Analysis Report:

Sample Code	C%	H%	N%
BCH3 5b	62.59	4.62	10.15
BCl 5c	57.24	3.71	9.72
Bun 5a	61.74	4.34	10.52
Cin 6b	75.38	5.14	11.25
ROCH3 4b	67.13	5.41	13.30
RCl 4c	63.29	4.62	13.14
Run 4a	69.16	5.21	14.23
Van 6a	68.65	4.89	10.64

INVESTIGATOR M. Yasser

DIRECTOR

شارع المخيم الدائم – مدينة نصر – القاهرة
البريد الإلكترونى : rcmb@azhar.edu.eg
الموقع الإلكترونى:
صندوق بريد ١١٧٥١ مدينة نصر القاهرة
تليفون: ٢٢٦٢٠٣٧٣ (٠٢٠٢)
فاكس: ٢٢٦٢٠٣٧٣ (٠٢٠٢)
http:// www.azhar.edu.eg.htm
http://www.azhar.edu.eg/pages/fungi_center.htm
Facebook: RCMB AZHAR

CS Scanned with CamScanner

Requester Data:

Name: Dr. Eman Raafat
Authority: Faculty of Pharmacy, Cairo University

Sample Data:

Three samples had been submitted for elemental analysis.

Analysis Report:

Sample Code	C%	H%	N%
8c Methoxy	67.45	6.09	14.28
8a Methyl	64.71	6.27	17.43
8b Ph	68.95	5.97	15.11

INVESTIGATOR **DIRECTOR**

شارع المخيم الدائم - مدينة نصر - القاهرة
البريد الإلكتروني: rcmb@azhar.edu.eg
الموقع الإلكتروني:
صندوق بريد ١١٧٥١ مدينة نصر القاهرة
تليفون: ٢٢٦٢٠٣٧٣ (٠٢٠٢) فاكس: ٢٢٦٢٠٣٧٣ (٠٢٠٢)
http:// www.azhar.edu.eg.htm
http://www.azhar.edu.eg/pages/fungi_center.htm
Facebook: RCMB AZHAR

Scanned with CamScanner

8. IR Spectra

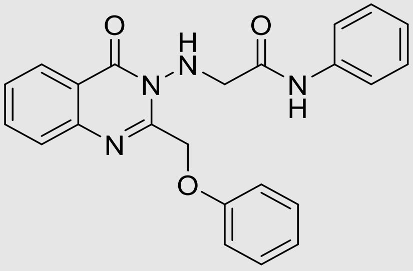

4a

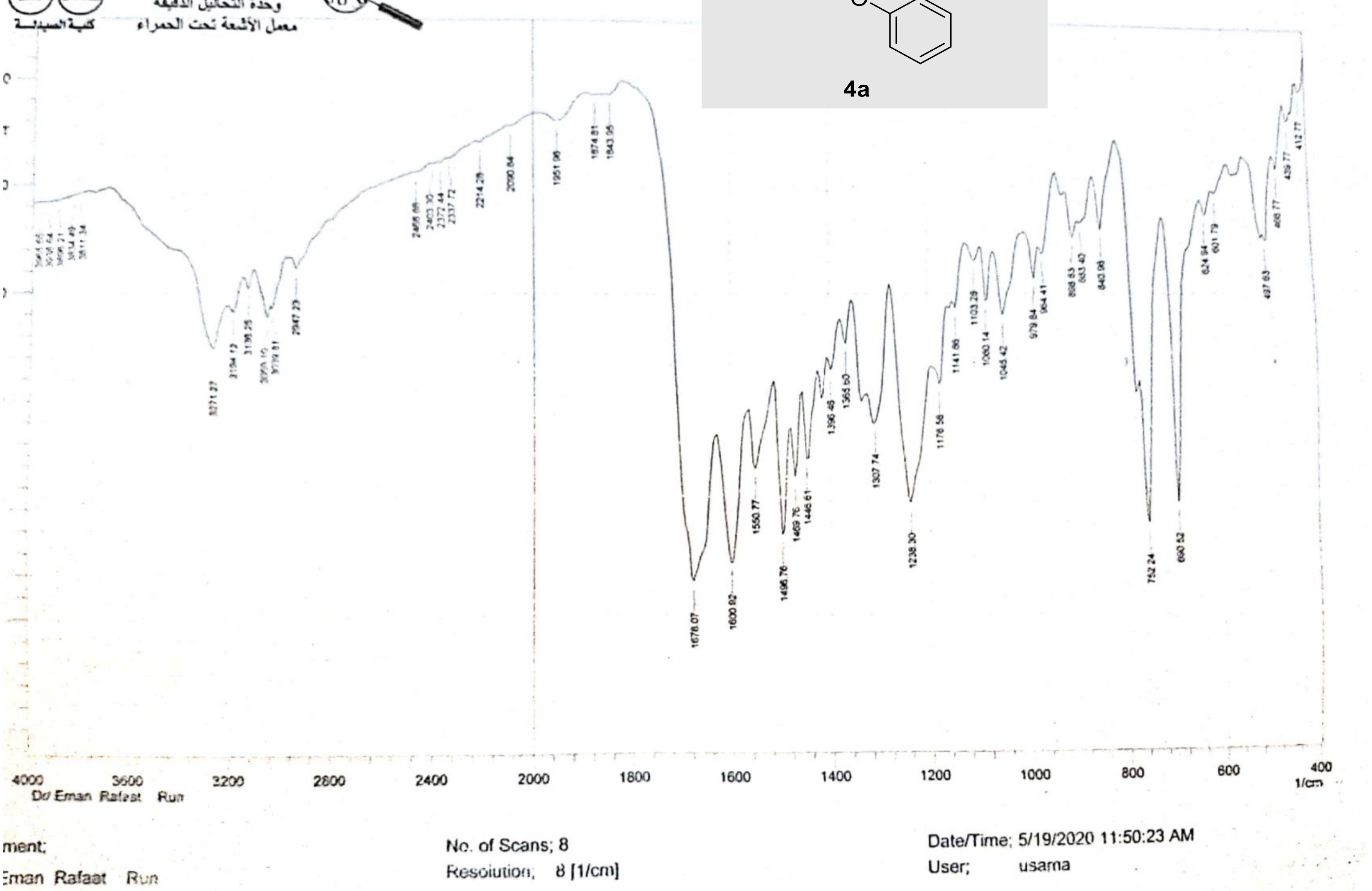

Scanned with CamScanner

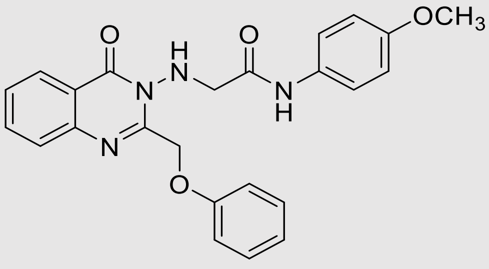
OCH3
O
N
H

4b

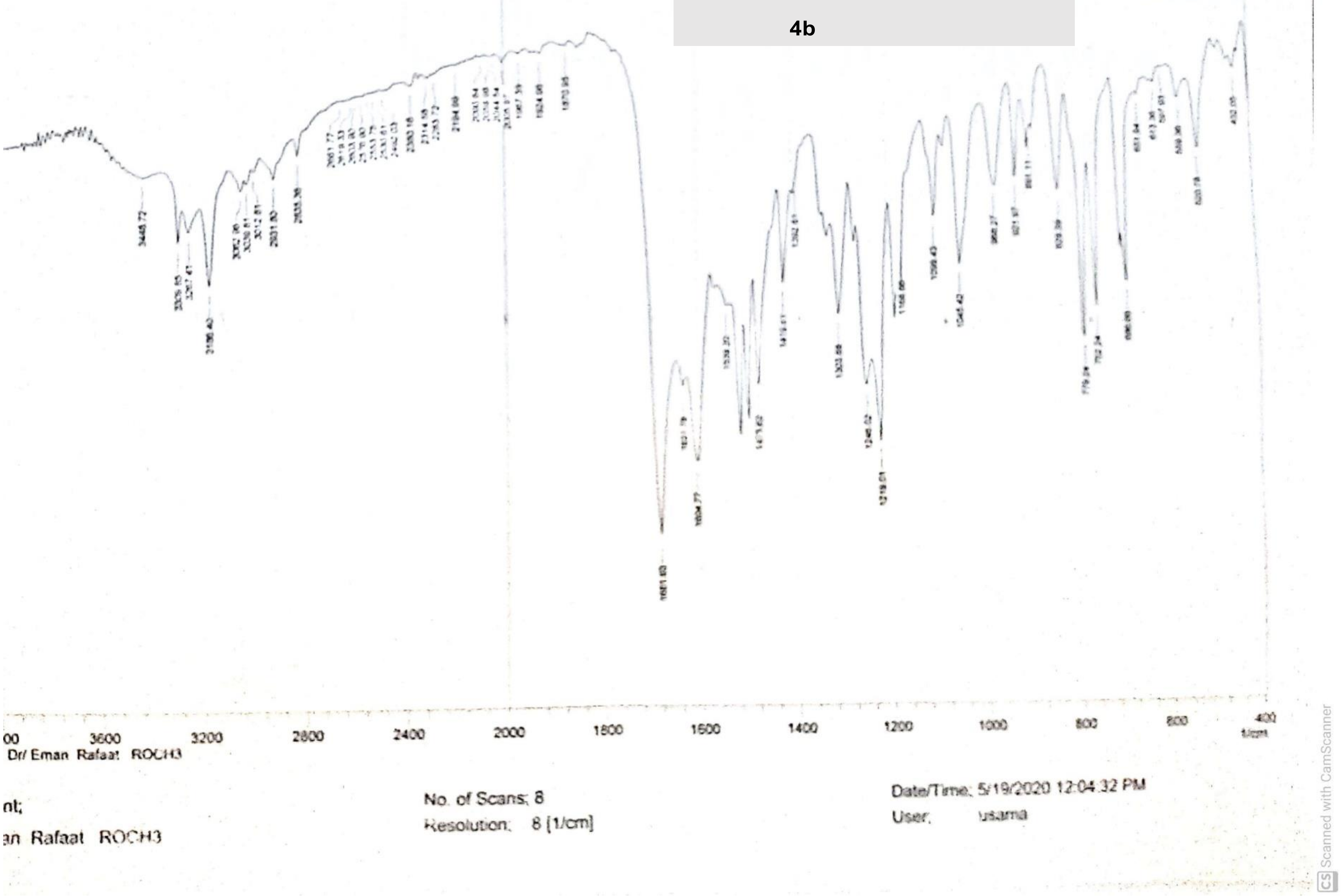
Microanalytical Unit-FOPCU
وحدة التحاليل الدقيقة
معمل الأشعة تحت الحمراء
Lab
SHIMADZU
00 3600 3200 2800 2400 2000 1800 1600 1400 1200 1000 800 600 400
1/cm
Dr/ Eman Rafaat ROCH3
No. of Scans; 8
Resolution; 8 [1/cm]
Date/Time; 5/19/2020 12:04:32 PM
User; usama
nt;
an Rafaat ROCH3

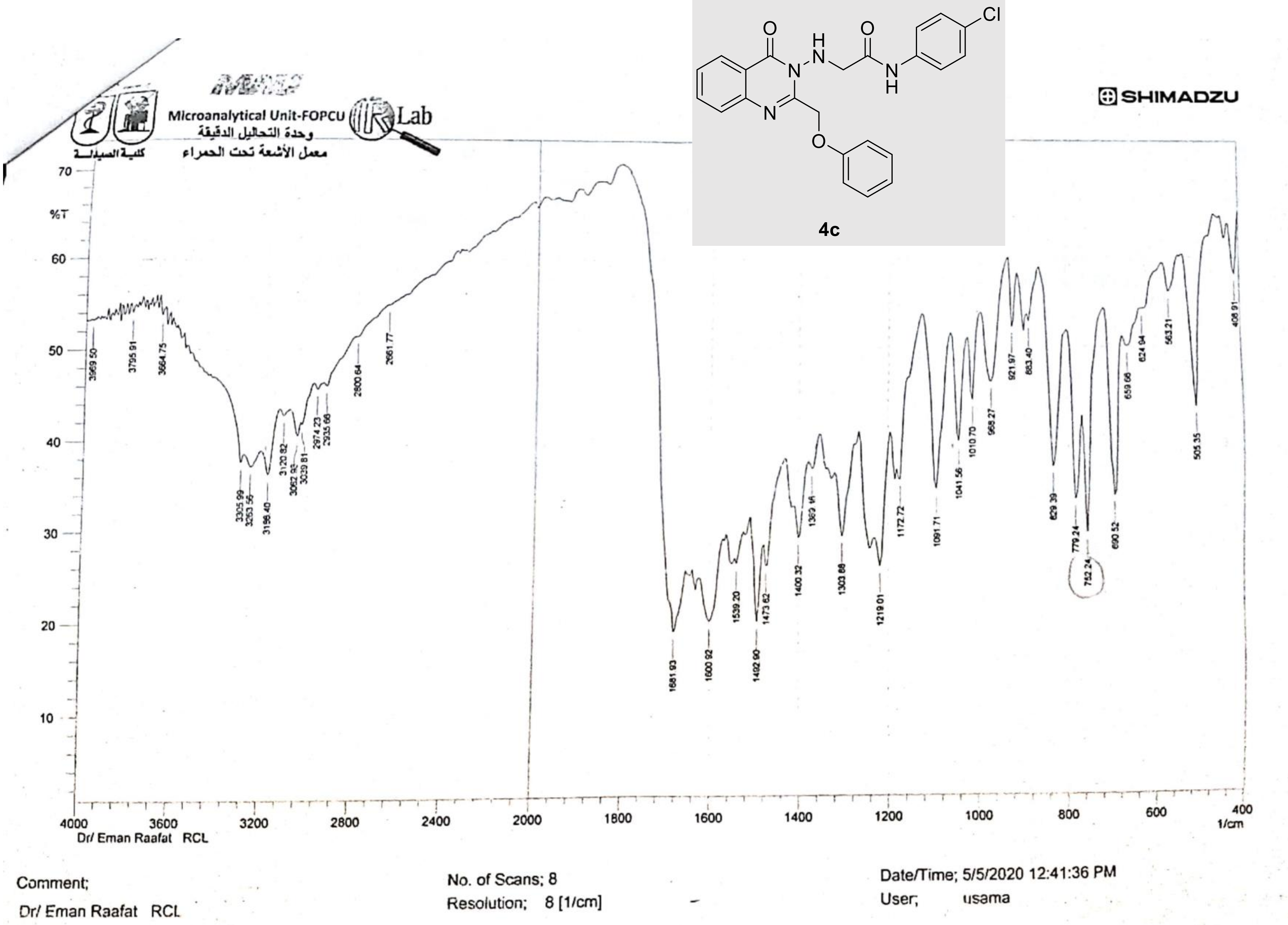

Scanned with CamScanner

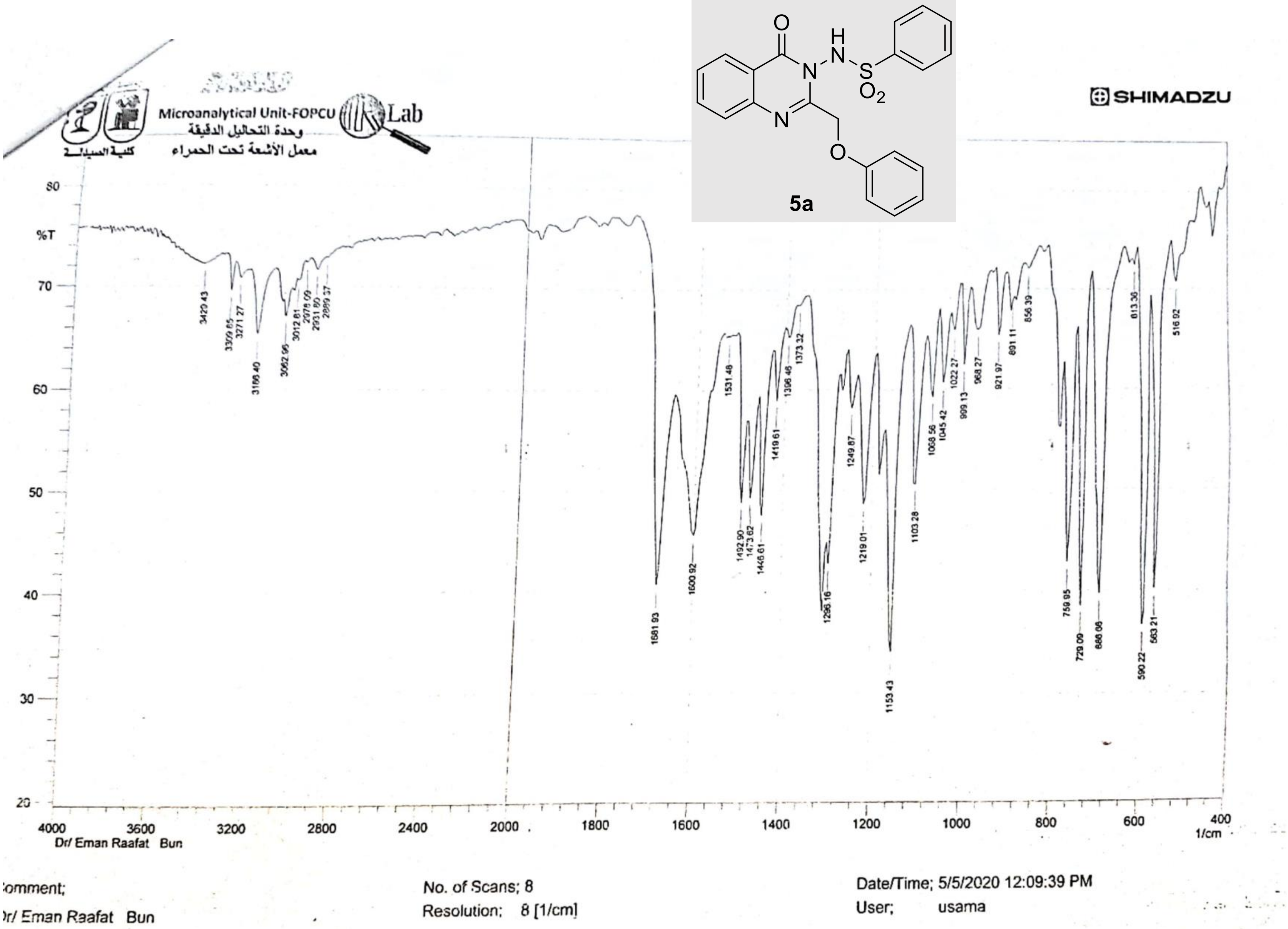

CS Scanned with CamScanner

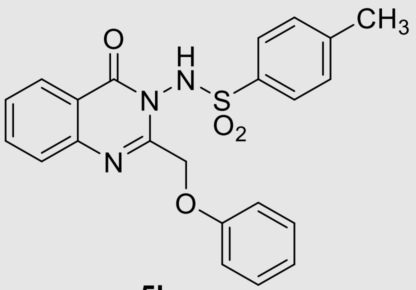

O
H
N
N
S
O_2
CH_3
N
O

5b

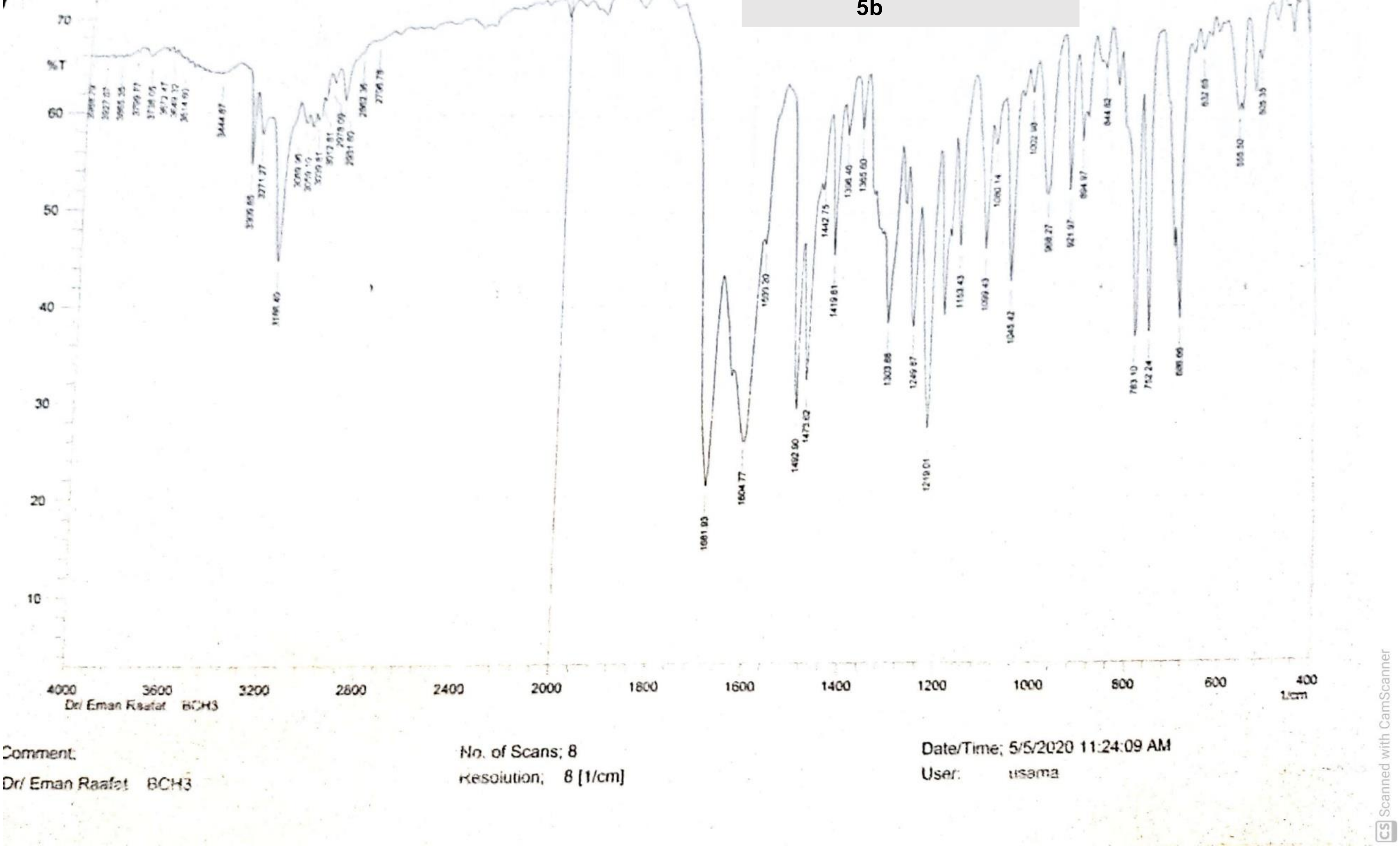

Microanalytical Unit-FOPCU
وحدة التحليل الدقيقة
معمل الأشعة تحت الحمراء
كلية الصيدلة
Lab
SHIMADZU
%T
70
60
50
40
30
20
10
4000
3600
3200
2800
2400
2000
1800
1600
1400
1200
1000
800
600
400
1/cm
3444.87
3271.77
3166.40
1681.93
1604.77
1531.20
1492.90
1473.62
1442.75
1419.61
1303.88
1249.87
1219.01
1153.43
1099.43
1045.42
908.27
921.97
763.10
752.24
686.66
Dr/ Eman Raafat BCH3
Comment;
Dr/ Eman Raafat BCH3
No. of Scans; 8
Resolution; 8 [1/cm]
Date/Time; 5/5/2020 11:24:09 AM
User: usama
Scanned with CamScanner

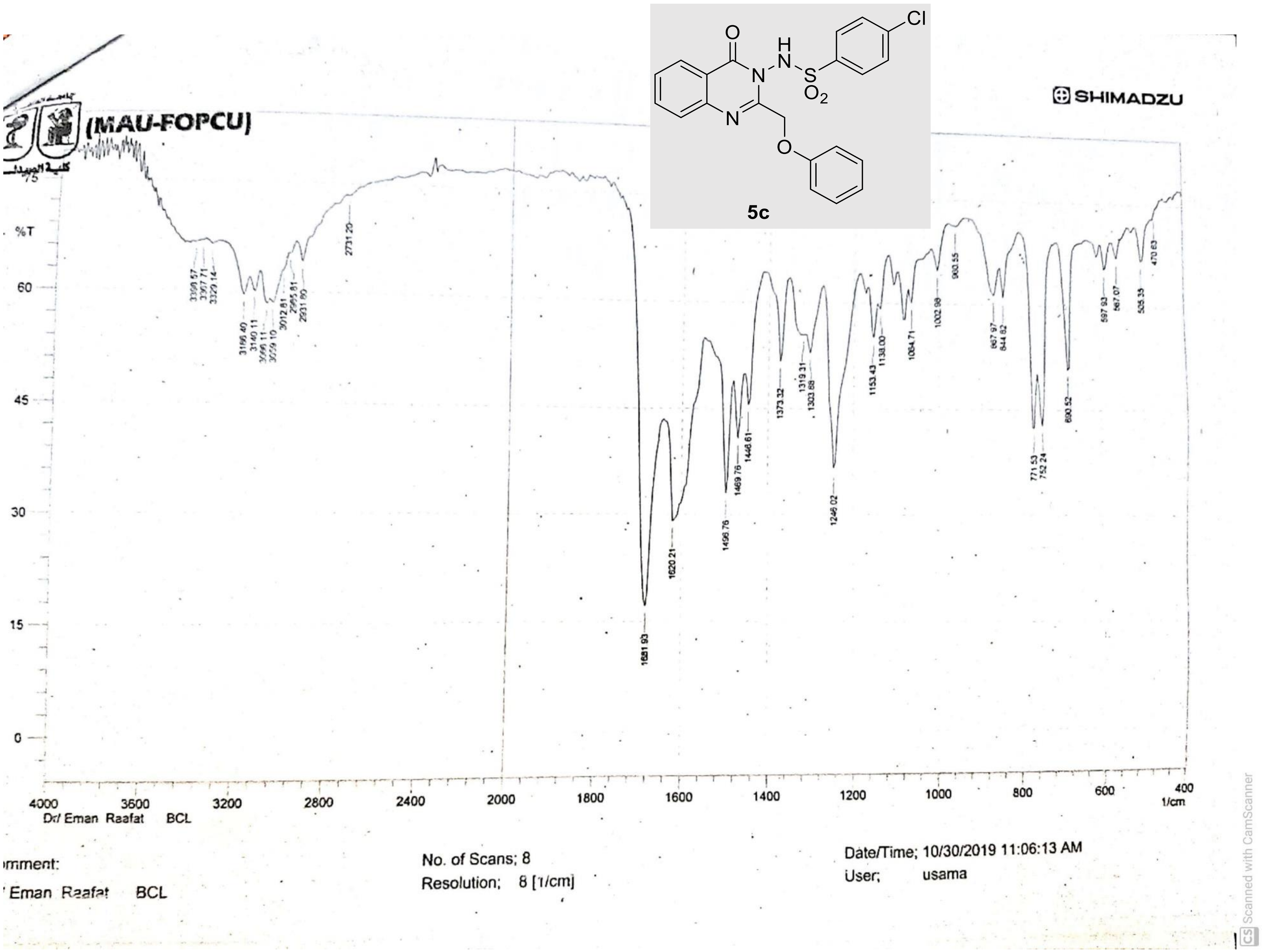

Scanned with CamScanner

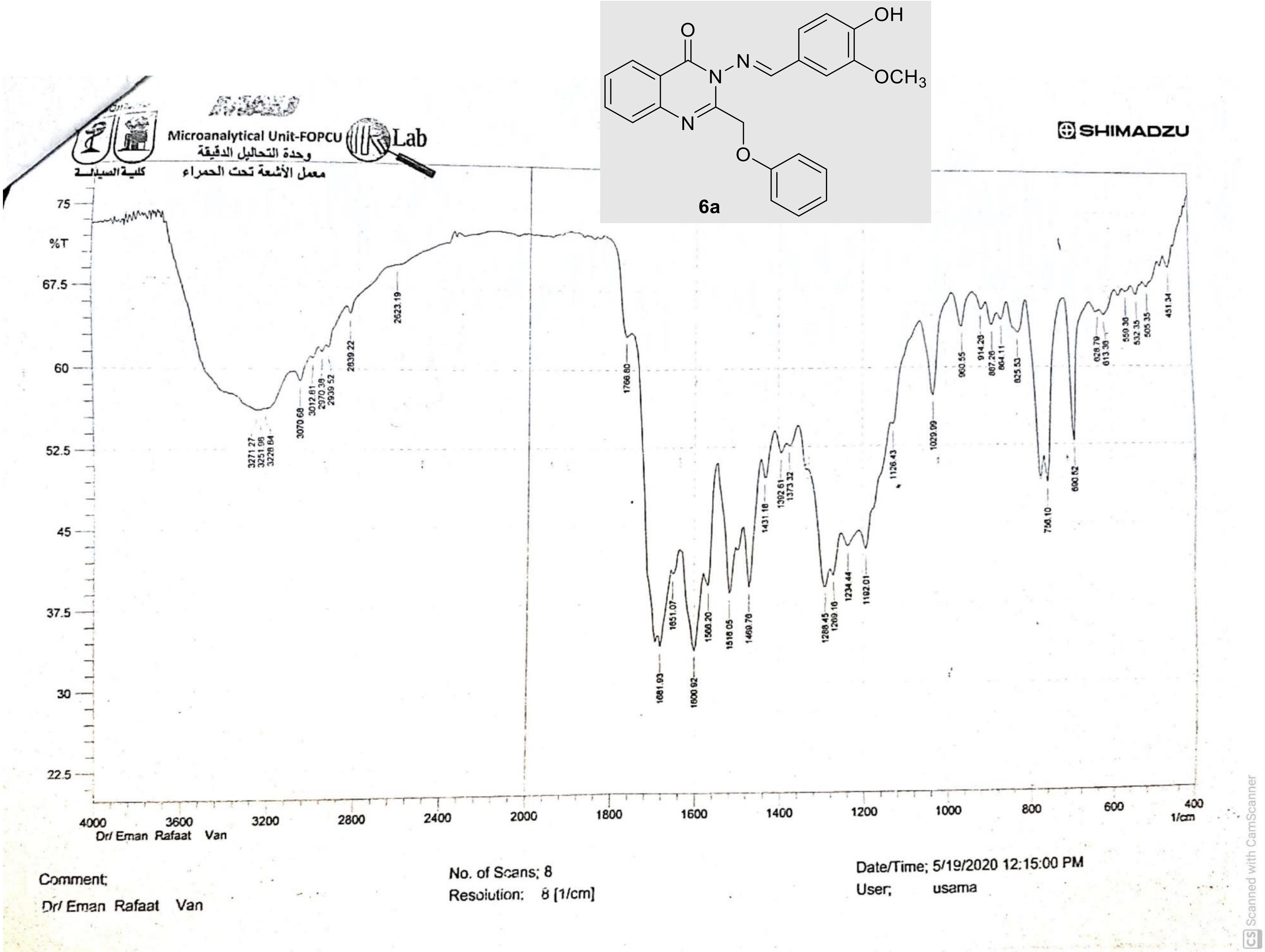

CS Scanned with CamScanner

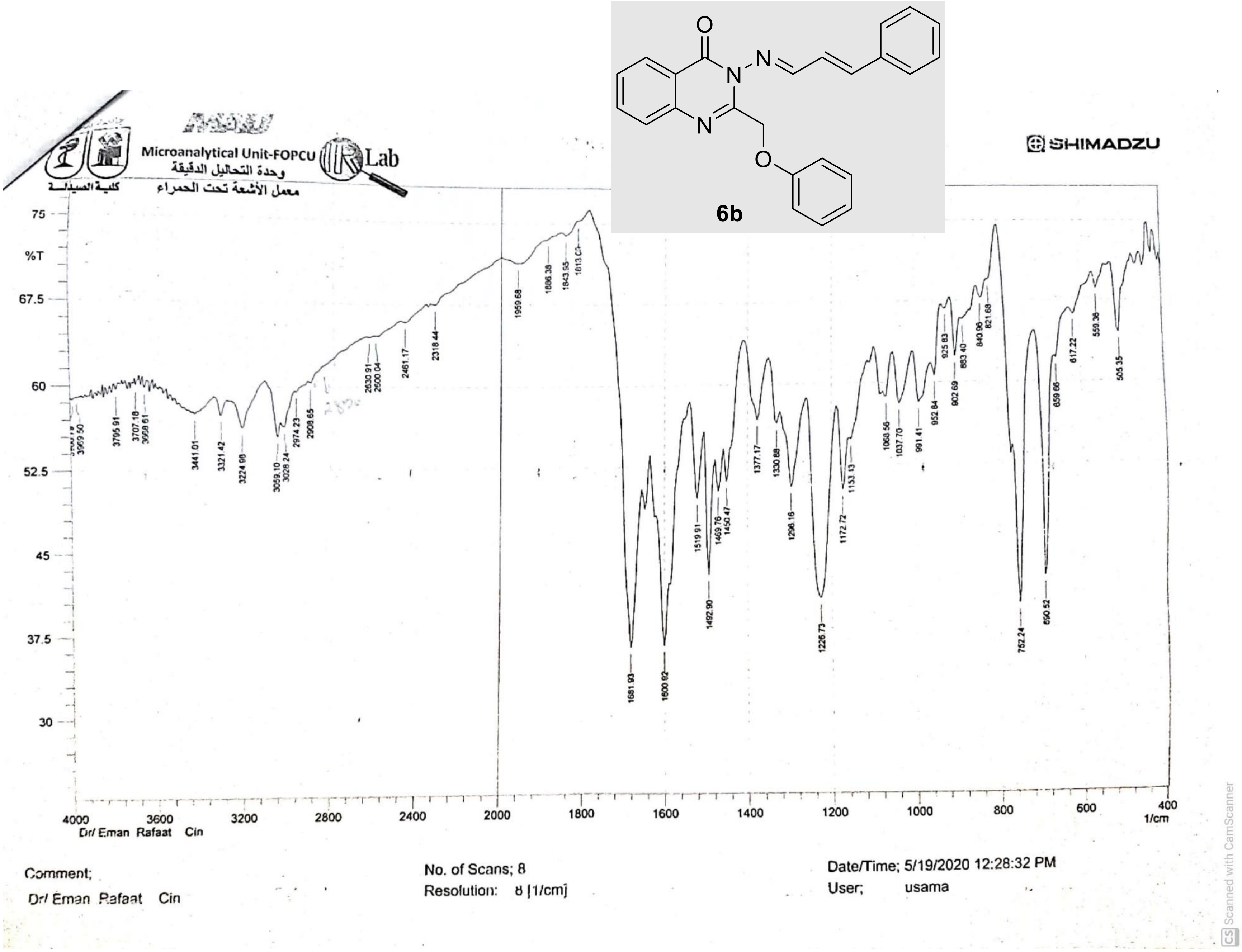

6b
Microanalytical Unit-FOPCU
وحدة التحاليل الدقيقة
معمل الأشعة تحت الحمراء
كلية الصيدلة
IR Lab
SHIMADZU
%T
75
67.5
60
52.5
45
37.5
30
4000
3600
3200
2800
2400
2000
1800
1600
1400
1200
1000
800
600
400
1/cm
Dr/ Eman Rafaat Cin
3795.91
3707.18
3658.61
3441.01
3321.42
3224.98
3059.10
3028.24
2974.23
2908.65
2630.91
2600.04
2461.17
2318.44
1959.68
1886.38
1843.95
1681.93
1600.92
1519.91
1492.90
1469.76
1450.47
1377.17
1330.88
1296.16
1226.73
1172.72
1153.13
1068.56
1037.70
991.41
952.84
925.83
902.69
883.40
840.96
821.68
752.24
690.52
659.66
617.22
559.36
505.35
Comment;
Dr/ Eman Rafaat Cin
No. of Scans; 8
Resolution: 8 [1/cm]
Date/Time; 5/19/2020 12:28:32 PM
User; usama
Scanned with CamScanner

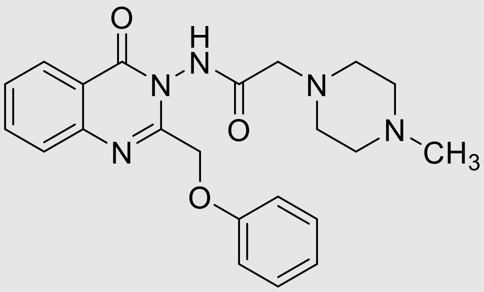

8a

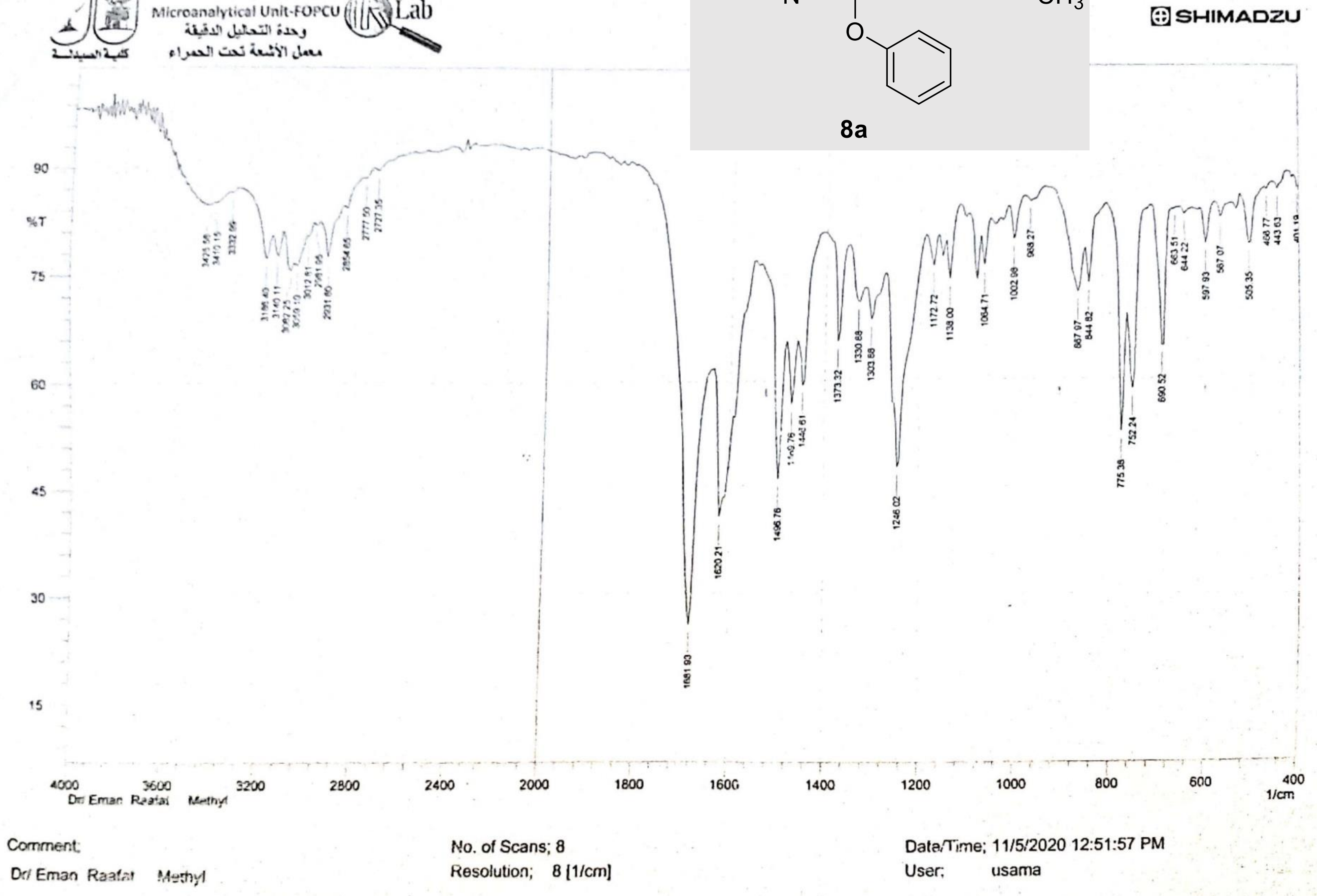

Scanned with CamScanner

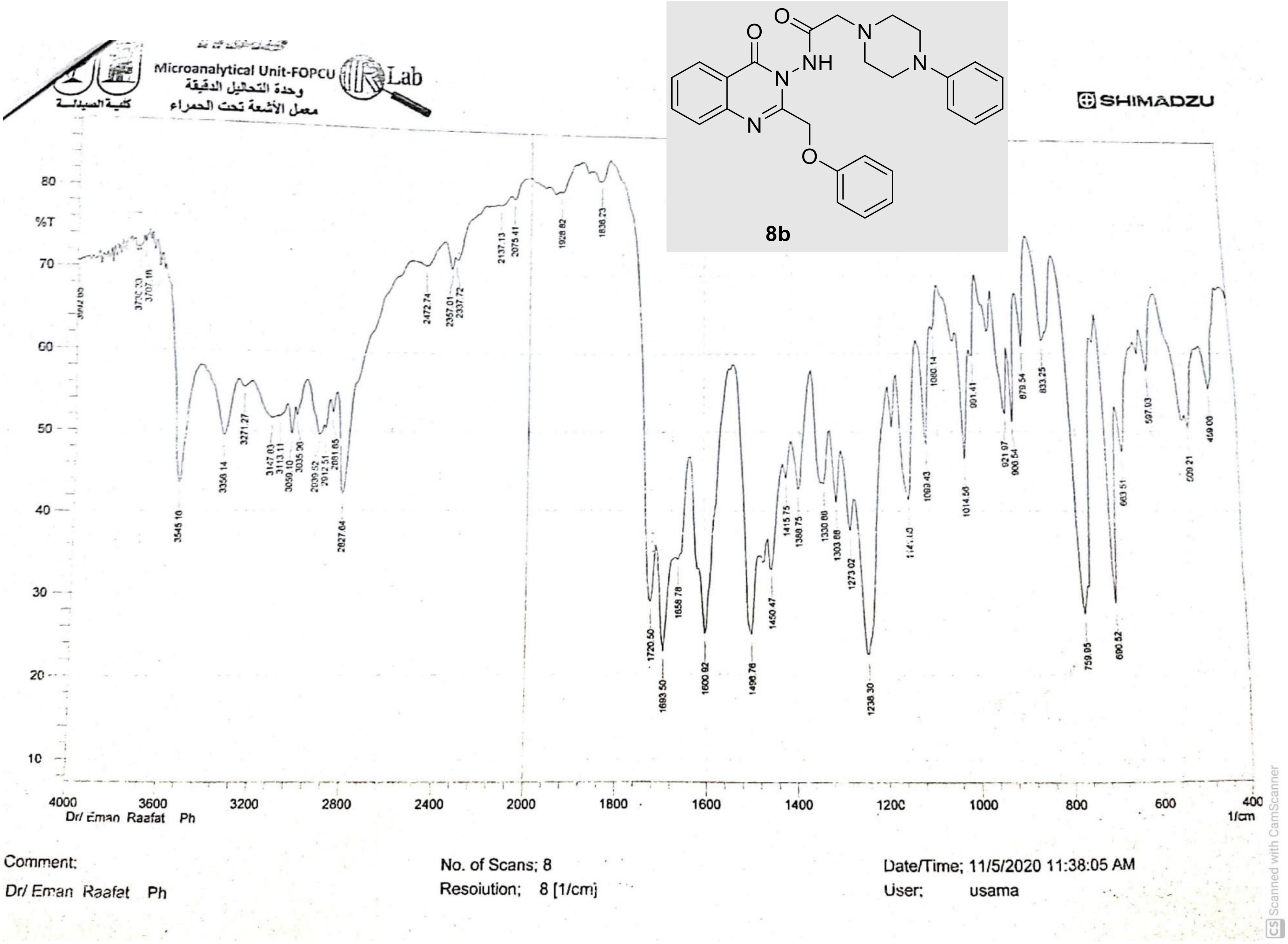

8b
Microanalytical Unit-FOPCU
وحدة التحاليل الدقيقة
معمل الأشعة تحت الحمراء
كلية الصيدلة
IR Lab
SHIMADZU
%T
1/cm
Dr/ Eman Raafat Ph
3545.10
3356.14
3271.27
2827.04
2472.74
2357.01
2337.72
2137.13
2075.41
1928.82
1836.23
1720.50
1693.50
1658.78
1600.92
1496.78
1450.47
1415.75
1388.75
1330.88
1303.88
1273.02
1238.30
1080.14
1014.56
991.41
921.97
900.54
879.54
833.25
759.95
690.52
663.51
597.93
509.21
459.00
Comment;
Dr/ Eman Raafat Ph
No. of Scans; 8
Resoiution; 8 [1/cm]
Date/Time; 11/5/2020 11:38:05 AM
User; usama

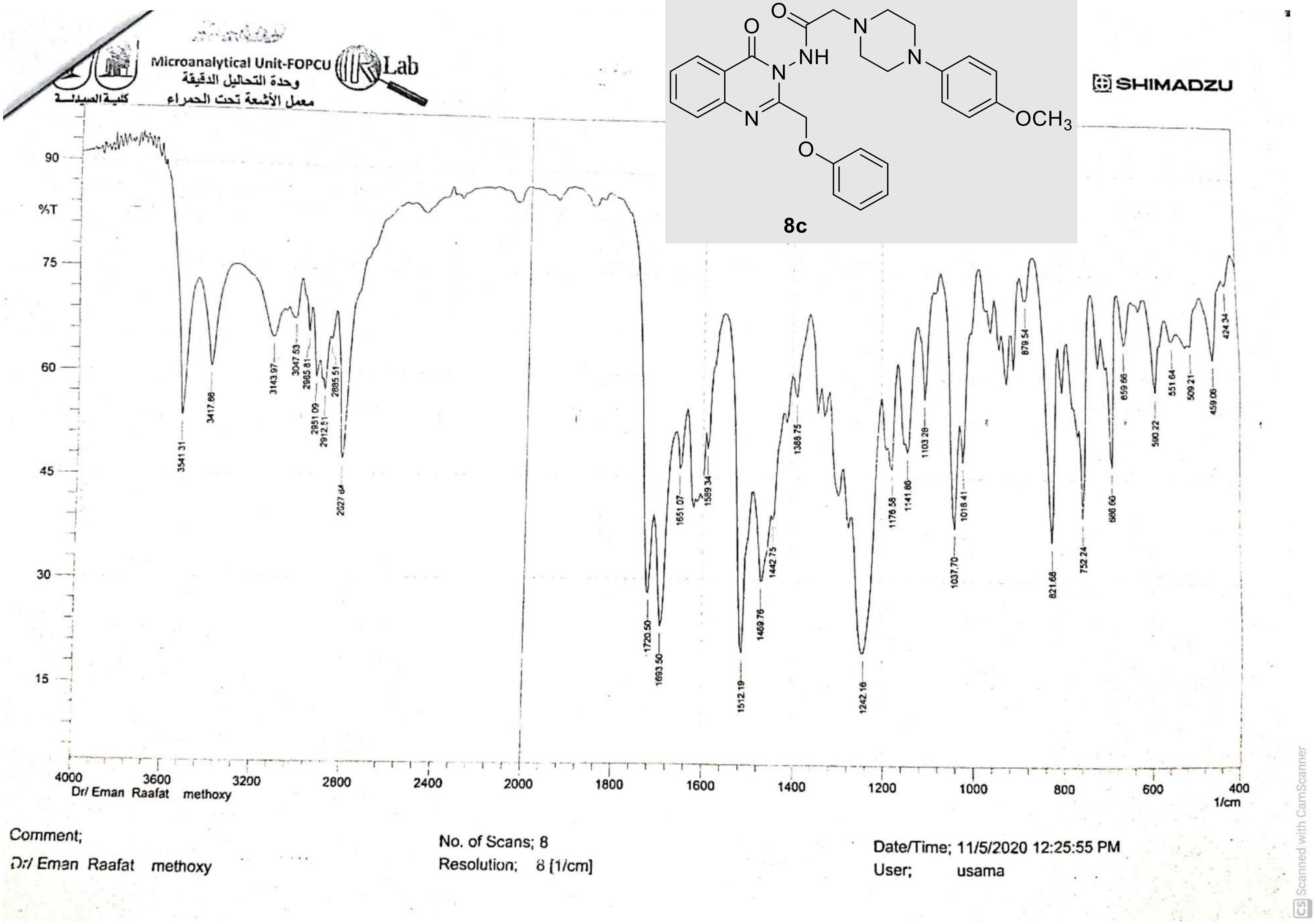

8c

CS Scanned with CamScanner

9. NMR Spectra

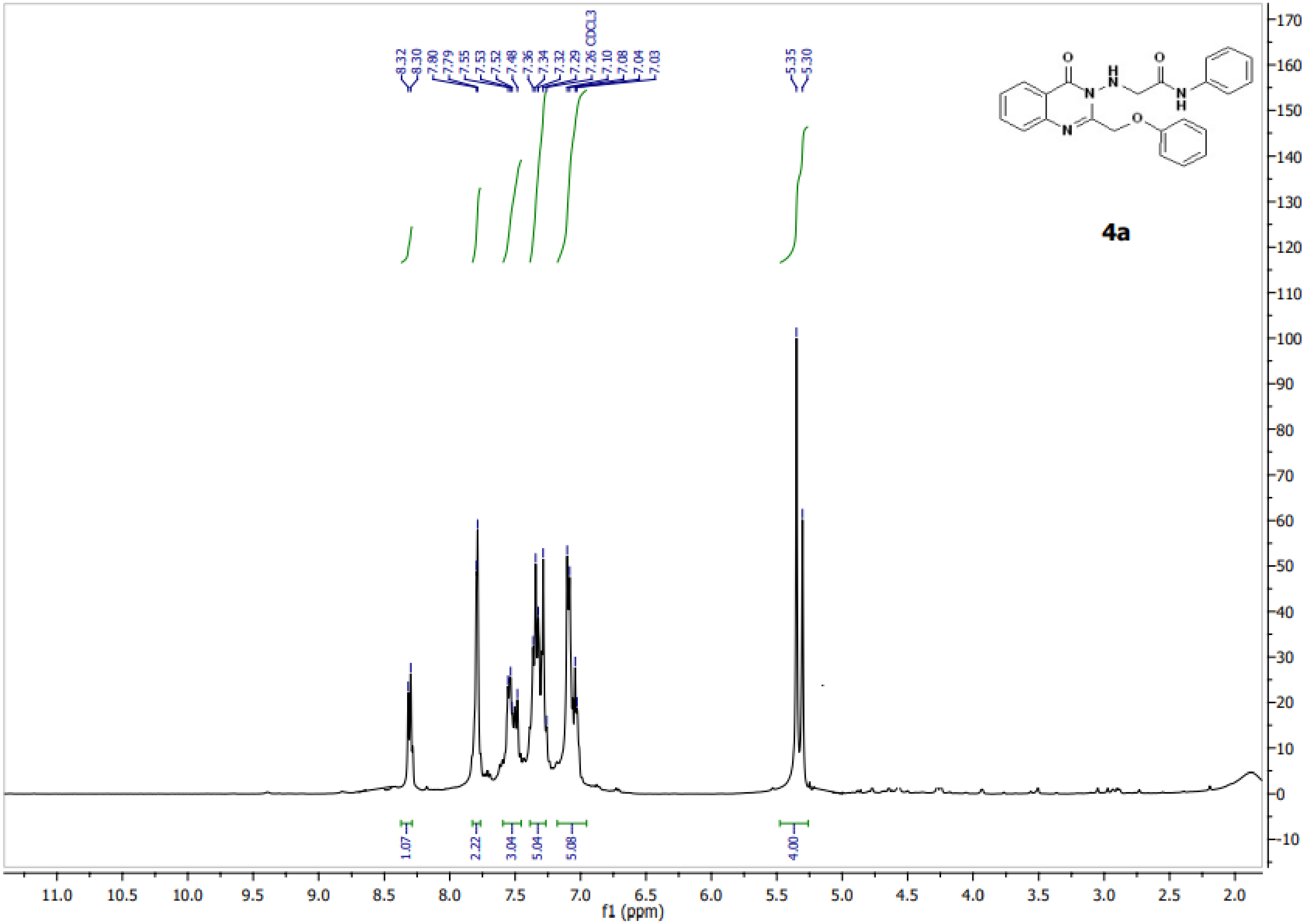
8.32
8.30
7.80
7.79
7.55
7.53
7.52
7.48
7.36
7.34
7.32
7.29
7.26 CDCl3
7.10
7.08
7.04
7.03
5.35
5.30
1.07
2.22
3.04
5.04
5.08
4.00
f1 (ppm)

4a

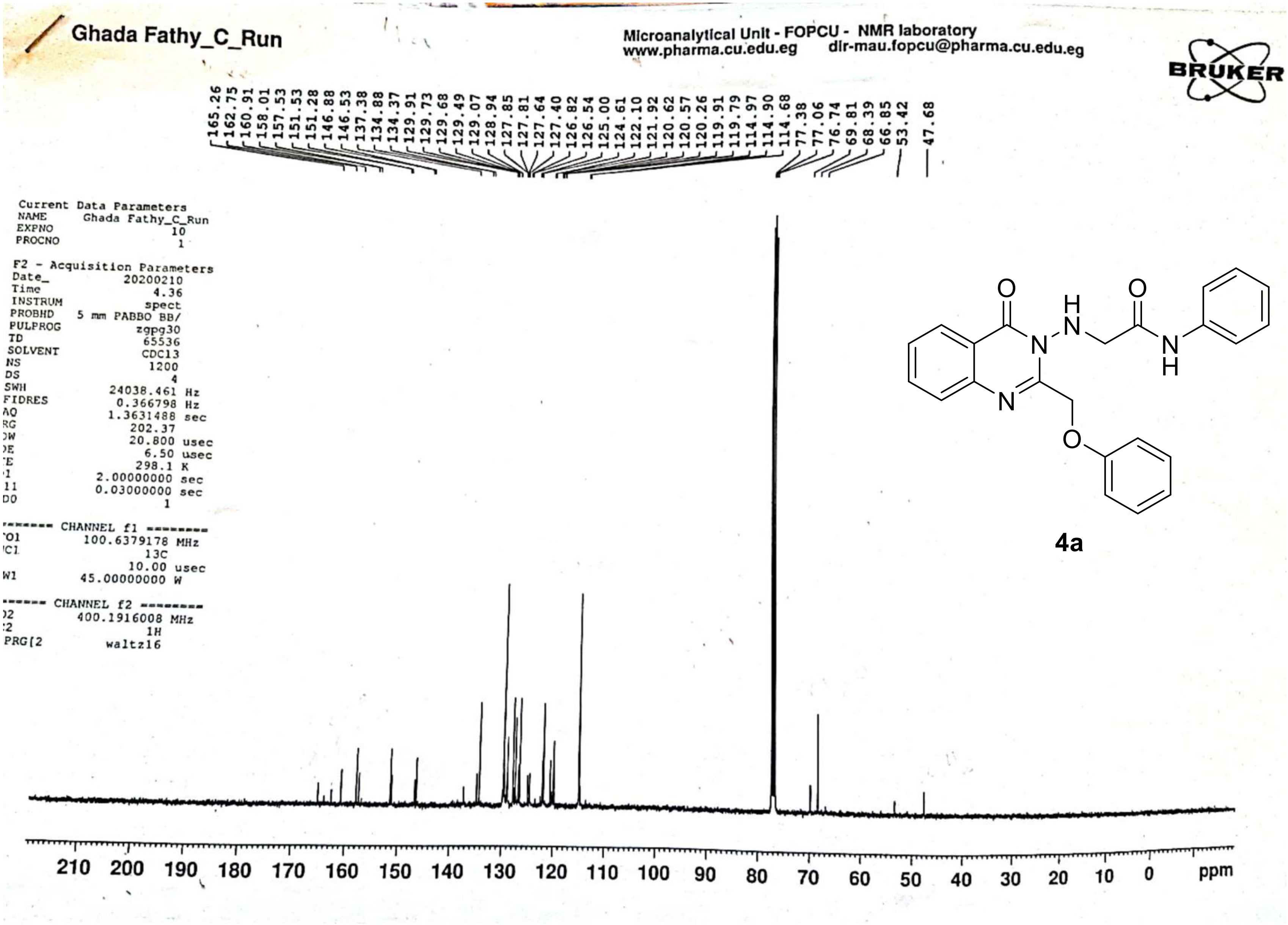

CS Scanned with CamScanner

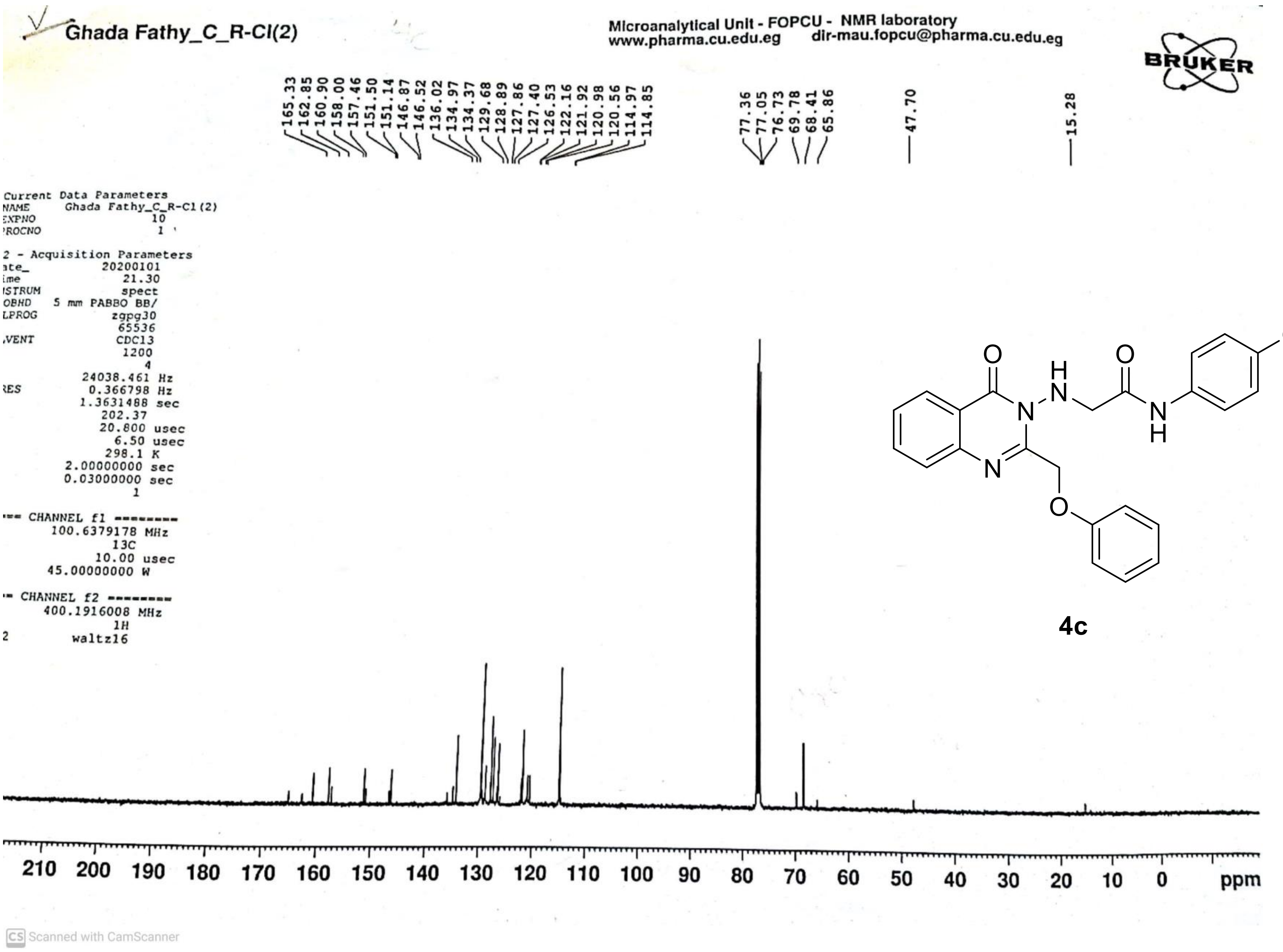

Ghada Fathy_C_R-Cl(2)
Microanalytical Unit - FOPCU - NMR laboratory
www.pharma.cu.edu.eg dir-mau.fopcu@pharma.cu.edu.eg
BRUKER
165.33 162.85 160.90 158.00 157.46 151.50 151.14 146.87 146.52 136.02 134.97 134.37 129.68 128.89 127.86 127.40 126.53 122.16 121.92 120.98 120.56 114.97 114.85
77.36 77.05 76.73 69.78 68.41 65.86
47.70
15.28
Current Data Parameters
NAME Ghada Fathy_C_R-Cl(2)
EXPNO 10
PROCNO 1
F2 - Acquisition Parameters
Date_ 20200101
Time 21.30
INSTRUM spect
PROBHD 5 mm PABBO BB/
PULPROG zgpg30
65536
SOLVENT CDCl3
1200
4
24038.461 Hz
FIDRES 0.366798 Hz
1.3631488 sec
202.37
20.800 usec
6.50 usec
298.1 K
2.00000000 sec
0.03000000 sec
1
CHANNEL f1
100.6379178 MHz
13C
10.00 usec
45.00000000 W
CHANNEL f2
400.1916008 MHz
1H
waltz16
O
O
H
N
N
N
N
H
Cl
O
4c
210 200 190 180 170 160 150 140 130 120 110 100 90 80 70 60 50 40 30 20 10 0 ppm
Scanned with CamScanner

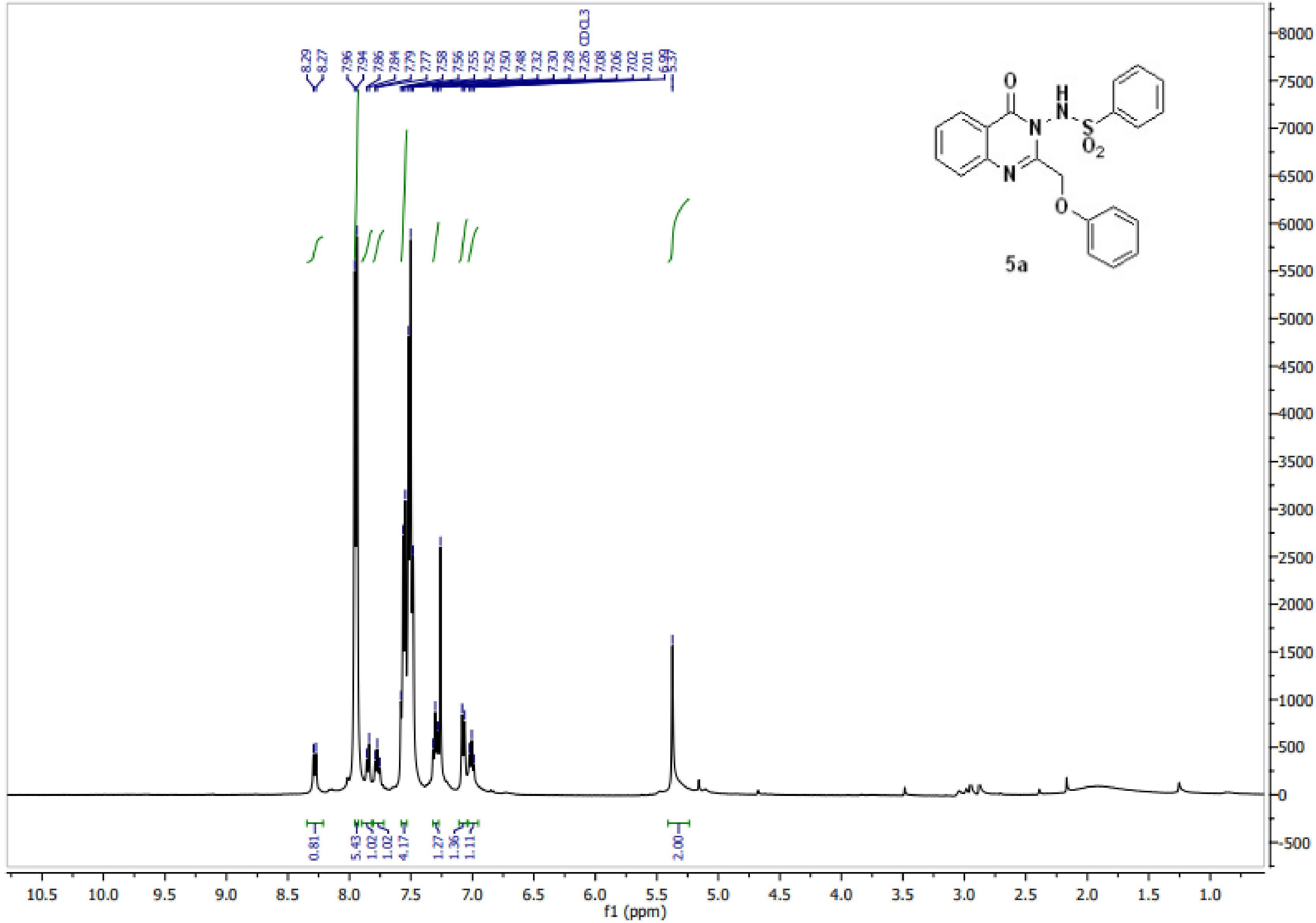
8.29
8.27
7.96
7.94
7.86
7.84
7.79
7.77
7.58
7.56
7.55
7.52
7.50
7.48
7.32
7.30
7.28
7.26 CDCL3
7.08
7.06
7.02
7.01
6.99
5.37
0.81
5.43
1.02
1.02
4.17
1.27
1.36
1.11
2.00
5a
f1 (ppm)

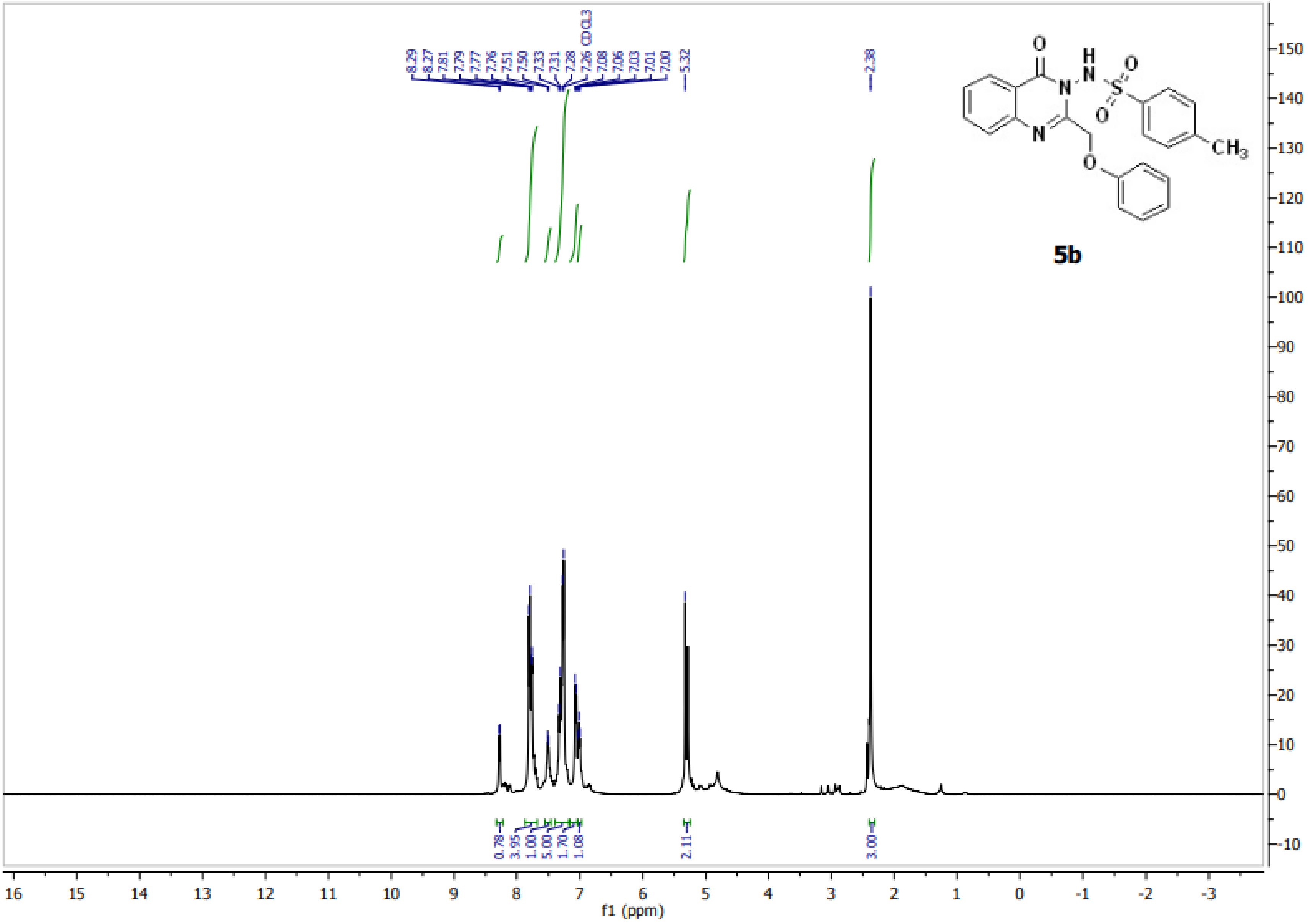

5b

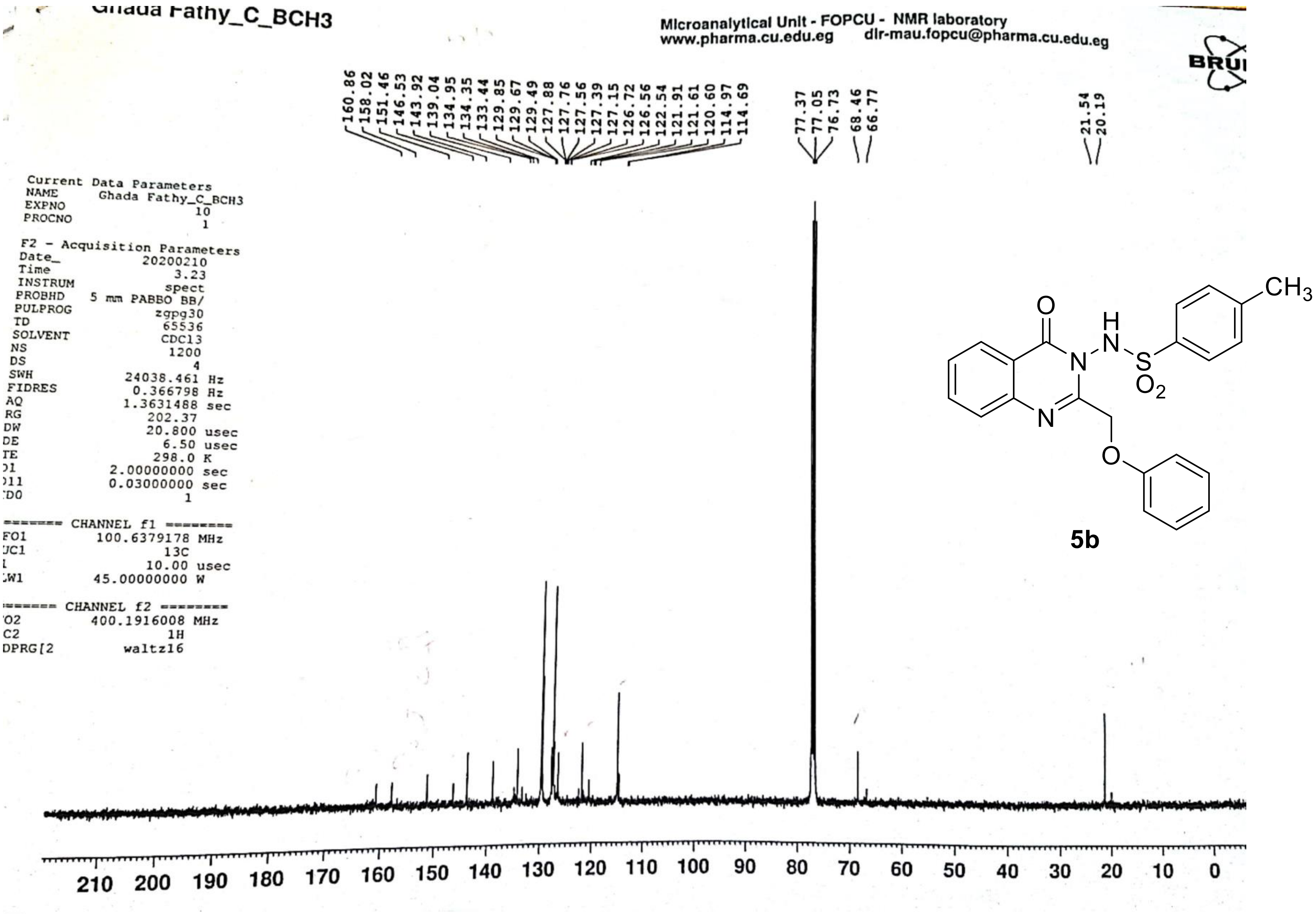

Scanned with CamScanner

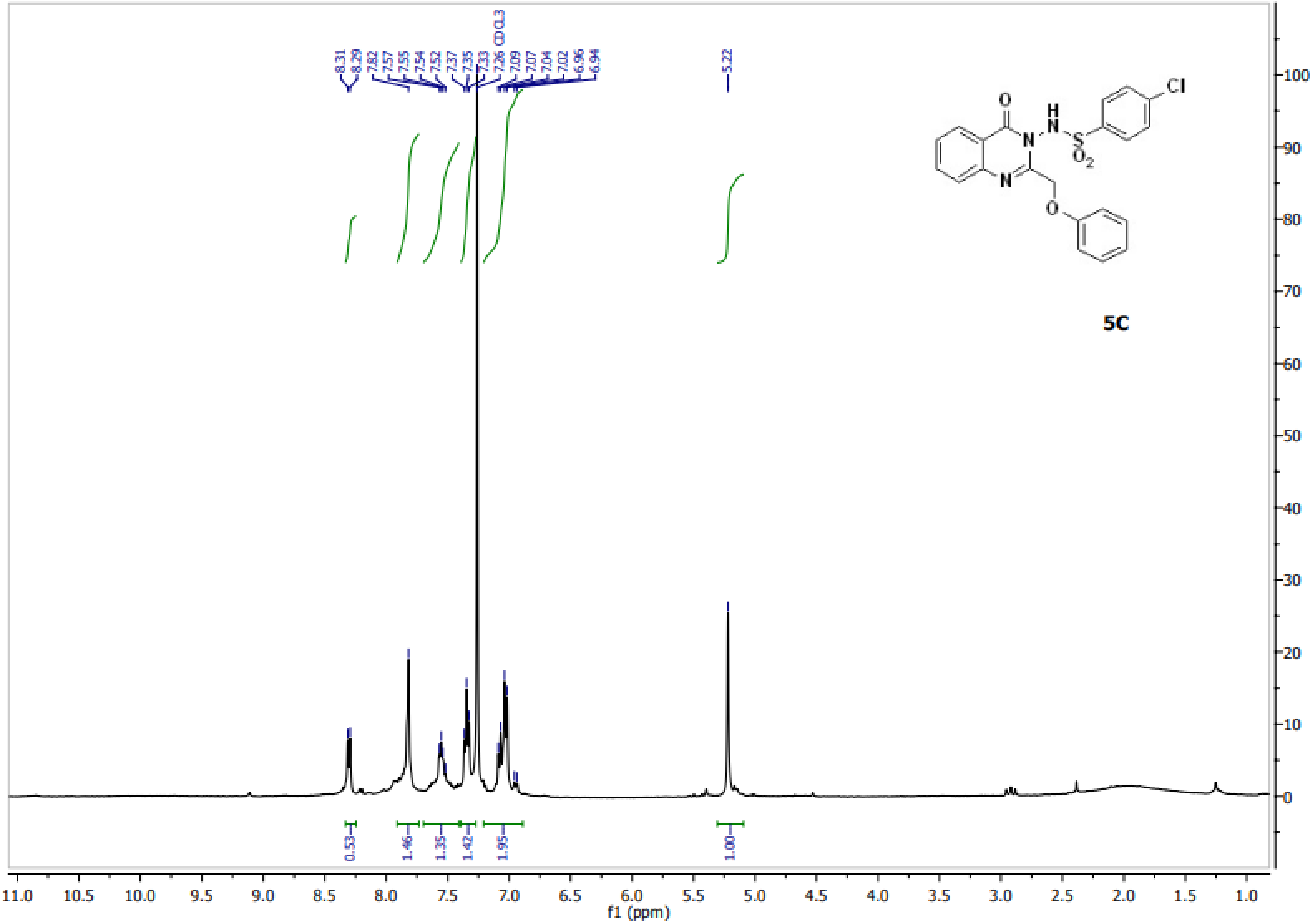
8.31
8.29
7.82
7.57
7.55
7.54
7.52
7.37
7.35
7.33
7.26 CDCl3
7.09
7.07
7.04
7.02
6.96
6.94
5.22
Cl
5C
0.53
1.46
1.35
1.42
1.95
1.00
f1 (ppm)

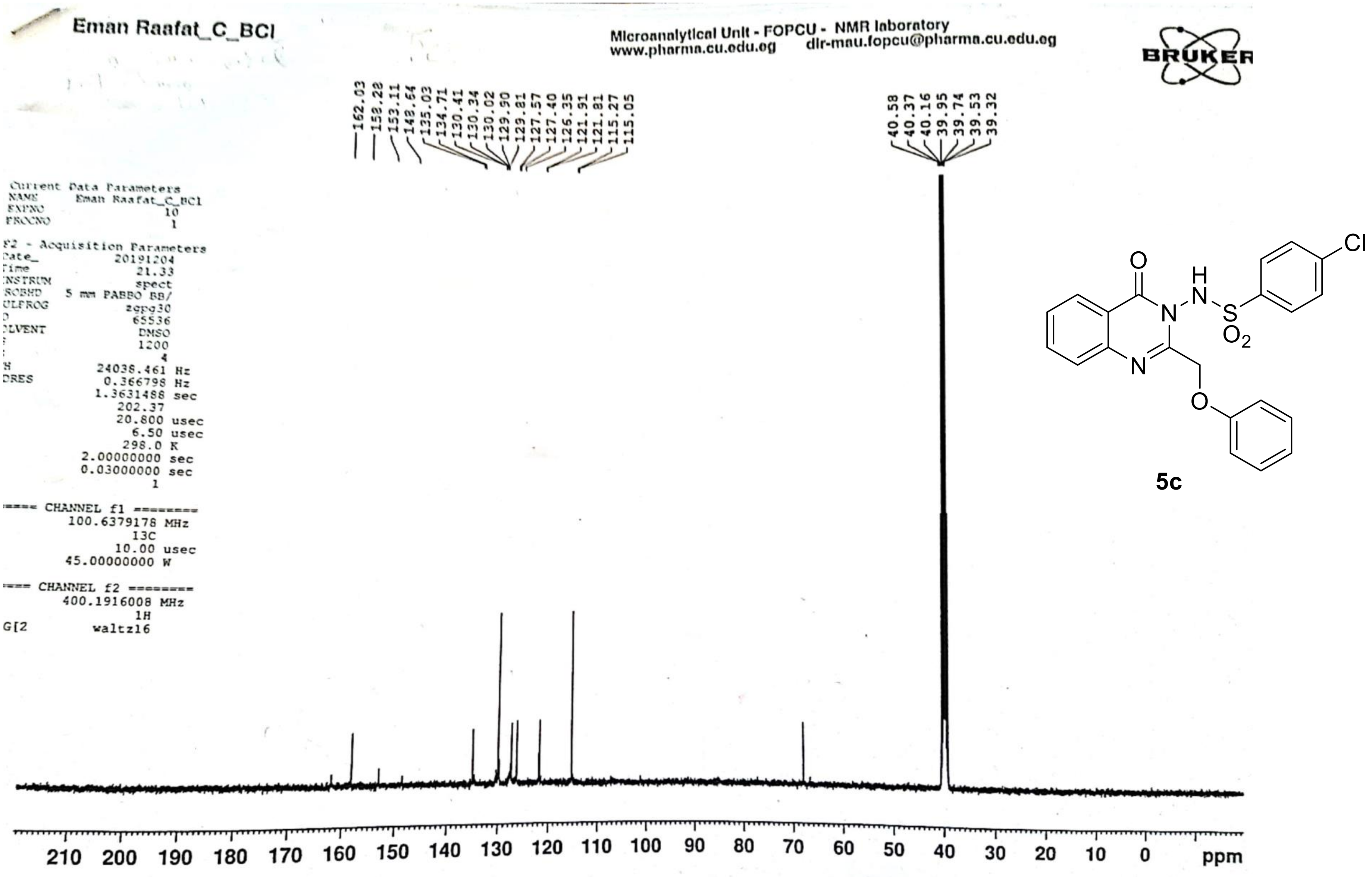

5c

Scanned with CamScanner

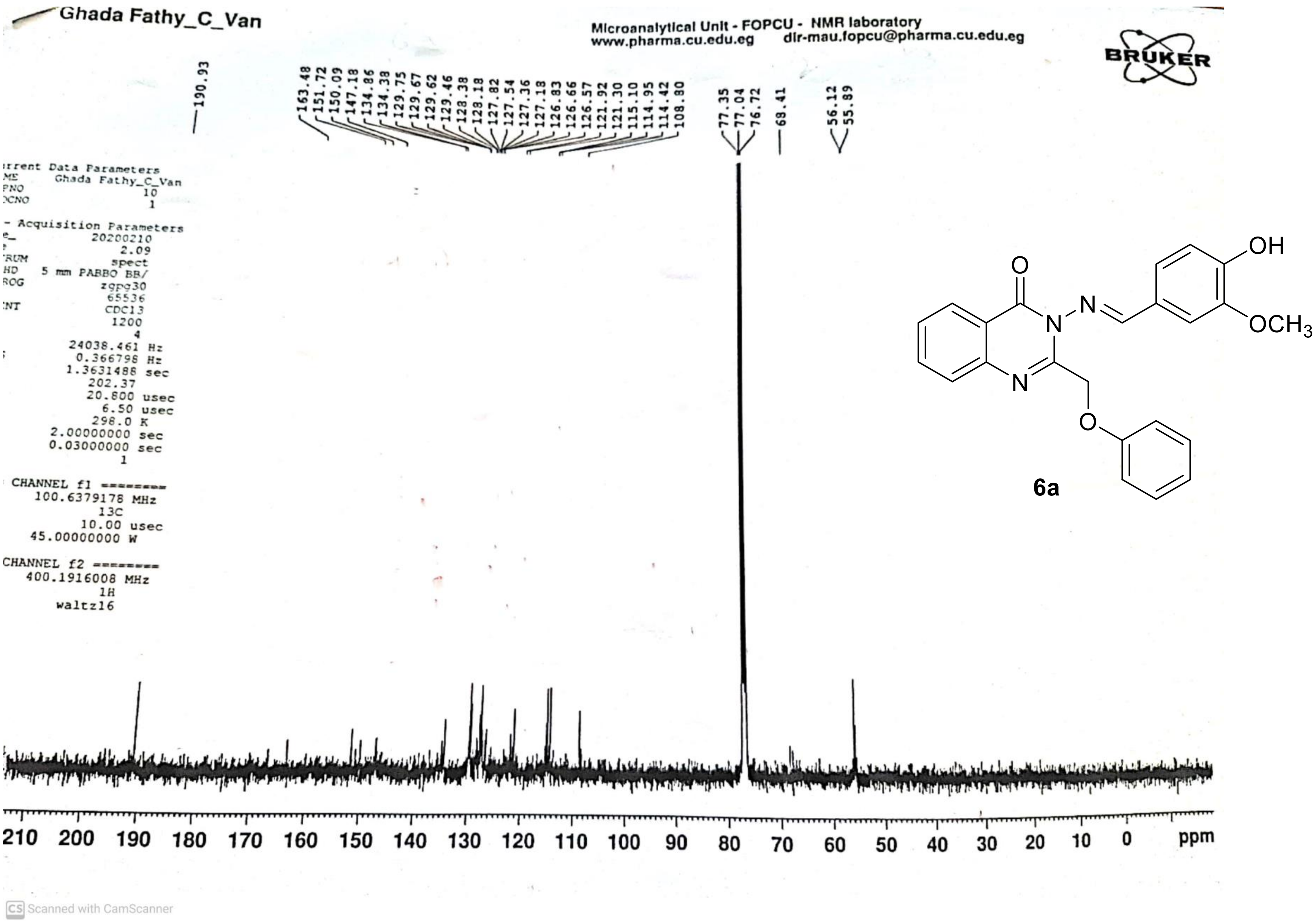
Ghada Fathy_C_Van
Microanalytical Unit - FOPCU - NMR laboratory
www.pharma.cu.edu.eg dir-mau.fopcu@pharma.cu.edu.eg
BRUKER
190.93
163.48
151.72
150.09
147.18
134.86
134.38
129.75
129.67
129.62
129.46
128.38
128.18
127.82
127.54
127.36
127.18
126.83
126.66
126.57
121.92
121.30
115.10
114.95
114.42
108.80
77.35
77.04
76.72
68.41
56.12
55.89
Current Data Parameters
Ghada Fathy_C_Van
10
1
Acquisition Parameters
20200210
2.09
spect
5 mm PABBO BB/
zgpg30
65536
CDCl3
1200
4
24038.461 Hz
0.366798 Hz
1.3631488 sec
202.37
20.800 usec
6.50 usec
298.0 K
2.00000000 sec
0.03000000 sec
1
CHANNEL f1 ========
100.6379178 MHz
13C
10.00 usec
45.00000000 W
CHANNEL f2 ========
400.1916008 MHz
1H
waltz16
210 200 190 180 170 160 150 140 130 120 110 100 90 80 70 60 50 40 30 20 10 0 ppm
Scanned with CamScanner
O
N
N
N
O
OH
OCH3

6a

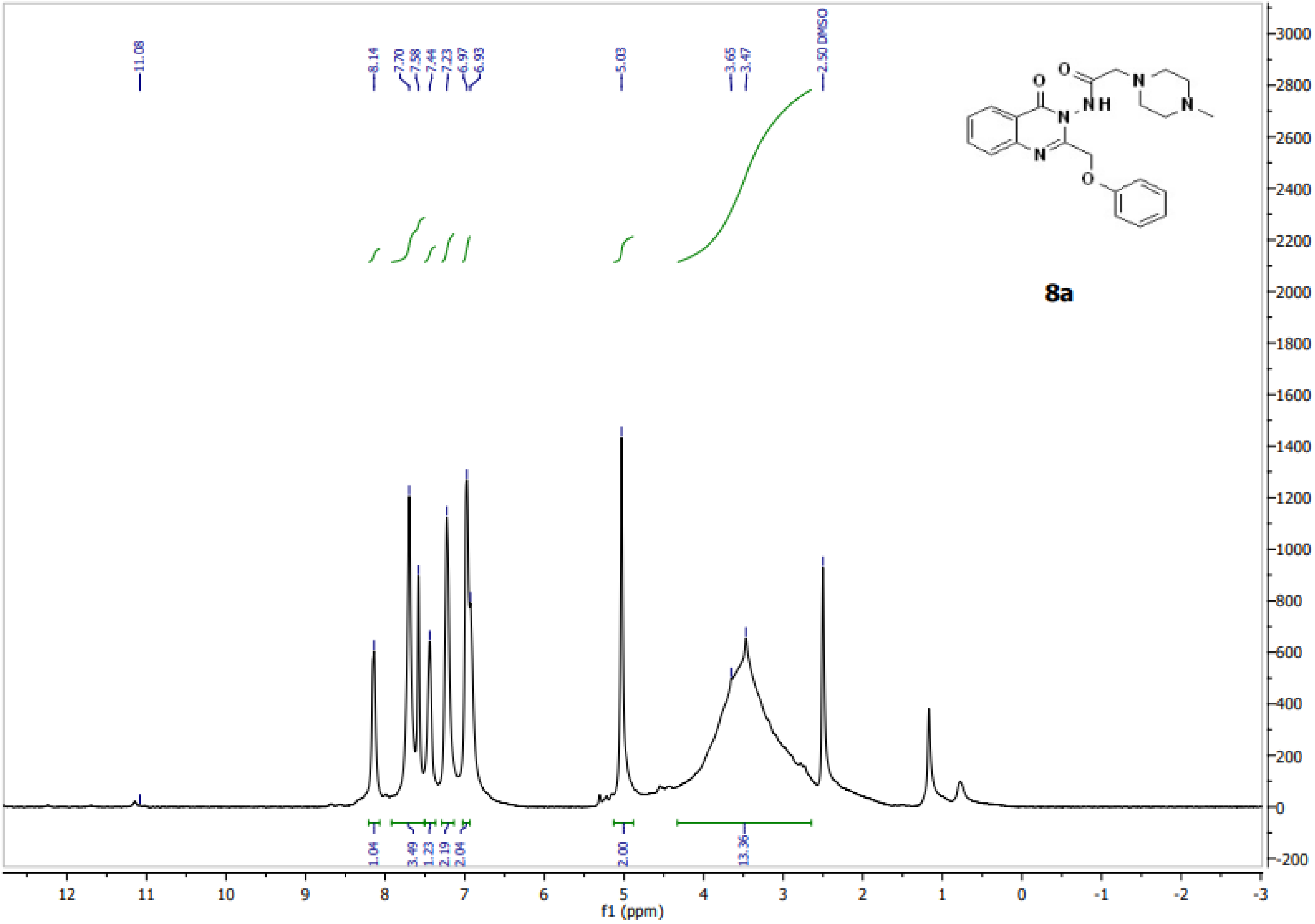
11.08
8.14
7.70
7.58
7.44
7.23
6.97
6.93
5.03
3.65
3.47
2.50 DMSO
1.04
3.49
1.23
2.19
2.04
2.00
13.36
8a
f1 (ppm)

Eman Raafat_C_NCH3

Microanalytical Unit - FOPCU - NMR laboratory
www.pharma.cu.edu.eg dir-mau.fopcu@pharma.cu.edu.eg

BRUKER

```
:urrent Data Parameters
JAME        Eman Raafat_C_NCH3
:XPNO                 10
ROCNO                  1

2 - Acquisition Parameters
ate_            20201119
ime                15.02
NSTRUM             spect
ROBHD     5 mm PABBO BB/
JLPROG           zgpg30
)                  65536
)LVENT             CDCl3
:                   1500
                       4
H           24038.461 Hz
DRES         0.366798 Hz
            1.3631488 sec
               202.37
               20.800 usec
                 6.50 usec
                298.1 K
           2.00000000 sec
           0.03000000 sec
                    1

===== CHANNEL f1 ========
1         100.6379178 MHz
1                 13C
                10.00 usec
1         45.00000000 W

===== CHANNEL f2 ========
2         400.1916008 MHz
2                  1H
PRG[2          waltz16
```

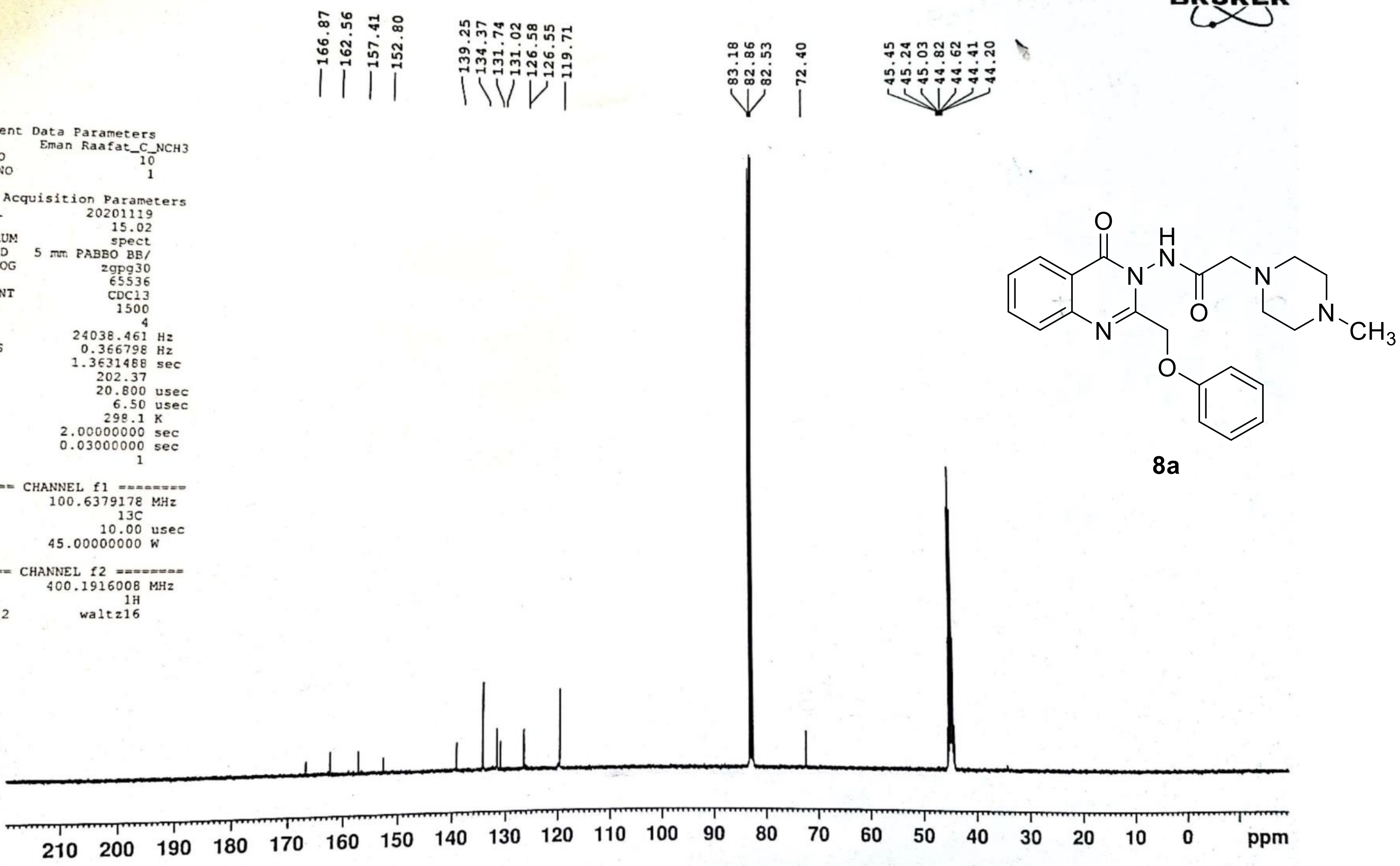

8a

CS Scanned with CamScanner

Eman Raafat_C_L1

Microanalytical Unit - FOPCU - NMR laboratory
www.pharma.cu.edu.eg dir-mau.fopcu@pharma.cu.edu.eg

BRUKER

```
Current Data Parameters
NAME        Eman Raafat_C_L1
EXPNO                     10
PROCNO                     1

F2 - Acquisition Parameters
Date_               20200924
Time                   10.17
INSTRUM                spect
?ROBHD     5 mm PABBO BB/
?ULPROG              zgpg30
:D                    65536
:OLVENT               CDCl3
'S                     1200
S                         4
WH              24038.461 Hz
IDRES            0.366798 Hz
Q               1.3631488 sec
G                   202.37
W                   20.800 usec
:                     6.50 usec
:            0 K
              2.00000000 sec
1             0.03000000 sec
0                        1

====== CHANNEL f1 ========
O1            100.6379178 MHz
C1                    13C
                    10.00 usec
W1            45.00000000 W

====== CHANNEL f2 ========
O2            400.1916008 MHz
C2                     1H
PRG[2              waltz16
```

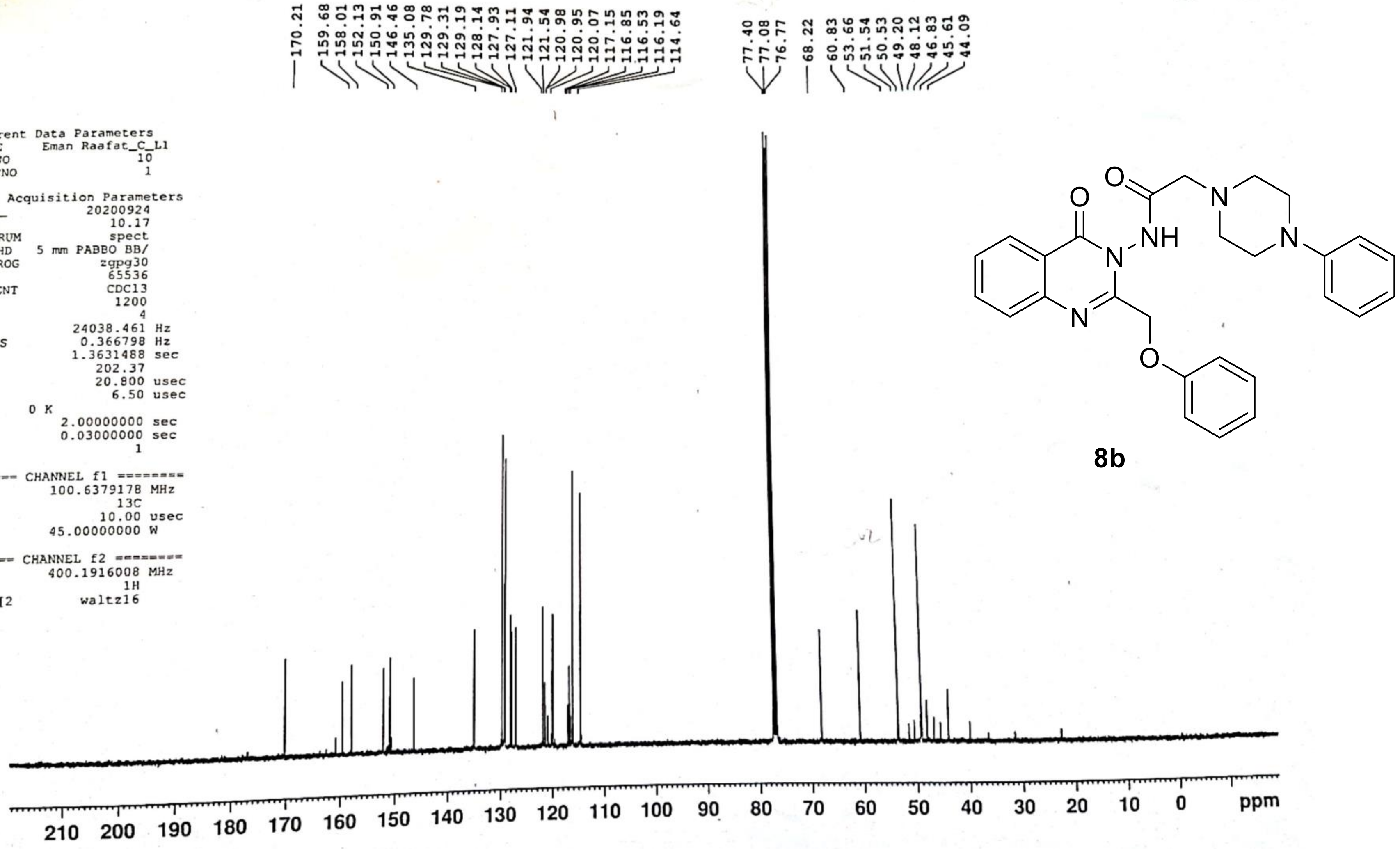

8b

Scanned with CamScanner

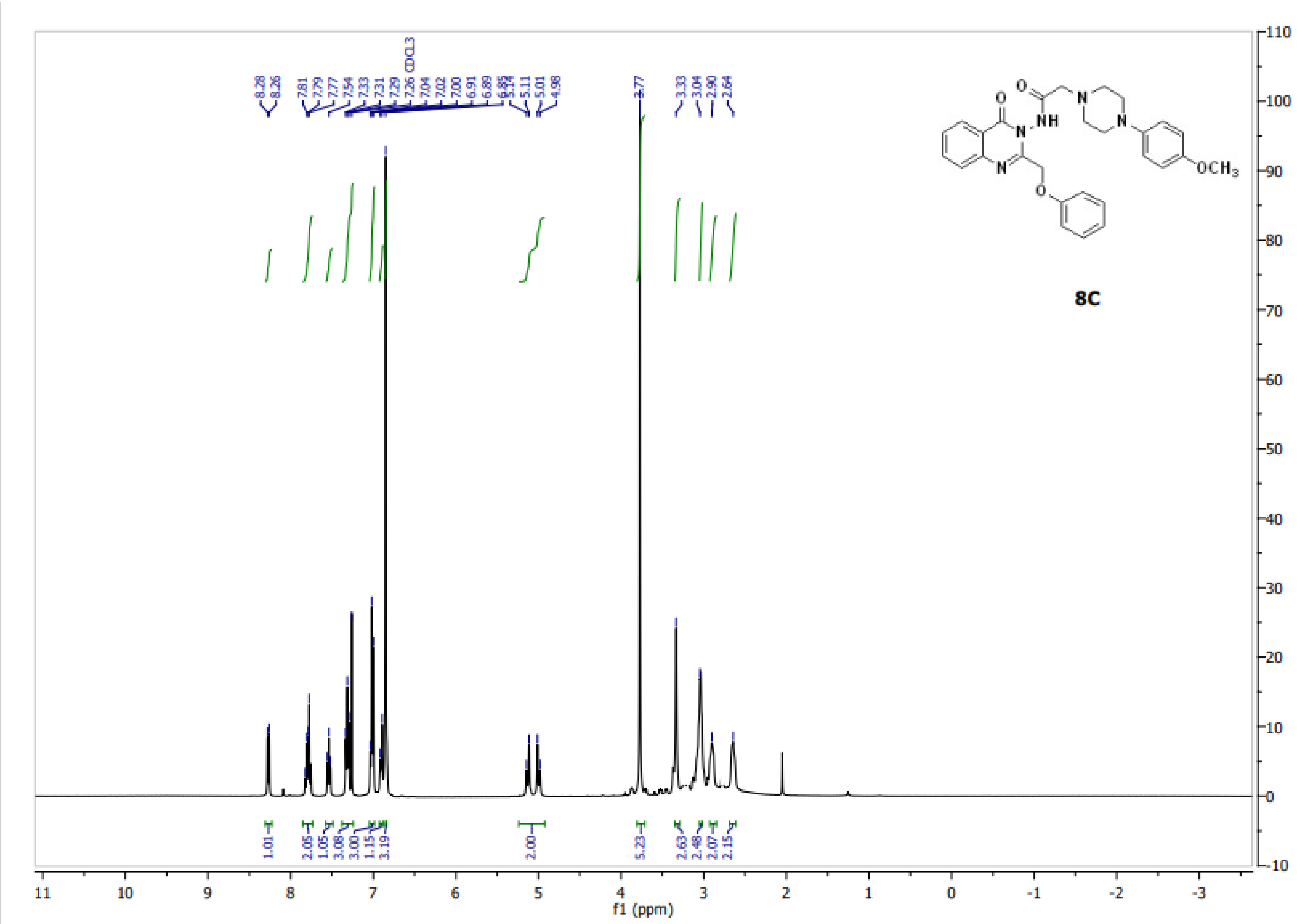
8.28
8.26
7.81
7.79
7.77
7.54
7.33
7.31
7.29
7.26
CDCL3
7.04
7.02
7.00
6.91
6.89
5.14
5.11
5.01
4.98
3.77
3.33
3.04
2.90
2.64
1.01
2.05
1.05
3.08
3.00
1.15
3.19
2.00
5.23
2.63
2.48
2.07
2.15
O
N
NH
N
N
OCH3
8C
f1 (ppm)

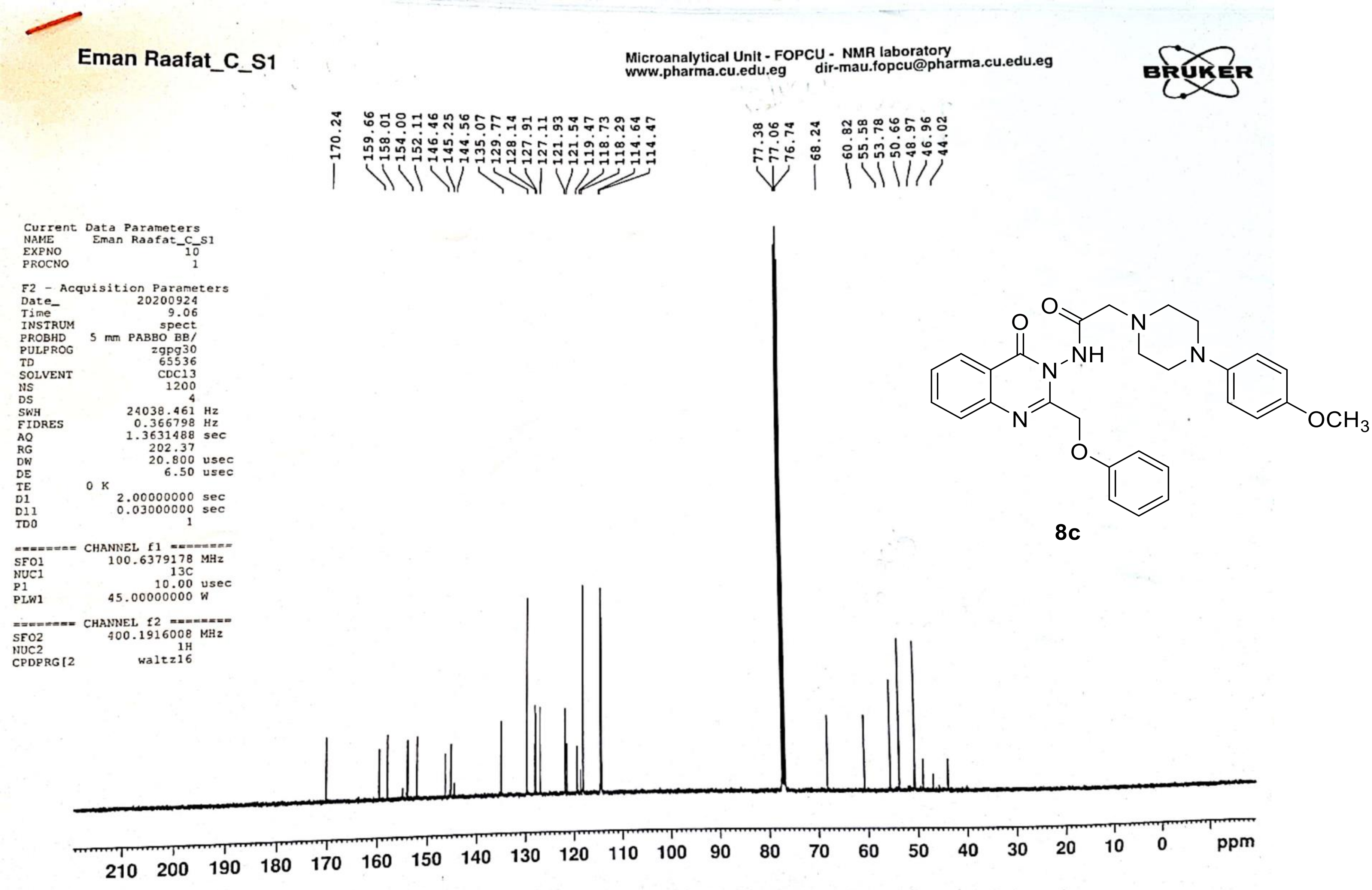

8c

CS Scanned with CamScanner

10. Mass spectra

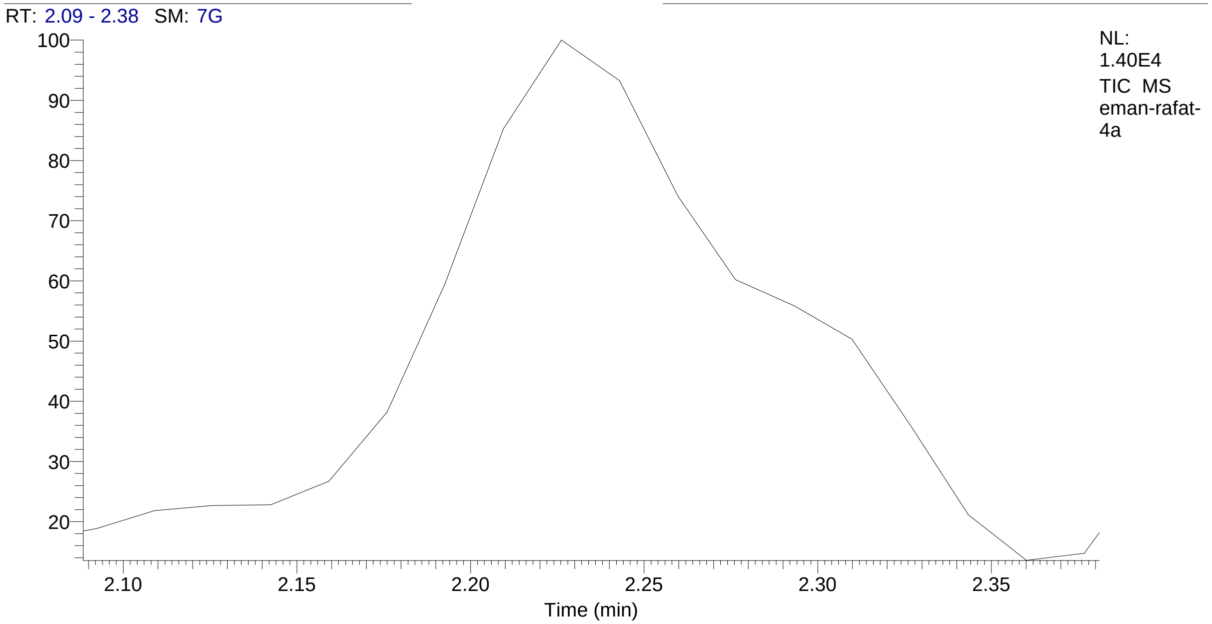
RT: 2.09 - 2.38 SM: 7G
NL: 1.40E4 TIC MS eman-rafat-4a
Time (min)

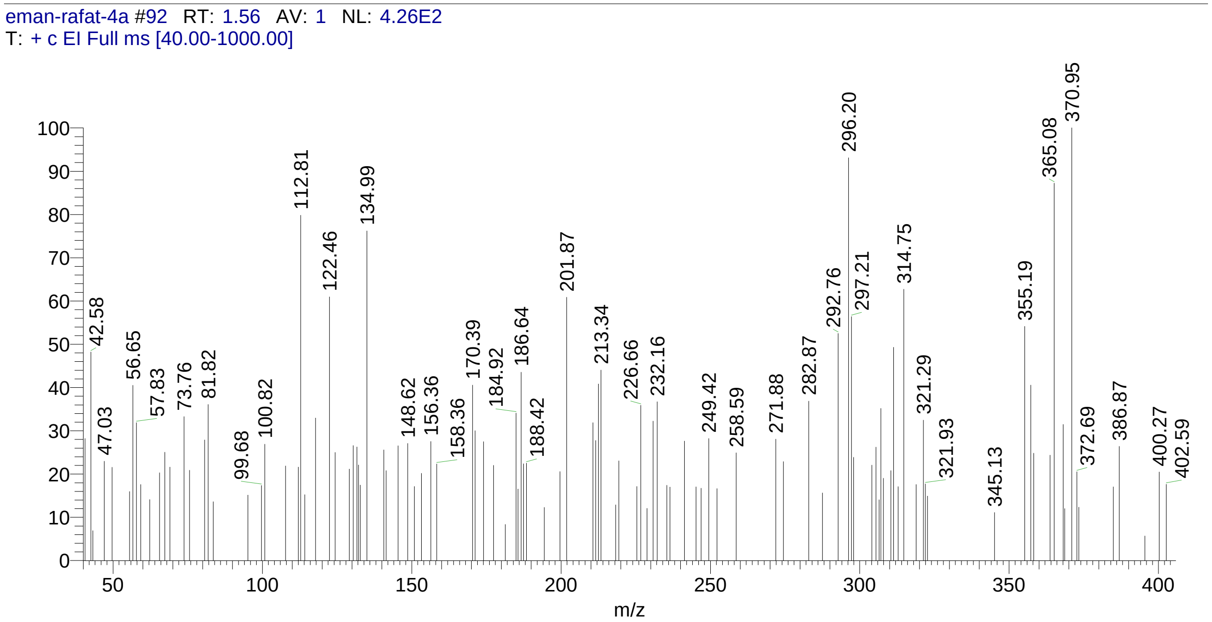
eman-rafat-4a #92 RT: 1.56 AV: 1 NL: 4.26E2
T: + c EI Full ms [40.00-1000.00]
42.58
47.03
56.65
57.83
73.76
81.82
99.68
100.82
112.81
122.46
134.99
148.62
156.36
158.36
170.39
184.92
186.64
188.42
201.87
213.34
226.66
232.16
249.42
258.59
271.88
282.87
292.76
296.20
297.21
314.75
321.29
321.93
345.13
355.19
365.08
370.95
372.69
386.87
400.27
402.59
m/z

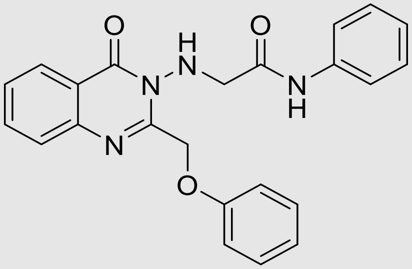
O
H
N
N
O
N
H
N
O

4a

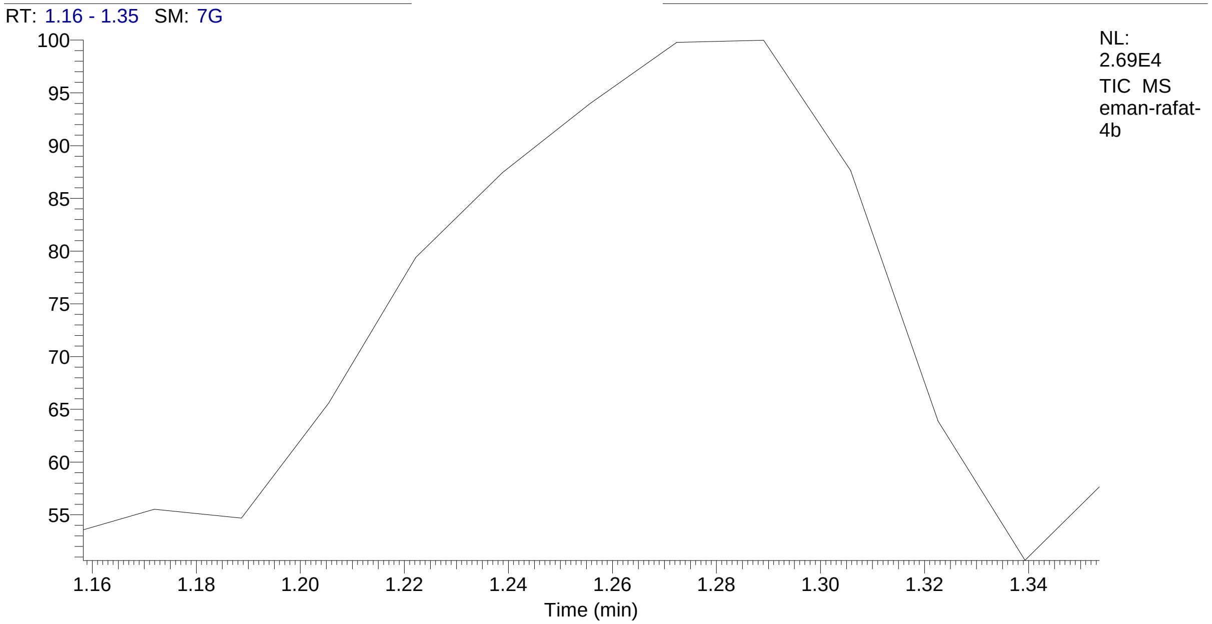
RT: 1.16 - 1.35 SM: 7G
NL: 2.69E4 TIC MS eman-rafat-4b
Time (min)

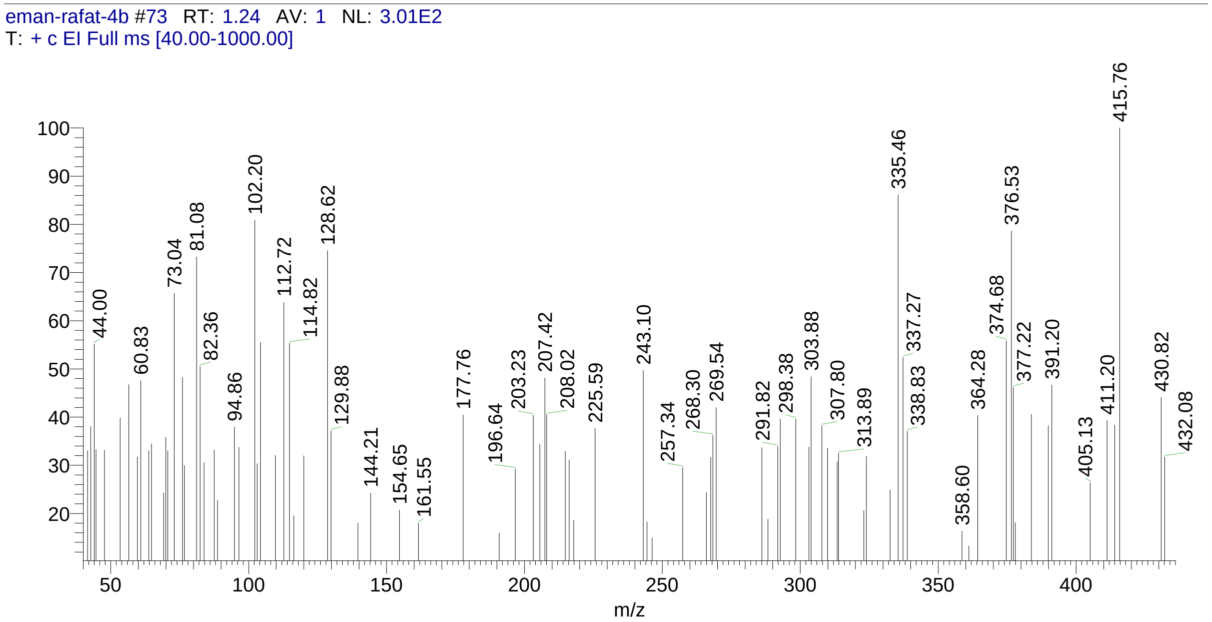
eman-rafat-4b #73 RT: 1.24 AV: 1 NL: 3.01E2
T: + c EI Full ms [40.00-1000.00]
44.00
60.83
73.04
81.08
82.36
94.86
102.20
112.72
114.82
128.62
129.88
144.21
154.65
161.55
177.76
196.64
203.23
207.42
208.02
225.59
243.10
257.34
268.30
269.54
291.82
298.38
303.88
307.80
313.89
335.46
337.27
338.83
358.60
364.28
374.68
376.53
377.22
391.20
405.13
411.20
415.76
430.82
432.08
m/z

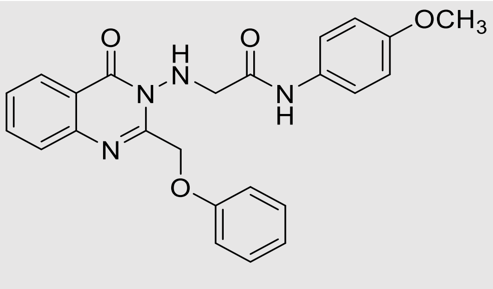
O
H
N
O
N
H
OCH3
N
N
O

4b

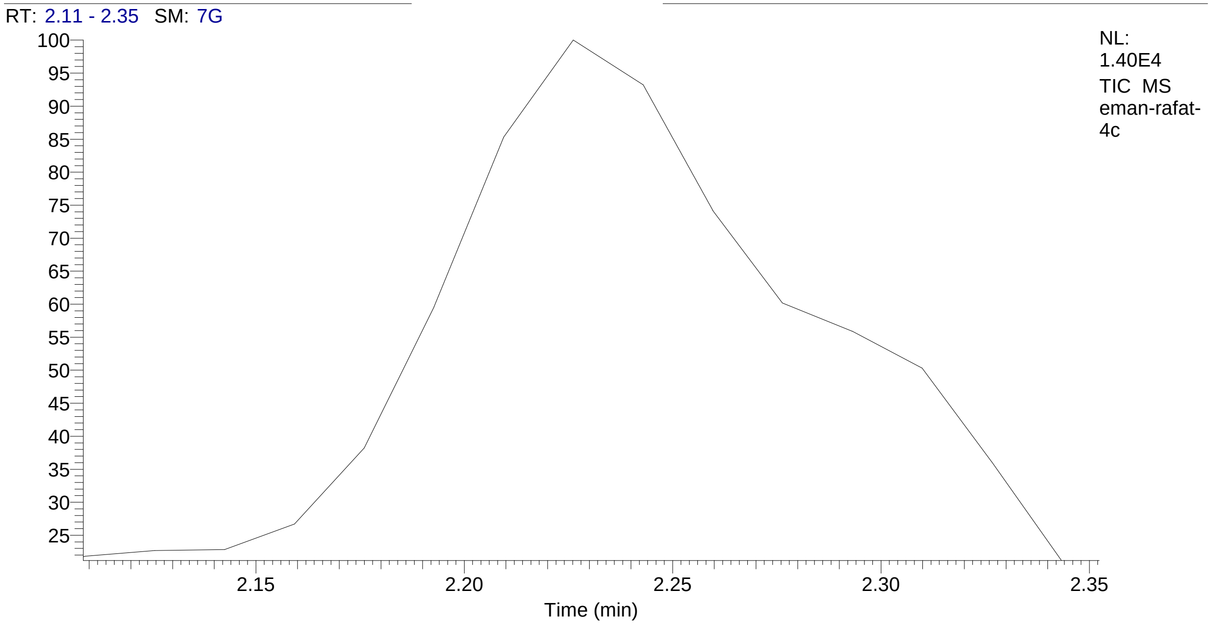
RT: 2.11 - 2.35 SM: 7G
NL:
1.40E4
TIC MS
eman-rafat-
4c
Time (min)

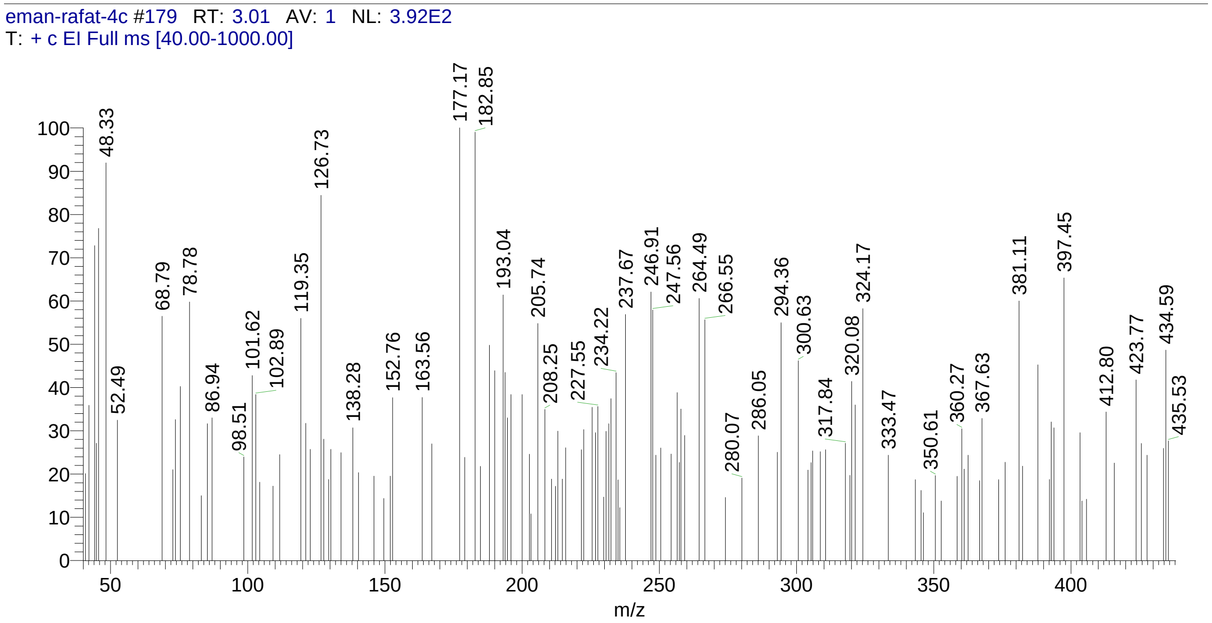
eman-rafat-4c #179 RT: 3.01 AV: 1 NL: 3.92E2
T: + c EI Full ms [40.00-1000.00]
48.33
52.49
68.79
78.78
86.94
98.51
101.62
102.89
119.35
126.73
138.28
152.76
163.56
177.17
182.85
193.04
205.74
208.25
227.55
234.22
237.67
246.91
247.56
264.49
266.55
280.07
286.05
294.36
300.63
317.84
320.08
324.17
333.47
350.61
360.27
367.63
381.11
397.45
412.80
423.77
434.59
435.53
m/z

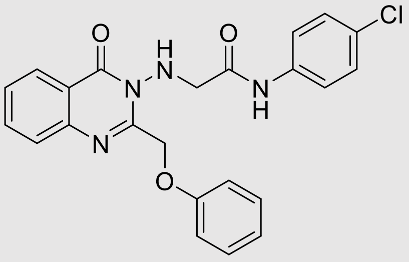
O
H
N
N
O
N
H
Cl
N
O

4c

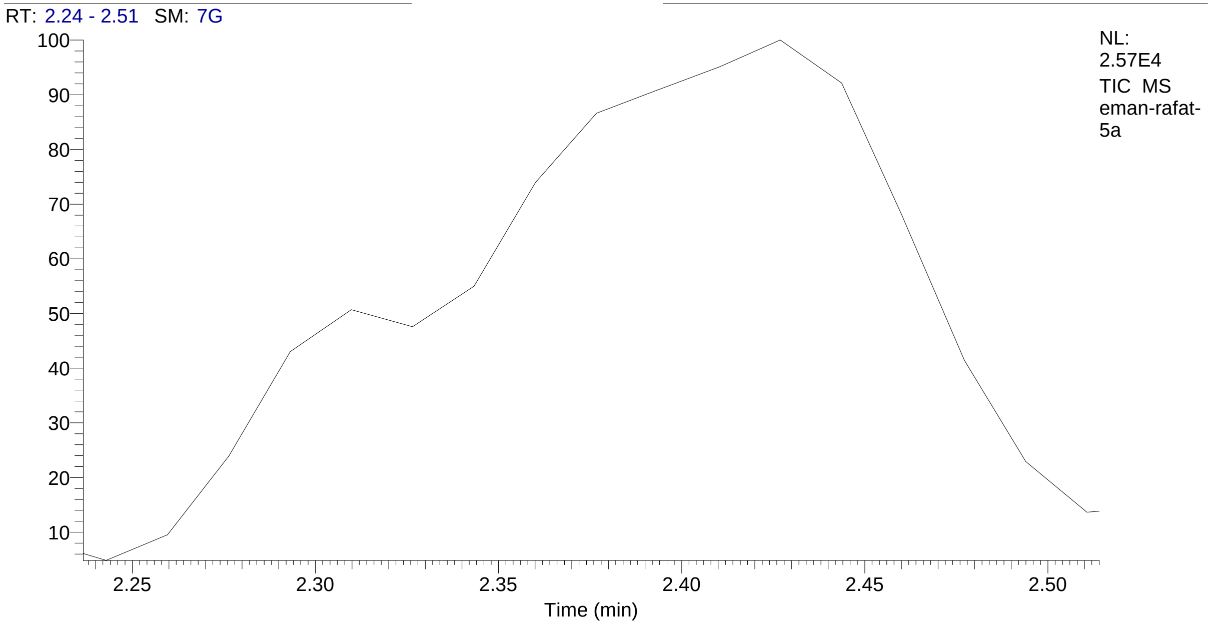
RT: 2.24 - 2.51 SM: 7G
NL: 2.57E4 TIC MS eman-rafat-5a
Time (min)

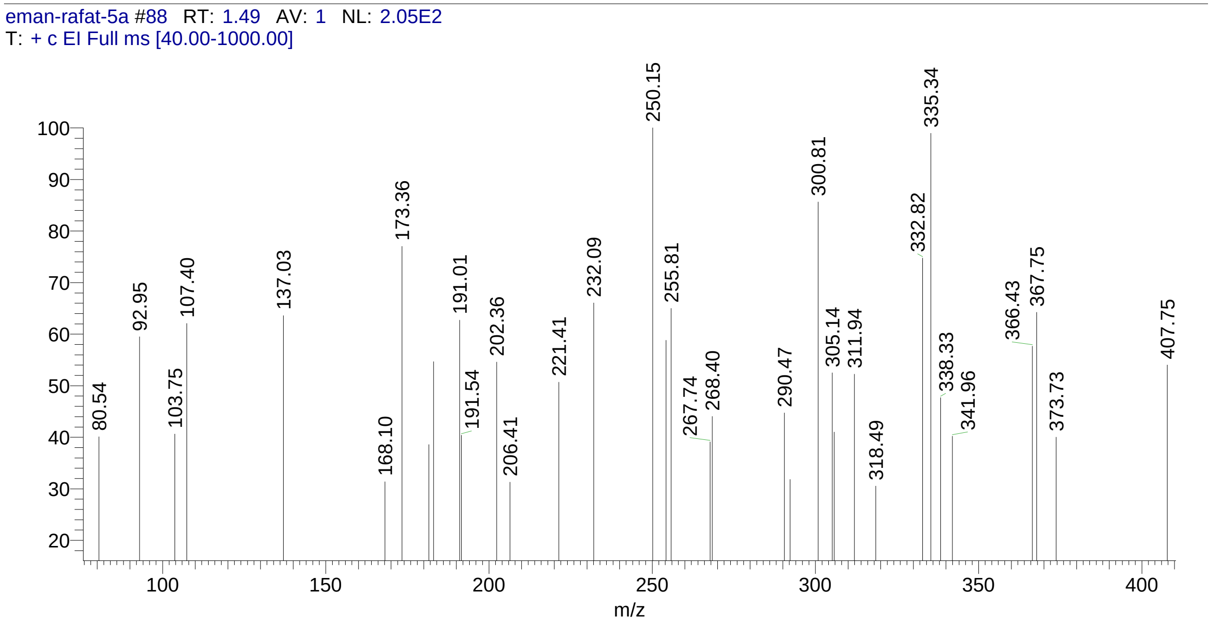
eman-rafat-5a #88 RT: 1.49 AV: 1 NL: 2.05E2
T: + c EI Full ms [40.00-1000.00]
80.54
92.95
103.75
107.40
137.03
168.10
173.36
191.01
191.54
202.36
206.41
221.41
232.09
250.15
255.81
267.74
268.40
290.47
300.81
305.14
311.94
318.49
332.82
335.34
338.33
341.96
366.43
367.75
373.73
407.75
m/z

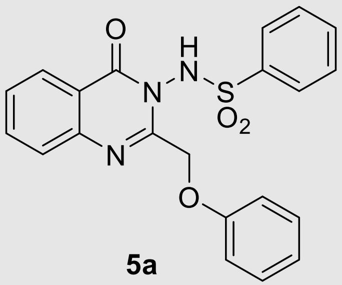
O
H
N
N
S
O2
N
O

5a

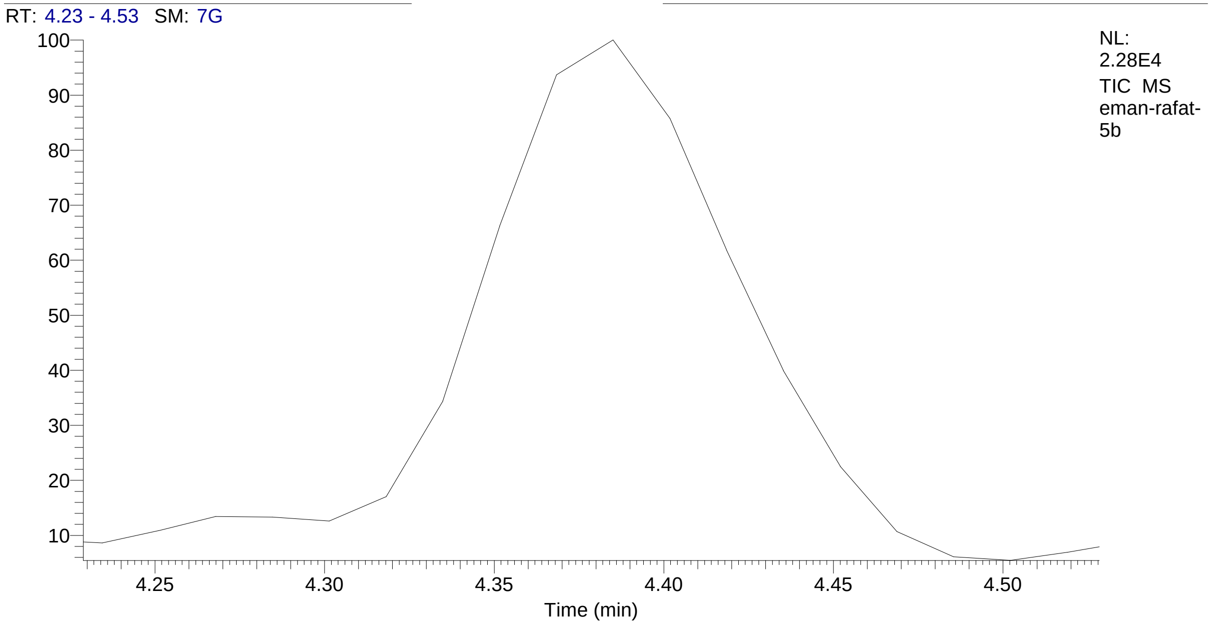
RT: 4.23 - 4.53 SM: 7G
NL: 2.28E4
TIC MS
eman-rafat-5b
Time (min)

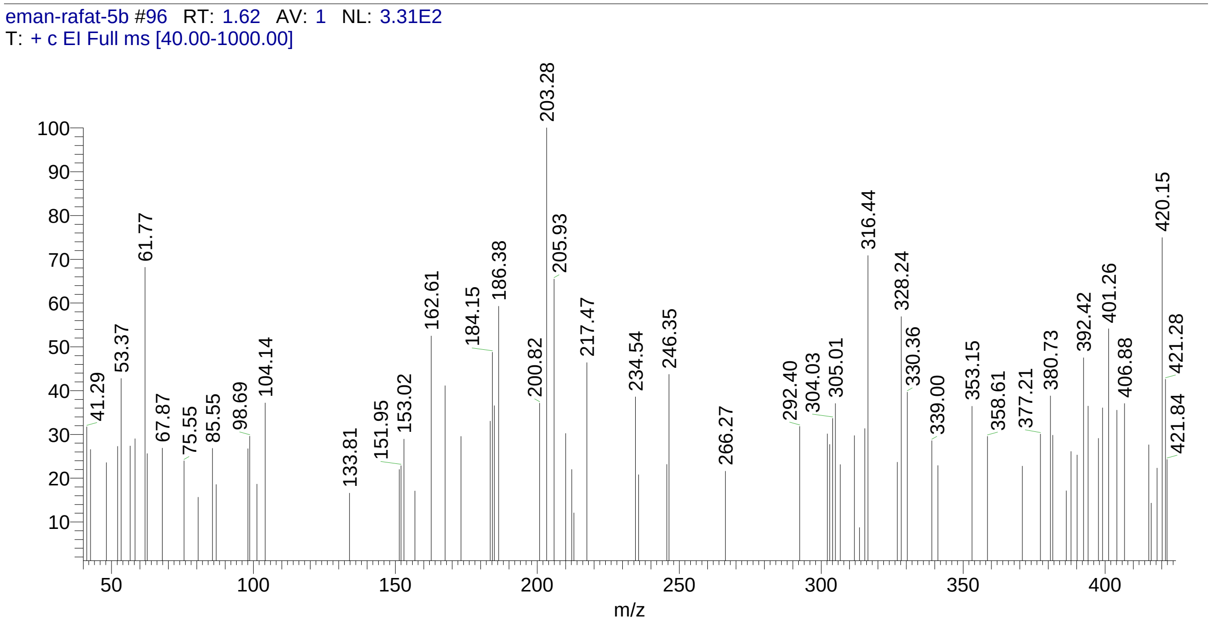
eman-rafat-5b #96 RT: 1.62 AV: 1 NL: 3.31E2
T: + c EI Full ms [40.00-1000.00]
203.28
420.15
316.44
61.77
205.93
186.38
328.24
401.26
392.42
421.28
53.37
162.61
184.15
217.47
m/z

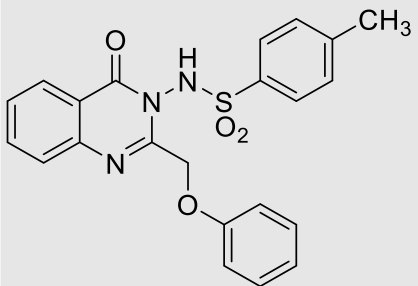

5b

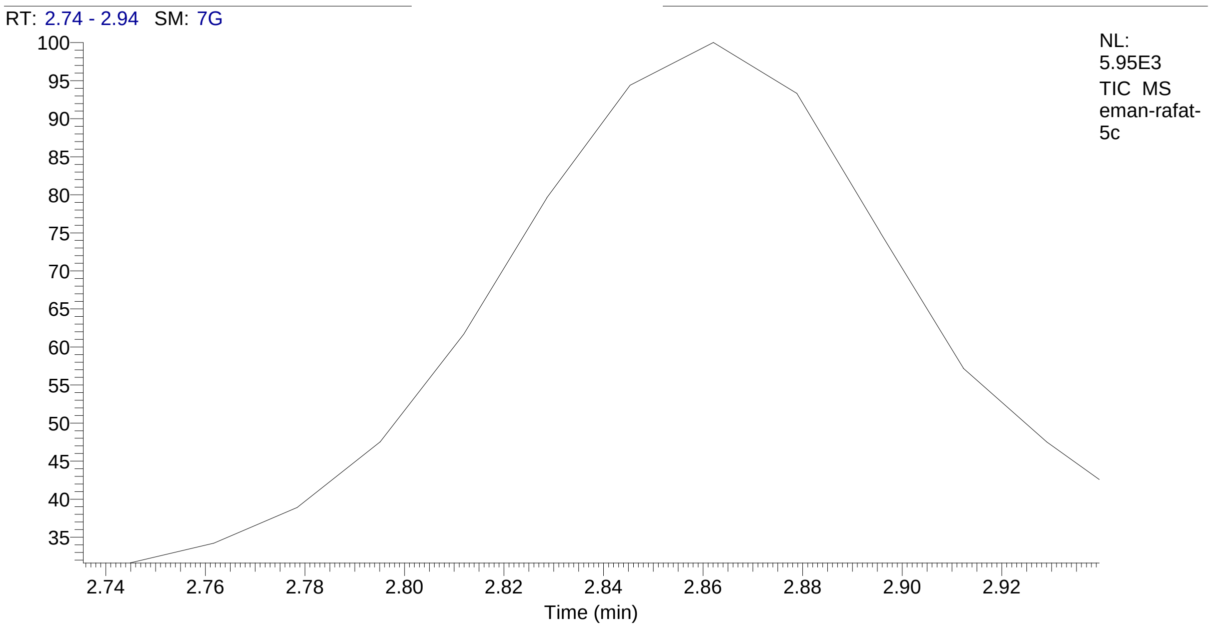
RT: 2.74 - 2.94 SM: 7G
NL:
5.95E3
TIC MS
eman-rafat-
5c
Time (min)

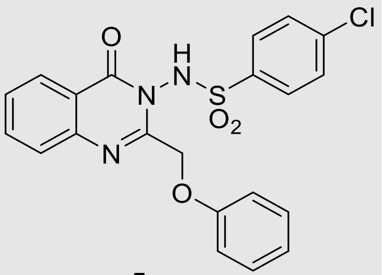

5c

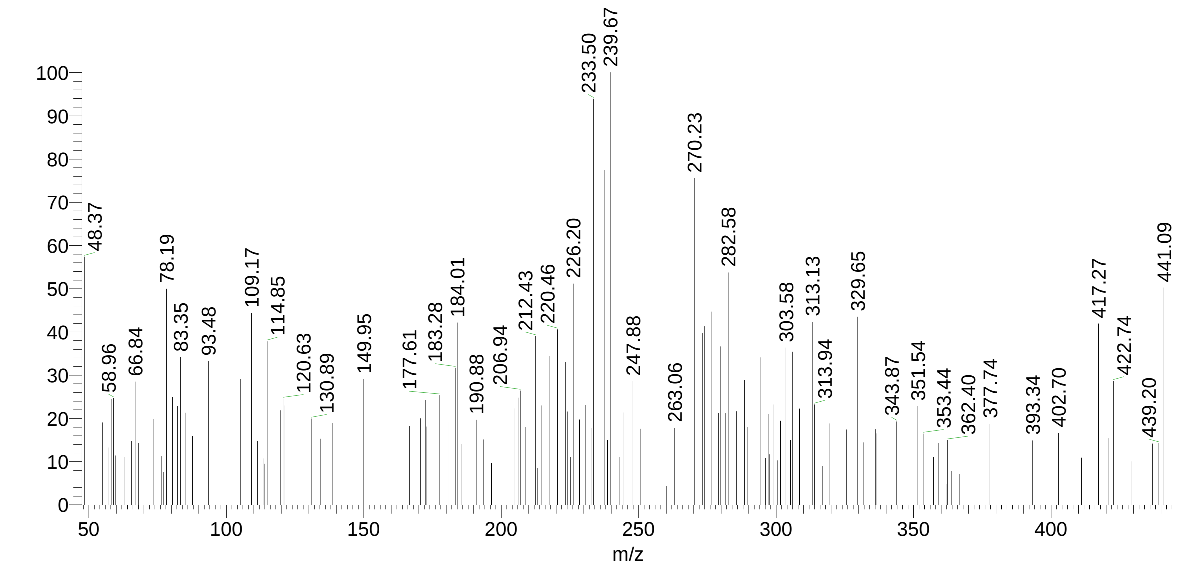
eman-rafat-5c #148 RT: 2.49 AV: 1 NL: 4.90E2
T: + c EI Full ms [40.00-1000.00]
48.37
58.96
66.84
78.19
83.35
93.48
109.17
114.85
120.63
130.89
149.95
177.61
183.28
184.01
190.88
206.94
212.43
220.46
226.20
233.50
239.67
247.88
263.06
270.23
282.58
303.58
313.13
313.94
329.65
343.87
351.54
353.44
362.40
377.74
393.34
402.70
417.27
422.74
439.20
441.09
m/z

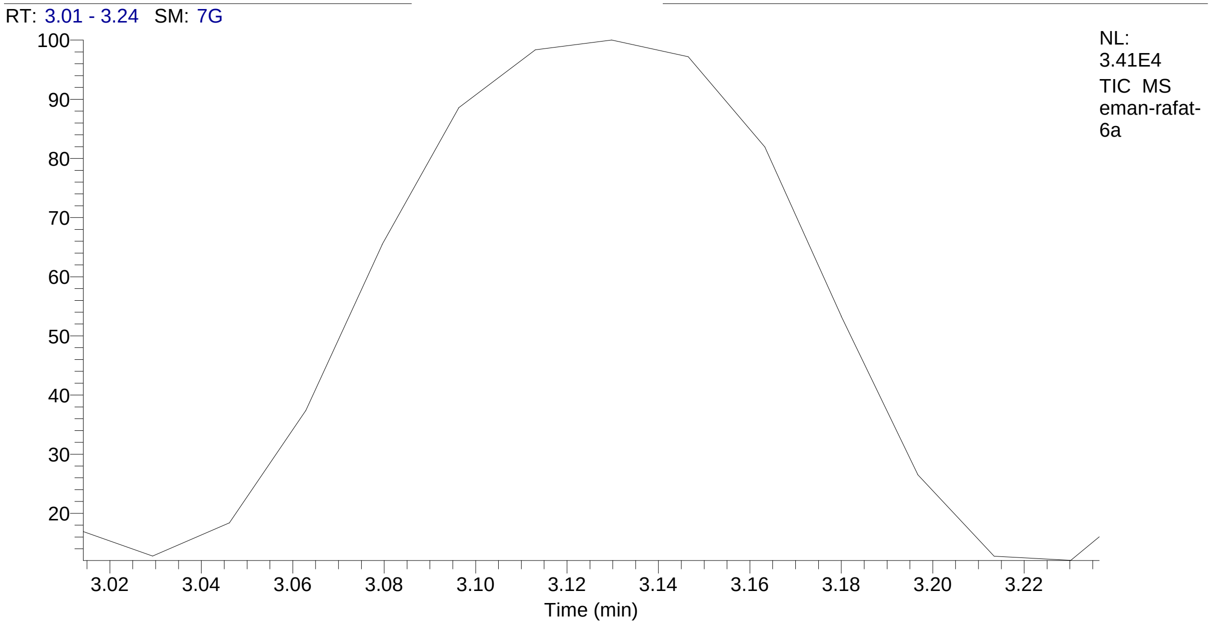
RT: 3.01 - 3.24 SM: 7G
NL: 3.41E4
TIC MS eman-rafat-6a
Time (min)

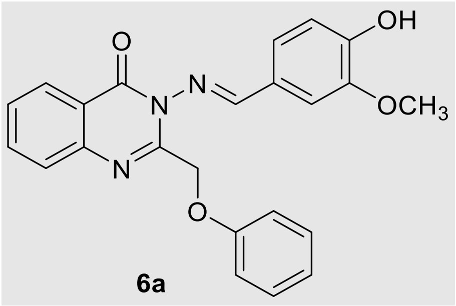
O
N
N
N
OH
OCH3
O
6a

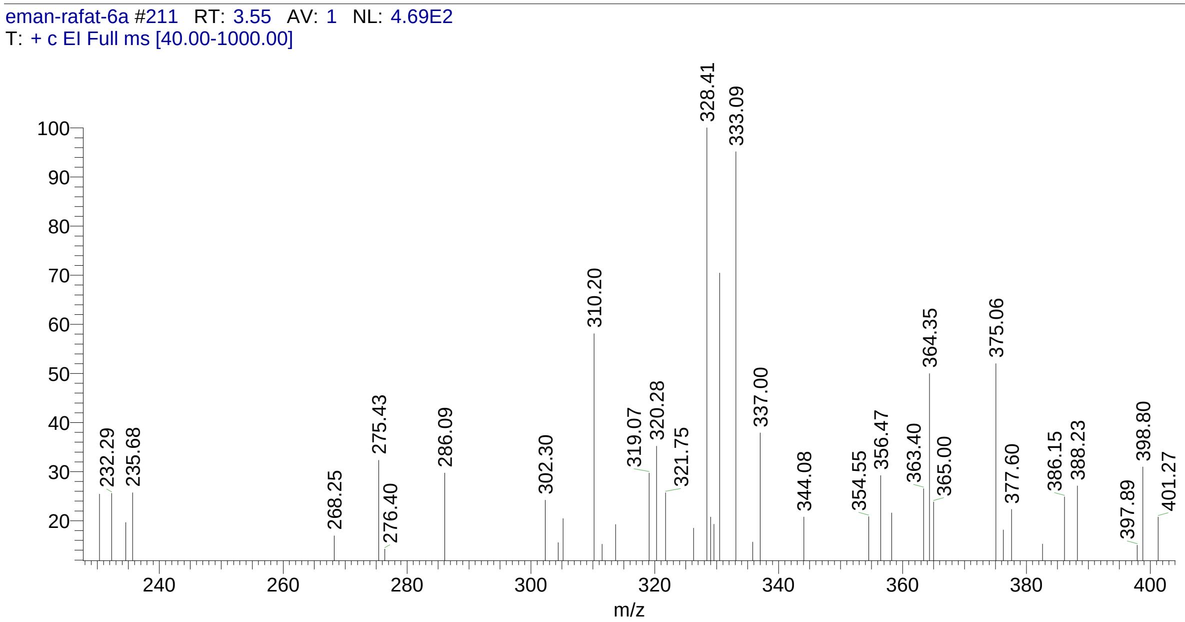
eman-rafat-6a #211 RT: 3.55 AV: 1 NL: 4.69E2
T: + c EI Full ms [40.00-1000.00]
328.41
333.09
310.20
375.06
364.35
337.00
275.43
286.09
320.28
319.07
321.75
232.29
235.68
268.25
276.40
302.30
344.08
354.55
356.47
363.40
365.00
377.60
386.15
388.23
397.89
398.80
401.27
m/z

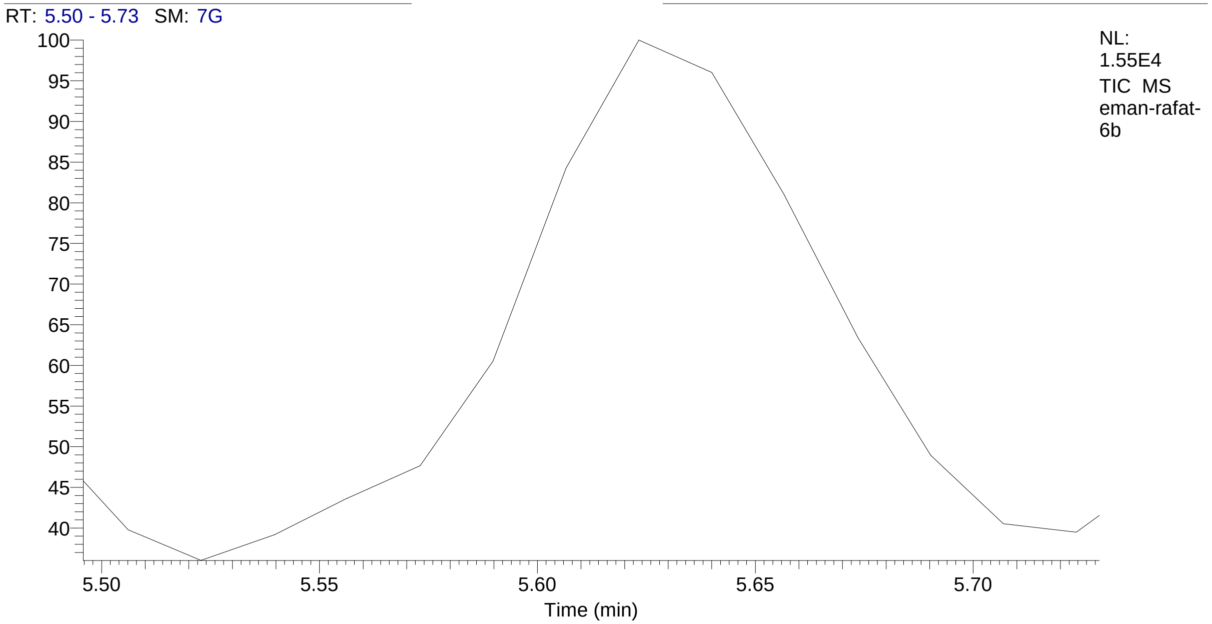
RT: 5.50 - 5.73 SM: 7G
NL: 1.55E4 TIC MS eman-rafat-6b
Time (min)

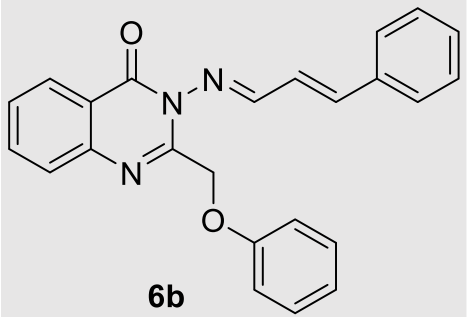
O
N
N
N
O
6b

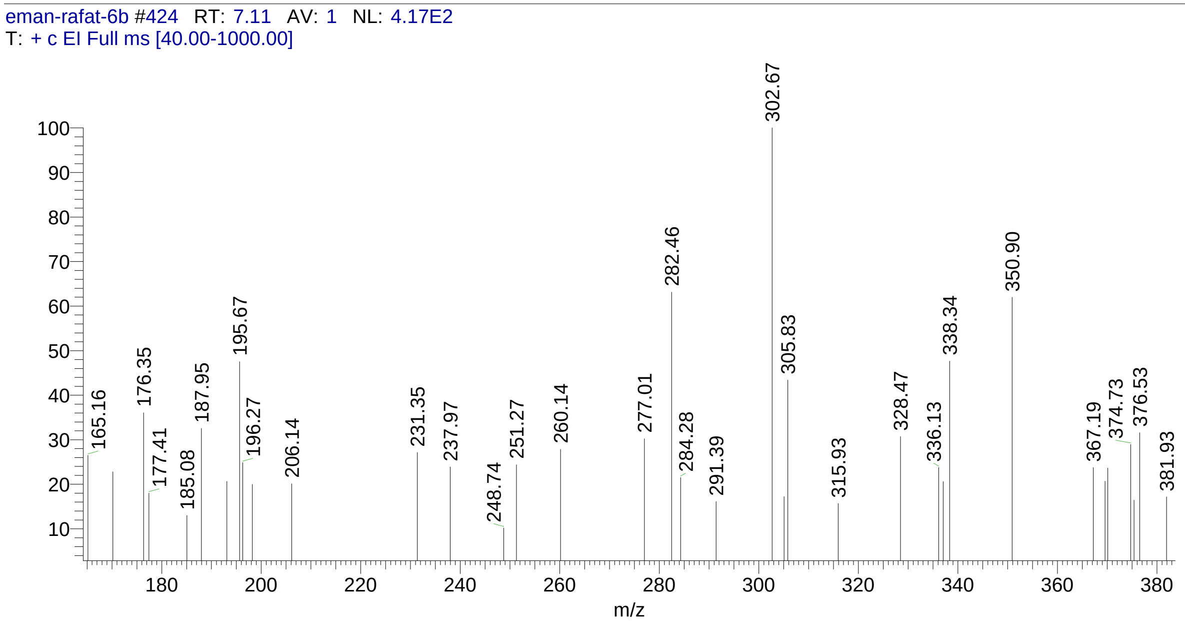
eman-rafat-6b #424 RT: 7.11 AV: 1 NL: 4.17E2
T: + c EI Full ms [40.00-1000.00]
302.67
282.46
350.90
338.34
305.83
195.67
176.35
187.95
165.16
177.41
185.08
196.27
206.14
231.35
237.97
248.74
251.27
260.14
277.01
284.28
291.39
315.93
328.47
336.13
367.19
374.73
376.53
381.93
m/z

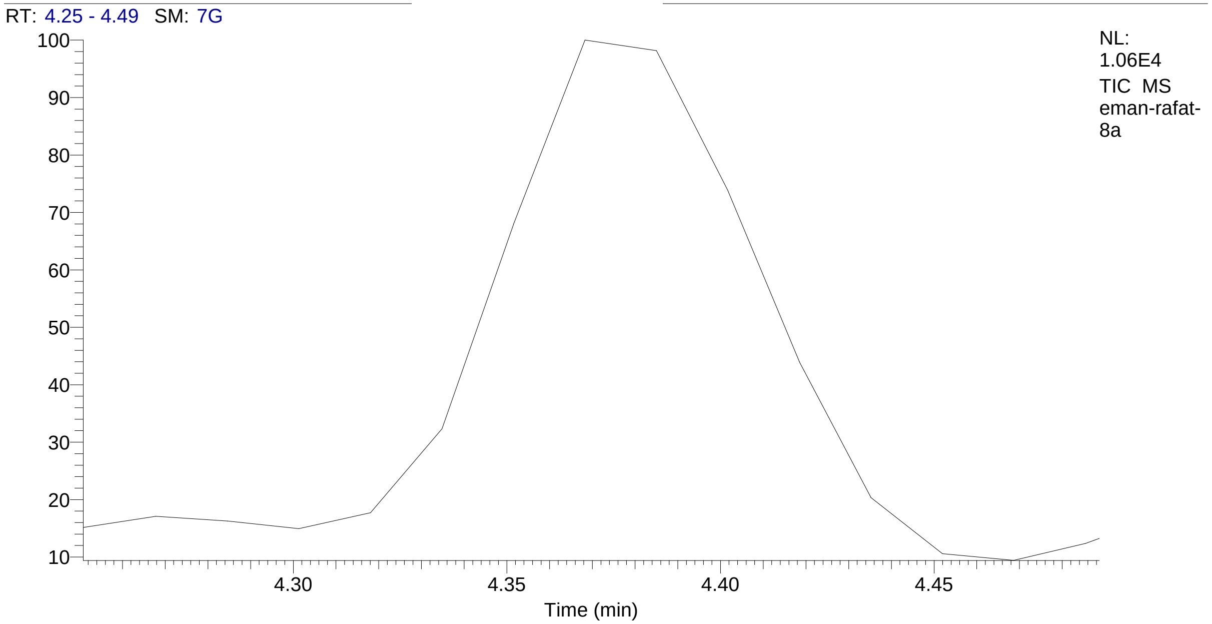
RT: 4.25 - 4.49 SM: 7G
NL: 1.06E4
TIC MS
eman-rafat-8a
Time (min)

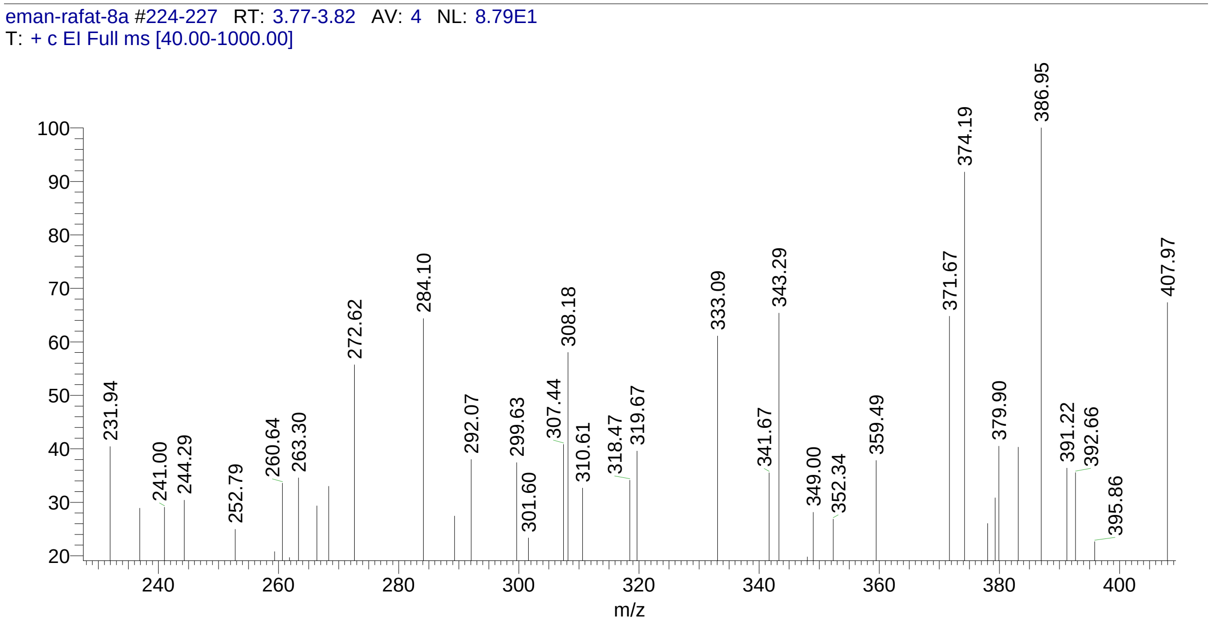
eman-rafat-8a #224-227 RT: 3.77-3.82 AV: 4 NL: 8.79E1
T: + c EI Full ms [40.00-1000.00]
231.94
241.00
244.29
252.79
260.64
263.30
272.62
284.10
292.07
299.63
301.60
307.44
308.18
310.61
318.47
319.67
333.09
341.67
343.29
349.00
352.34
359.49
371.67
374.19
379.90
386.95
391.22
392.66
395.86
407.97
m/z

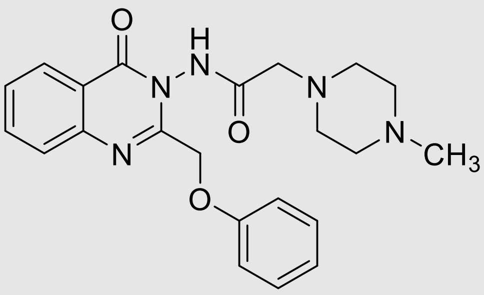
O
H
N
N
O
N
N
N
CH3
O

8a

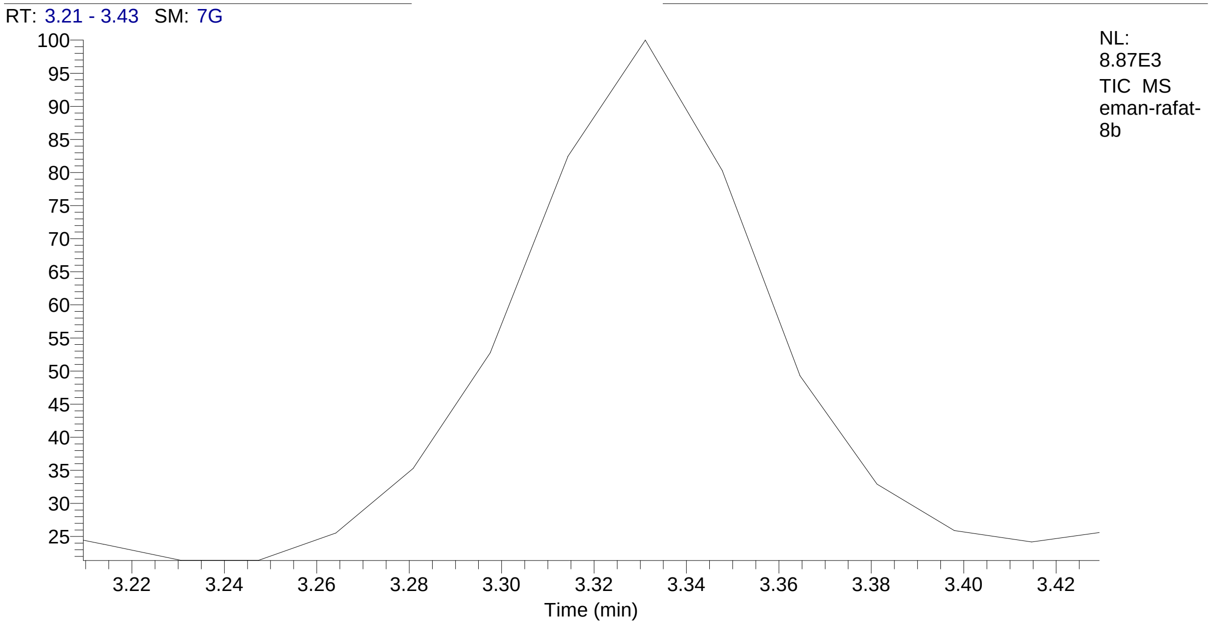
RT: 3.21 - 3.43 SM: 7G
NL:
8.87E3
TIC MS
eman-rafat-
8b
Time (min)

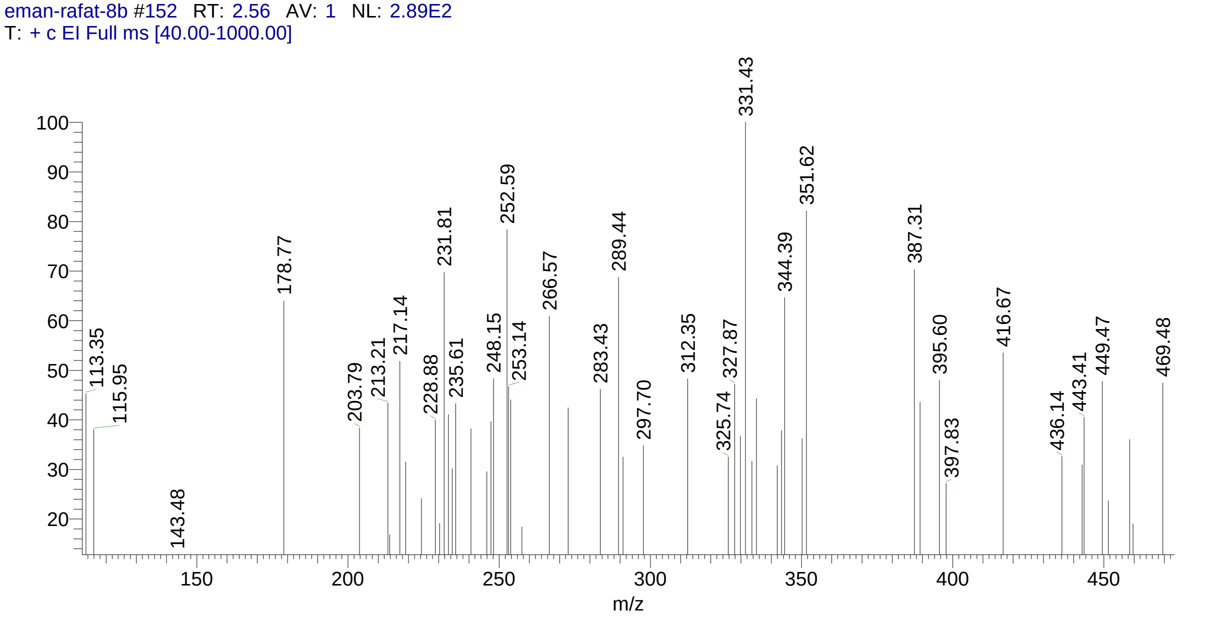
eman-rafat-8b #152 RT: 2.56 AV: 1 NL: 2.89E2
T: + c EI Full ms [40.00-1000.00]
331.43
351.62
252.59
231.81
289.44
387.31
344.39
178.77
266.57
416.67
469.48
449.47
443.41
436.14
395.60
397.83
312.35
327.87
325.74
297.70
283.43
253.14
248.15
235.61
228.88
217.14
213.21
203.79
143.48
115.95
113.35
m/z

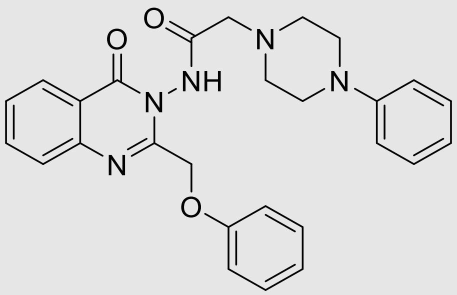

8b

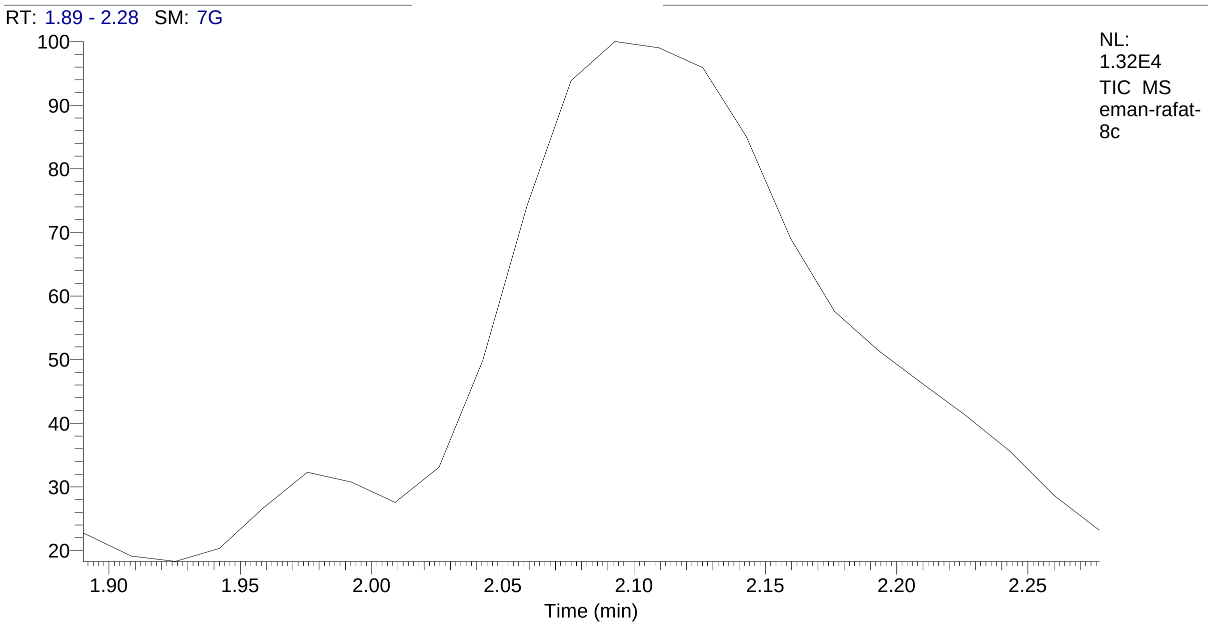
RT: 1.89 - 2.28 SM: 7G
NL:
1.32E4
TIC MS
eman-rafat-
8c
Time (min)

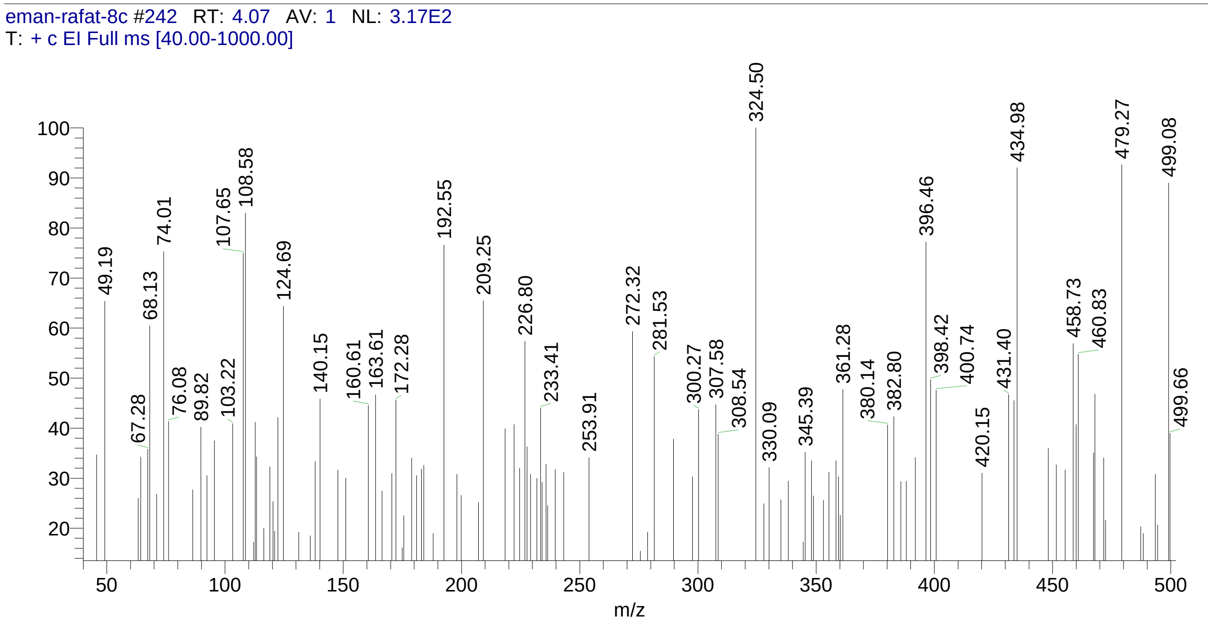
eman-rafat-8c #242 RT: 4.07 AV: 1 NL: 3.17E2
T: + c EI Full ms [40.00-1000.00]
324.50
434.98
479.27
499.08
108.58
107.65
192.55
74.01
396.46
209.25
49.19
124.69
68.13
272.32
226.80
458.73
460.83
281.53
361.28
398.42
400.74
431.40
140.15
160.61
163.61
172.28
233.41
300.27
307.58
308.54
76.08
89.82
103.22
67.28
253.91
330.09
345.39
380.14
382.80
420.15
499.66
m/z

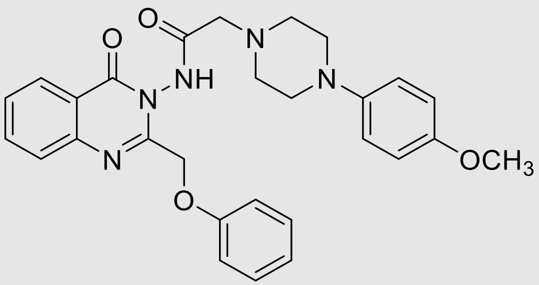
O
O
N
NH
N
N
N
O
OCH3
8c
